# Stapled ACE2 peptidomimetics designed to target the SARS‐CoV‐2 spike protein do not prevent virus internalization

**DOI:** 10.1002/pep2.24217

**Published:** 2021-01-08

**Authors:** Danielle C. Morgan, Caroline Morris, Amit Mahindra, Connor M. Blair, Gonzalo Tejeda, Imogen Herbert, Matthew L. Turnbull, Gauthier Lieber, Brian J. Willett, Nicola Logan, Brian Smith, Andrew B. Tobin, David Bhella, George Baillie, Andrew G. Jamieson

**Affiliations:** ^1^ School of Chemistry, University of Glasgow Glasgow UK; ^2^ MRC‐University of Glasgow Centre for Virus Research Glasgow UK; ^3^ Sir Michael Stoker Building Glasgow UK; ^4^ Centre for Translational Pharmacology Institute of Molecular Cell and Systems Biology, Davidson Building, University of Glasgow Glasgow UK

**Keywords:** peptidomimetic, protein‐protein interaction, stapled peptides, SARS‐CoV‐2, virus

## Abstract

COVID‐19 is caused by a novel coronavirus called severe acute respiratory syndrome‐coronavirus 2 (SARS‐CoV‐2). Virus cell entry is mediated through a protein‐protein interaction (PPI) between the SARS‐CoV‐2 spike protein and angiotensin‐converting enzyme 2 (ACE2). A series of stapled peptide ACE2 peptidomimetics based on the ACE2 interaction motif were designed to bind the coronavirus S‐protein RBD and inhibit binding to the human ACE2 receptor. The peptidomimetics were assessed for antiviral activity in an array of assays including a neutralization pseudovirus assay, immunofluorescence (IF) assay and in‐vitro fluorescence polarization (FP) assay. However, none of the peptidomimetics showed activity in these assays, suggesting that an enhanced binding interface is required to outcompete ACE2 for S‐protein RBD binding and prevent virus internalization.

## Graphical abstract

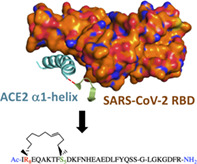

## 1 INTRODUCTION

Coronavirus disease 2019 (COVID‐19) has emerged as a severe pandemic with >42 million confirmed cases and 1.1 M deaths globally since its outbreak (WHO, October 25th, 2020).^[^ ^1^ ^]^ COVID‐19 is caused by the severe acute respiratory syndrome‐coronavirus 2 (SARS‐CoV‐2) positive‐strand RNA virus. The main symptoms of COVID‐19 infection include fever, continuous cough, difficulties in breathing and/or shortage of breath and loss of taste/smell. Elderly patients and individuals with pre‐existing medical conditions are at highest risk. Several candidate therapies are being assessed in on‐going clinical trials, including the ChAdOx1 vaccine,^[^ ^2^ ^]^ however no effective treatment for COVID‐19 currently exists and the current standard in care relies on supportive treatments.^[^ ^3^ ^]^ New therapeutics that directly target the virus are therefore urgently required to treat infected patients. As such, there is a clear and urgent requirement for the development of new, effective antiviral therapeutics.^[^ ^4^ ^]^

**FIGURE 1:**
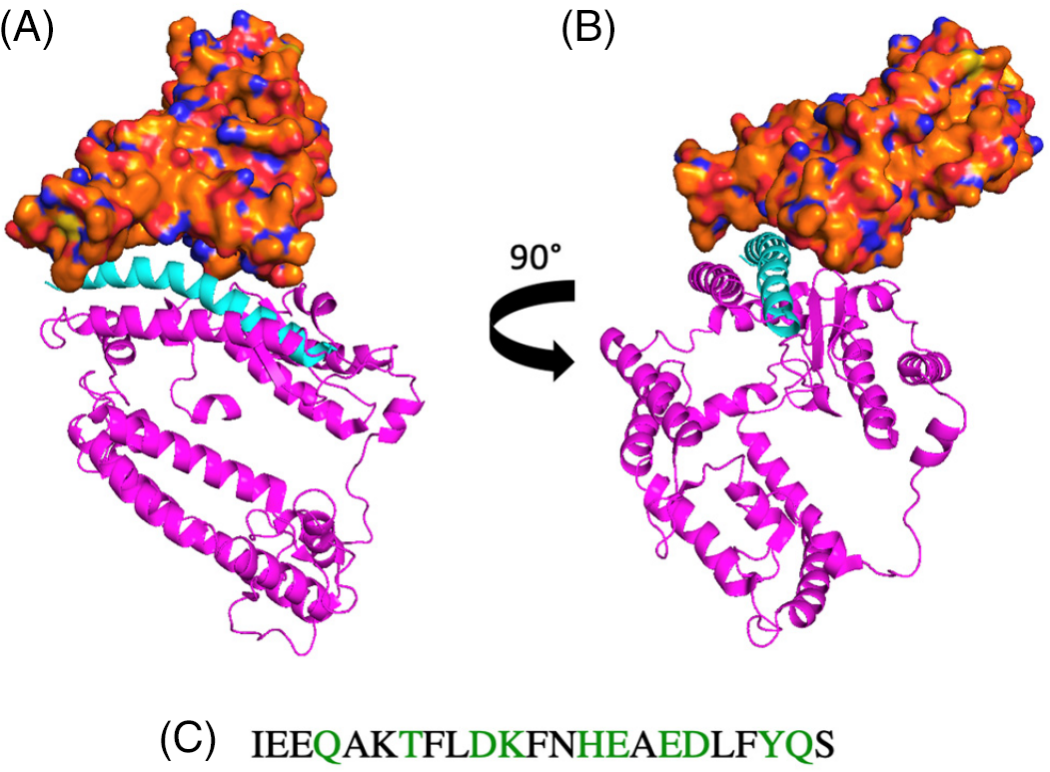
The structure of the SARS‐CoV‐2‐S receptor binding domain (RBD) (orange) and a segment of the ACE2 receptor (pink) (PDB: 6M07). A, Front view. B, Side view. α1 (cyan), appears to interact with the RBD of SARS‐CoV‐2 (orange). C, A section of the α1 peptide sequence, showing the interacting residues (green)

SARS‐CoV‐2 and SARS‐CoV infect cells via a critical protein‐protein interaction (PPI) between the SARS‐CoV spike glycoprotein (S‐protein) receptor binding domain (RBD) with the protease domain (PD) of the human cell surface receptor angiotensin‐converting enzyme 2 (ACE2).^[^ ^5^, ^6^ ^]^ Regulation of PPIs using traditional small molecules is extremely challenging due to the relatively large (typically ∼1500‐3000 Å^2^) and dynamic nature of the PPI interface with few, if any, binding pockets.^[^ ^7^ ^]^ As such, small molecule regulation of the S‐protein RBD‐ACE2 PD PPI is unlikely to be a successful approach. The recently reported cryo‐EM structures of SARS‐CoV‐2 S‐protein RBD and full length human ACE2 receptor have revealed that the molecular recognition event is predominantly mediated by ACE2 PD helix α1, which makes several important amino acid side‐chain interactions with the SARS‐CoV‐2 RBD (Figure 1).^[^ ^8^, ^9^, ^10^, ^11^ ^]^ Regulation of helix mediated PPIs such as this one is achievable using peptides.^[^ ^12^ ^]^ Previous studies in this field have included the use of medium length linear peptides derived from ACE2, which demonstrated micromolar affinity binding of the SARS‐CoV‐2 S‐protein RBD,^[^ ^13^ ^]^ targeting RBD for degradation,^[^ ^14^ ^]^ and inhibition of ACE2‐receptor mediated host cell SARS‐CoV pseudovirus entry *in vitro*.^[^ ^15^ ^]^ However, linear peptides are generally poor lead compounds for drug discovery due to their poor DMPK properties, including blood plasma stability. A challenge therefore exists to design molecules that occupy the chemical space large enough to modulate this PPI and yet small enough to have suitable drug‐like DMPK properties.

Stapled peptides have recently come of age as tool compounds for the regulation of PPIs.^[^ ^16^ ^]^ These peptidomimetics incorporate a hydrocarbon‐bridge that conformationally constrains the peptide into its α‐helical bioactive conformation.^[^ ^17^, ^18^ ^]^ They have been shown in numerous biologically relevant examples (e.g., p53/MDM2, Bcl2 and Aurora‐A/TPX2) to have excellent binding affinity, selectivity and also favorable DMPK properties.^[^ ^19^ ^]^

Soluble ACE2 has been investigated as a potential therapeutic, acting as a competitive interceptor of SARS‐CoV‐2 and preventing viral binding to cells.^[^ ^20^ ^]^ Recent studies have shown that human recombinant soluble ACE2 (hrsACE2) have shown promise for treating severe COVID‐19.^[^ ^21^, ^22^ ^]^

The aim of this work was to develop stable, conformationally constrained stapled analogues of the ACE2 PD helix α1 peptide that would bind to SARS‐CoV‐2 S‐protein RBD and prevent interaction with endogenous ACE2 receptors. Here we describe the design, synthesis and biological evaluation of a series of stapled peptide ACE2 mimetics.

## 2 EXPERIMENTAL

### 2.1 Peptide synthesis and characterization

Peptides were synthesized using a Fmoc/^t^Bu strategy on a microwave assisted solid‐phase peptide synthesizer (Biotage Alstra) on a 0.1 mmol scale using Tentagel S RAM resin. *N*,*N*′‐Diisopropylcarbodiimide (DIC)/ 2‐Cyano‐2‐(hydroxyimino)acetic acid ethyl ester (Oxyma Pure) coupling conditions were employed at 90 °C for 2 minutes. For stapled peptides, commercially available unnatural amino acids were incorporated in the sequence [(*R*)‐N‐Fmoc‐*α*‐(7‐octenyl)alanine (R_8_) and (*S*)‐N‐Fmoc‐*α*‐(4‐pentenyl)alanine (S_5_)]. Macrocyclisation was achieved on‐resin by ring‐closing metathesis (RCM) using Grubbs first Generation Catalyst (20 mol%).^[^17] The cyclisation reactions were allowed to proceed for 2 hours and were then repeated a second time using fresh reagents. Upon cyclisation, a mixture of cis and trans isomers of the peptides formed as assessed by LC‐MS of the crude mixture following cleavage test (3 mg resin). The cis and trans isomers had similar retention times making purification difficult, however the trans isomer of each peptide could be isolated by reverse phase high‐performance liquid chromatography (RP‐HPLC). All peptides were characterized by analytical‐RP‐HPLC and mass spectrometry (see Tables S1‐S5). Analogues of each peptide were synthesized with an N‐terminal fluorescein label. Fluorescein isothiocyanate isomer I (FITC) was reacted with the amine of a 6‐aminohexanoic acid linker to space the label from the peptide and prevent thiohydantoin formation.

## 3 RESULTS AND DISCUSSION

### 3.1 First generation peptides

SARS‐CoV‐2 enters cells through the molecular interaction of the spike protein RBD with the cell entry receptor ACE2.^[^ ^6^ ^]^ ACE2 is an essential regulator of renin‐angiotensin‐aldosterone system (RAAS) activity, which is a vital regulator of cardiovascular and renal function. Developing mimics of ACE2 as decoys therefore presents a potential therapeutic strategy to prevent viral infection. The Cryo‐EM structure of the SARS‐CoV‐2‐S receptor binding domain (RBD) and the full length human ACE2 receptor provides information on the key amino acid residues involved in the PPI, and thus provides the opportunity to rationally design therapeutic peptides based on the interaction hotspot constellation of ACE2 amino acids.^[^ ^9^, ^11^ ^]^ Indeed, the structure reveals the ACE2 interaction hotspot as an alpha‐helical motif, α1 (cyan) (Figure 1A,B). From inspection of the structure, α1 helix appears to interact with the RBD and the residues involved can be clearly identified (Figure 1C, showing the interacting residues in green). By analyzing this crystal structure, we considered resides Gln_24_, Thr_27_, Asp_30_, Lys_31_, His_34_, Glu_35_, Glu_37_, Asp_38_, Tyr_41_, Gln_42_ of ACE2 to be key for this PPI when designing our peptide series.

There have been several reports based on the linear peptide fragment of the ACE2 PD α1 helix^[^ ^13^, ^23^ ^]^ Both biophysical and pseudoviral techniques have been used to assess the binding and activity, revealing that the peptides showed micromolar affinity and anti‐viral activity.

The aim of our work was to develop a series of conformationally constrained helical ACE2 peptides and to investigate their ability to bind SARS‐CoV‐2 S‐protein RBD, disrupt the RBD‐ACE2 PPI and prevent viral infection. Stapling peptides induces the helical bioactive conformation and pays the entropic penalty of folding, improving the biophysical properties and potentially the binding affinity vs the native peptide. In parallel, we also set out to produce truncated stapled peptides covering the full 23‐mer sequence, in an attempt to induce the bioactive conformation at key interaction residues and therefore enhance binding affinity with a shorter sequence.

**FIGURE 2:**
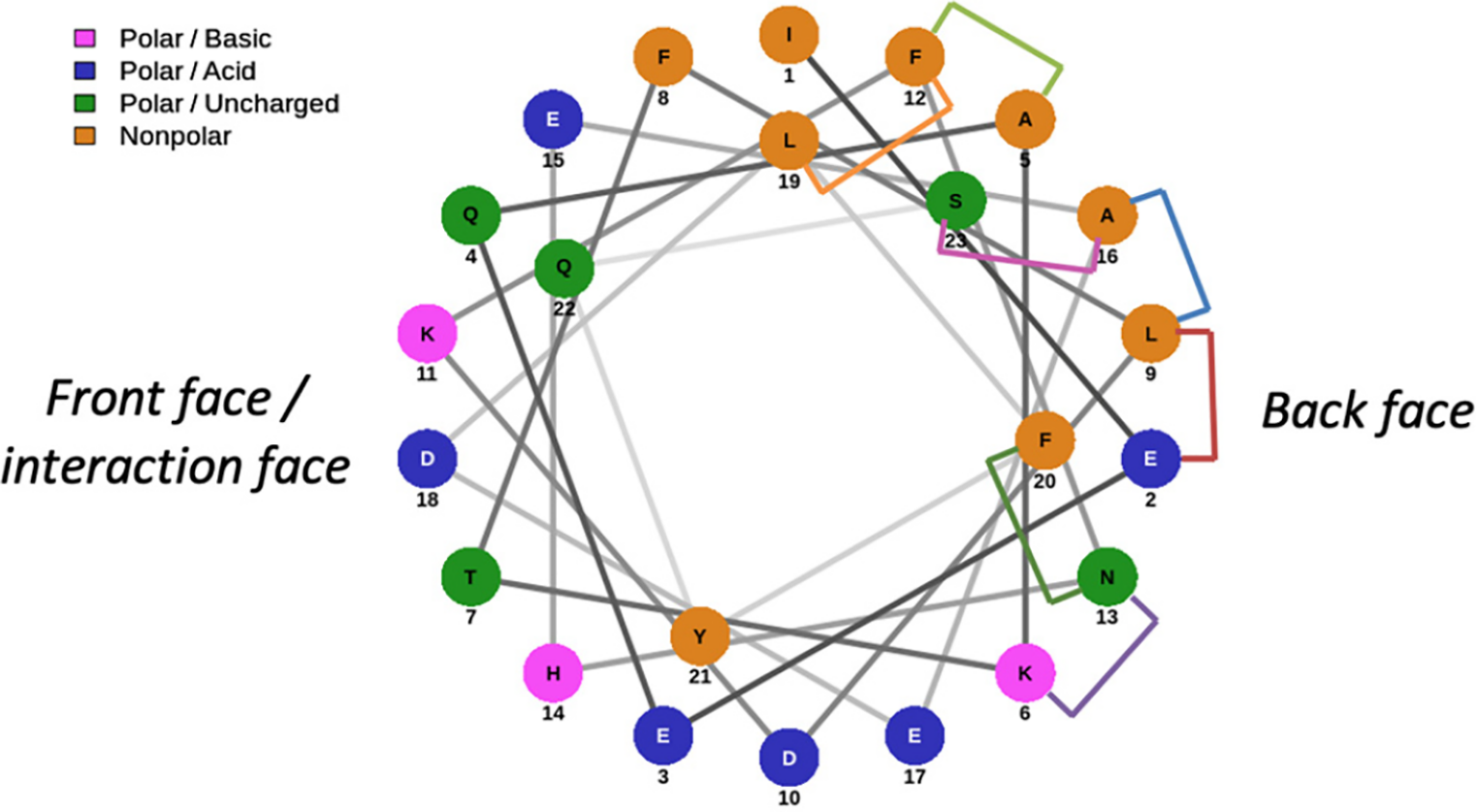
A helix wheel showing the positioning of the amino acids in the sequence with respect to the helix structure. The right side shows the amino acids on the back, nonbonding face of the helix, where we elected to position our staples (colored bridges)

The ACE2 protein domain (PD) α1 helix has a bend at His_34_ which should be considered when selecting the position of a staple. We created a helix wheel to decide on the staple positioning within the sequence (Figure 2). The constellation of amino acids on the left‐hand side, or front face of the helix wheel are the residues involved in the interaction with ACE2. On the right‐hand side of the helix wheel are the residues not involved in the binding event and that will be projected into solution.

We placed a staple at every available position on the back face of the helix, avoiding the interaction domain. Peptides were designed to incorporate either *i*, *i* + 7 or *i*, *i* + 4 hydrocarbon‐staples that would conformationally constrain the peptide over approximately two turns or one turn of the helix, respectively.

A range of peptidomimetic analogues with *i*, *i* + 7 staples (Table 1, **2**‐**8**) were designed to test this positioning, for comparison with the native peptide **1**. Staples were placed either side of the histidine, as well as across it, to test the importance of this bend within the helix. Truncated analogues were also designed based on the residues that interact with the RBD. Three truncated peptides were selected, and *i*, *i* + 4 and *i*, *i* + 7 stapled analogues designed (Table 1, **9**‐**15**). A peptide with a scrambled sequence was synthesized as a negative control. A second negative control incorporating an *i*, *i* + 7 hydrocarbon staple on the binding face of the peptide was produced, which should not bind to the SARS‐CoV‐2‐RBD.

**TABLE 1:** Peptides designed based on the ACE2 PD a1 helix **1**. A staple scan was conducted on the back face of the helix, ranging from N‐terminal stapling positions to C‐terminal stapling

Name	Sequence
Native (1)	IEEQAKTFLDKFNHEAEDLFYQS
2	IR_8_EQAKTFS_5_DKFNHEAEDLFYQS
3	IEEQR_8_KTFLDKS_5_NHEAEDLFYQS
4	IEEQAR_8_TFLDKFS_5_HEAEDLFYQS
5	IEEQAKTFR_8_DKFNHES_5_EDLFYQS
6	IEEQAKTFLDKR_8_NHEAEDS_5_FYQS
7	IEEQAKTFLDKFR_8_HEAEDLS_5_YQS
8	IEEQAKTFLDKFNHER_8_EDLFYQS_5_
9	HEAEDLFYQS
10	HES_5_EDLS_5_YQS
11	IEEQAKTFLDKFNHE
12	IEEQR_8_KTFLDKS_5_NHE
13	TFLDKFNHEAEDL
14	TFR_8_DKFNHES_5_EDL
15	TS_5_LDKS_5_NHEAEDL
Scrambled negative control	FHTSEYDEQNEIEAAQLFKDFLK
Stapled negative control	IEEQAKR_8_FLDKFNS_5_EAEDLFYQS

**FIGURE 3:**
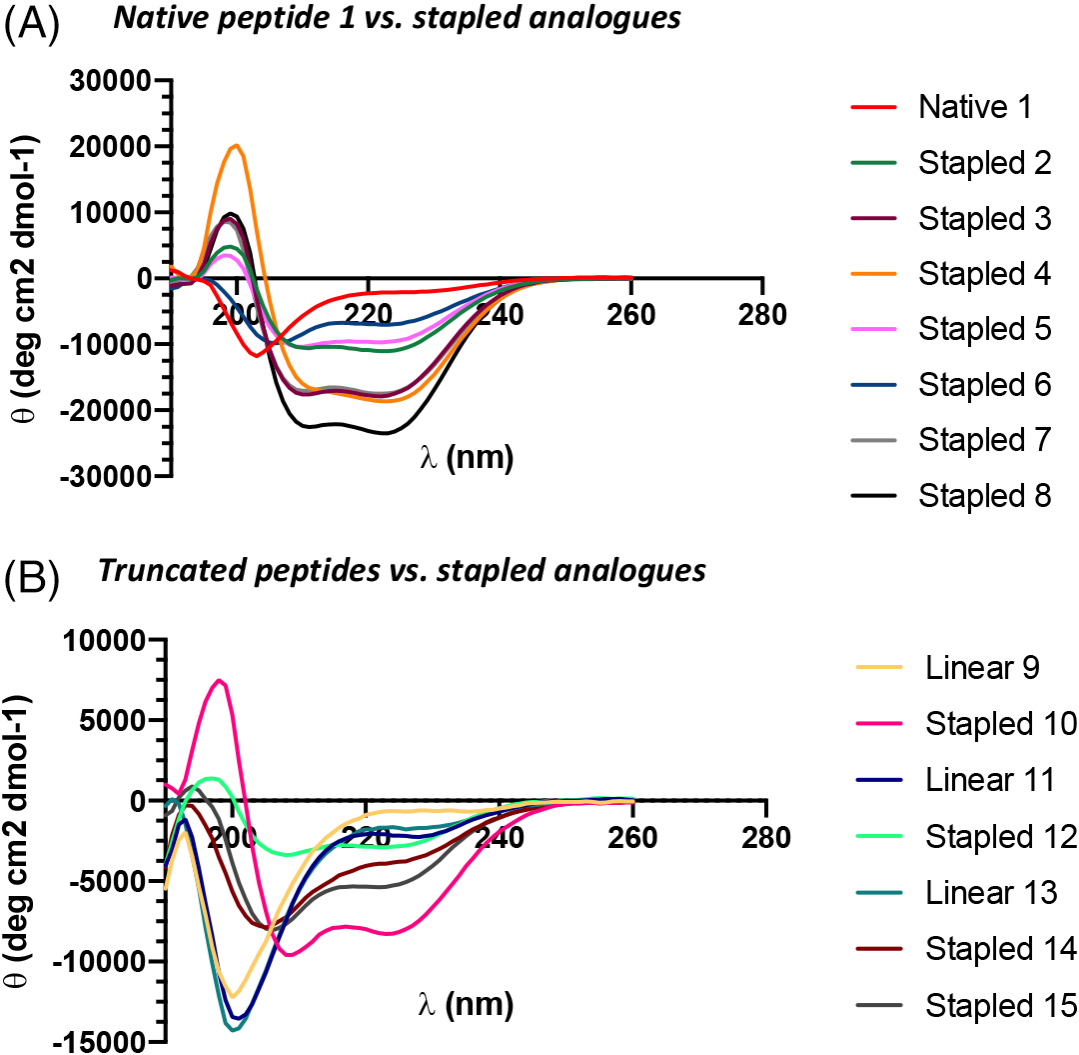
Circular dichroism spectra of the native ACE2 peptide and the stapled peptidomimetics. A, CD data of the native peptides compared to the stapled analogues. B, CD data of the truncated analogues and their stapled variants. Conditions: peptides 100 μM in PBS, pH 7.4. Spectra recorded between 190 and 260 nm

Conformational analysis was then achieved using circular dichroism (CD) spectroscopy to assess the effect of the staples on the structure of the peptides. Each peptide was analyzed at 100 μM (Figure 3) in phosphate buffered saline (PBS, pH 7.4). CD spectra were measured from 190 to 260 nm in order to observe the characteristic α‐helical minima at 208 nm and 222 nm.^**[**^ ^24^ ^]^

CD experiments confirmed that hydrocarbon stapling stabilizes the sequence into an α‐helix, relative to peptide **1** which is random coil (Figure 3A). The most helical peptidomimetic is peptide **8**, suggesting that positioning of the staple *C*‐terminal is favorable. This is mirrored with the truncated peptide series (Figure 3B), where **10**, the only truncated peptidomimetic with its staple *C*‐terminal to His_34,_ exhibits the greatest helicity. The raw CD data was converted to molar residue ellipticity (MRE) and the value at 222 nm was used to calculate the % helicities using Equation S1 (see SI). The most helical peptide was stapled peptide **8** with 72% helicity, in comparison to the native peptide **1** with 9% helicity (see SI for all peptide % helicities—Table S6). As such, the hydrocarbon conformational constraints are clearly effective at inducing the helical bioactive conformation of α1.

Assays were then selected to assess the ability of the peptidomimetics to bind SARS‐CoV‐2 RBD, disrupt the PPI with ACE2 and neutralize viral infectivity.

#### 3.1.1 Pseudovirus inhibition assay

**FIGURE 4:**
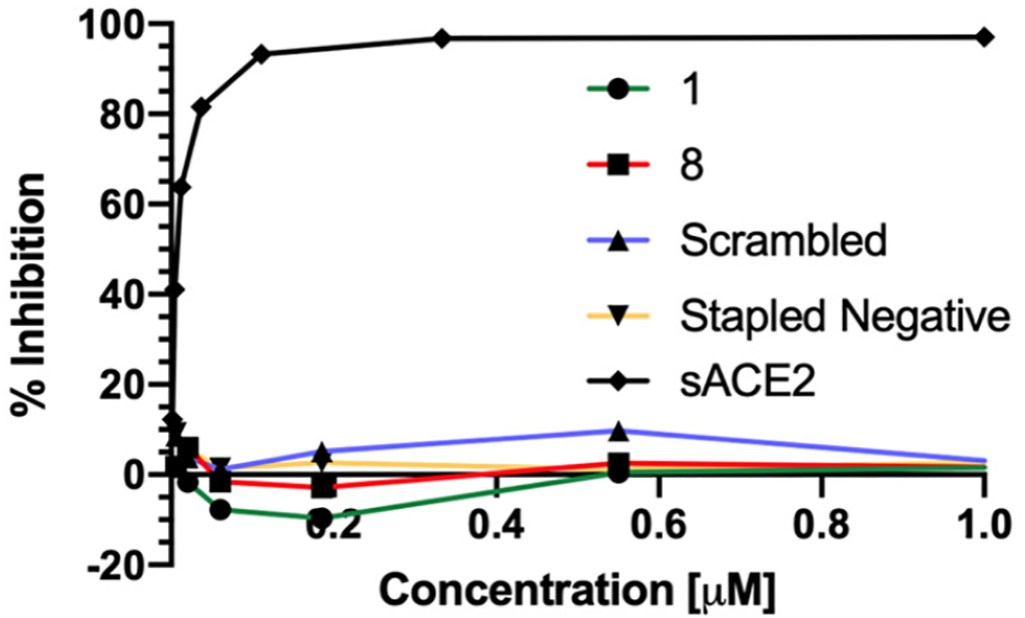
Neutralization data for the positive control—soluble ACE2, and data for peptide 1, 8 and the negative controls

To determine if the stapled peptidomimetics can inhibit SARS‐CoV‐2 entry *in vitro*, pseudovirus inhibition assays were utilized. Pseudovirus consisting of a HIV core, with a luciferase reporter gene, and Wuhan‐Hu‐1 strain of the SARS‐CoV‐2 spike was used. Peptides were incubated with the pseudovirus for 1 hour before addition of 293 T ACE2 expressing cells. After a 48‐hour incubation luciferase activity was measured and inhibition was calculated as a percentage against DMSO negative controls. Soluble ACE2 (sACE2) protein has previously been shown to effectively act as a decoy, bind to the SARS CoV‐2 spike protein and prevent viral infection. sACE2 was thus used as a positive control to validate the assay (Figure 4). It can be seen that the presence of ACE2 inhibited viral entry into cells (IC_50_ = 5.36 nM). The most helical peptidomimetic **8**, native α1 peptide **1** and the two negative control peptides (scrambled and stapled) were tested. None of these compounds including stapled peptide **8** inhibited viral entry at up to 5 mM concentration (Figure 4). The full series of peptides **1**‐**15** were also assessed however did not show any activity in this assay (Figure S1).

#### 3.1.2 Immunofluorescence (IF) assay

**FIGURE 5:**
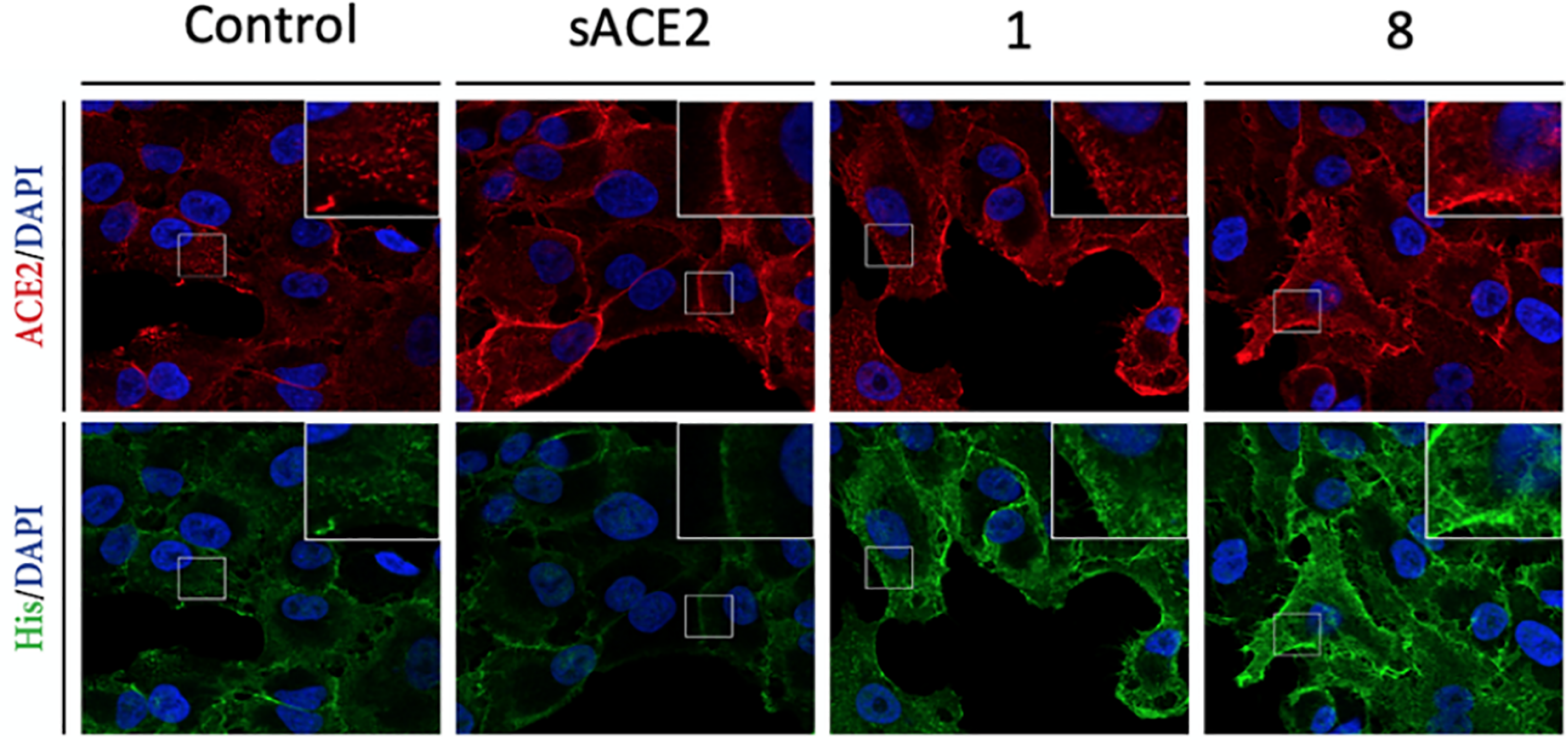
Immunofluorescence data shows that cells treated with ACE2 can block the entry of His‐tagged SARS‐CoV RBD (green). The cells treated with peptides 1, 8 show entry of the His‐tagged SARS‐CoV RBD into cells. Stapled peptides (10 μM concentration) or soluble hACE2 (~100 nM) were incubated with 50 nM S_RBD_ for 30 min at 37 °C and later added to A549 cells for 3 hours. Scale bar, 20 μm

To directly probe the ability of the peptidomimetics to disrupt the SARS‐CoV‐2 RBD/ACE2 PPI in a cellular context, yet avoiding the complexity of a pseudovirus assay, we designed an immunofluorescence assay. The lung cancerogenic cell line A549 was chosen as an alveolar Type II pulmonary epithelium cell model because SARS‐CoV‐2 predominately infects the host via the lung. Incubation of a commercial His‐tagged S‐RBD protein was able to stimulate the internalization of ACE2 overexpressed in A549 cells (Figure S2). Furthermore, S‐RBD protein colocalized with the ACE2 endocytic vesicles, which suggests that the entry of both proteins as a complex. Importantly, no S‐RBD signal was observed in control A549 cells, indicating that the internalization is ACE2‐dependent (Figure S2). Preincubation of the S‐RBD protein with soluble ACE2 before addition to the cells partially reduced the number of ACE2 vesicles in the cytoplasm (Figure 5). However, treatment with native α1 peptide **1** and peptidomimetic **8** showed a similar ACE2 internalization response than with the control conditions (Figure 5).

These results show that none of these peptides are able to bind the S‐RBD and disrupt the SARS‐CoV‐2 RBD/ACE2 PPI. ACE2 evidently has significantly higher binding affinity for the S‐RBD than the stapled peptidomimetics. With both the pseudo‐virus and IF assays indicating that the peptidomimetics lack the ability to disrupt the ACE2/SARS‐CoV‐2 PPI, we then turned to a biophysical assay to measure the binding affinity of the peptidomimetics for the SARS‐CoV‐2 S‐protein RBD.

#### 3.1.3 Fluorescence polarization (FP) assay

**FIGURE 6:**
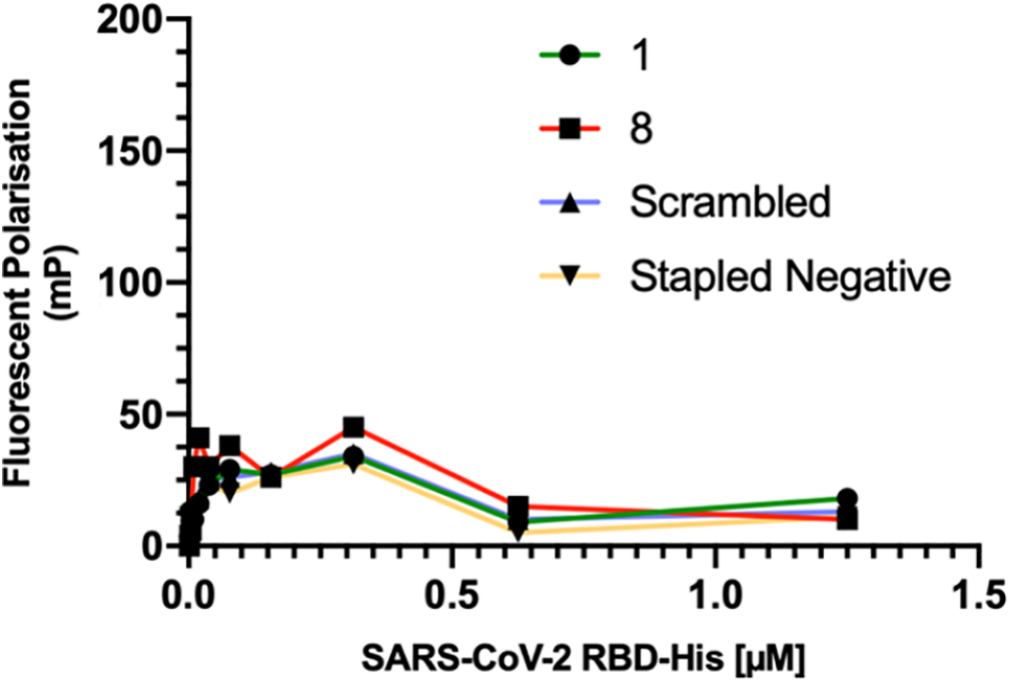
Direct binding assay (fluorescent polarization (FP) data for peptide **1**, **8** and the negative controls—Fluorescent polarization measurements taken at 30 minutes post‐incubation at room temperature

FP was used to compare the direct binding affinities of N‐terminal fluorescein labeled analogues of α1 peptide **1** and the peptidomimetics **1**‐**15** with S‐RBD. Figure 6 shows the comparison between the native peptide **1**, stapled peptide **8**, along with the negative controls No fluorescent positive control is currently available. Increasing concentrations of S‐RBD were incubated with 100 nM final concentration of N‐terminally fluorescein labeled ACE2 peptides. Fluorescent polarization measurements were taken at 30 minutes (Figure 6 and S3), 60 minutes (Figure S4), 120 minutes (Figure S5), 240 minutes (Figure S6) and overnight (Figure S7) post‐incubation at room temperature. None of the peptides bound to S‐RBD at up to 1.25 μM concentration.

A *C*‐terminally fluorescein labeled peptide was also synthesized, to test whether the positioning of the fluorophore affected binding (Figure S8). The *C*‐terminally labeled peptide also did not show any binding. The FP data shows that all of the fluorescein labeled ACE2 peptides direct binding “curves” were similar to the ACE2 scrambled negative control, indicative of no binding with S‐RBD being observed.

### 3.2 Second generation peptides

As our initial peptide designs did not have any detectable binding affinity or activity, we next designed a series of longer peptides (Table 2). The critical determinants of SARS‐CoV S‐protein interaction with hACE2 required for cell entry have previously been reported.^**[**^ ^15^ ^]^ A 31‐mer peptide was prepared that incorporates the α1 helix linked to a hACE2 loop sequence that includes a lysine residue that makes an electrostatic interaction with the RBD. This peptide was shown to have potent activity (IC_50_ 100 nM) in a SARS‐CoV pseudovirus neutralization assay.

**TABLE 2:** A second generation array of peptides were designed based on previous work for SARS‐CoV^[^ ^15^ ^]^and a mutant peptide was included

G‐link	IEEQAKTFLDKFNHEAEDLFYQSS‐G‐LGKGDFR
D30E	IEEQAKTFLEKFNHEAEDLFYQSS‐G‐LGKGDFR
G‐link stapled	IR_8_EQAKTFS_5_DKFNHEAEDLFYQSS‐G‐LGKGDFR
Mutant Peptide	IEEQAKYFLEWFNPEAEDLFYLSS‐G‐FGKGDFR

**FIGURE 7:**
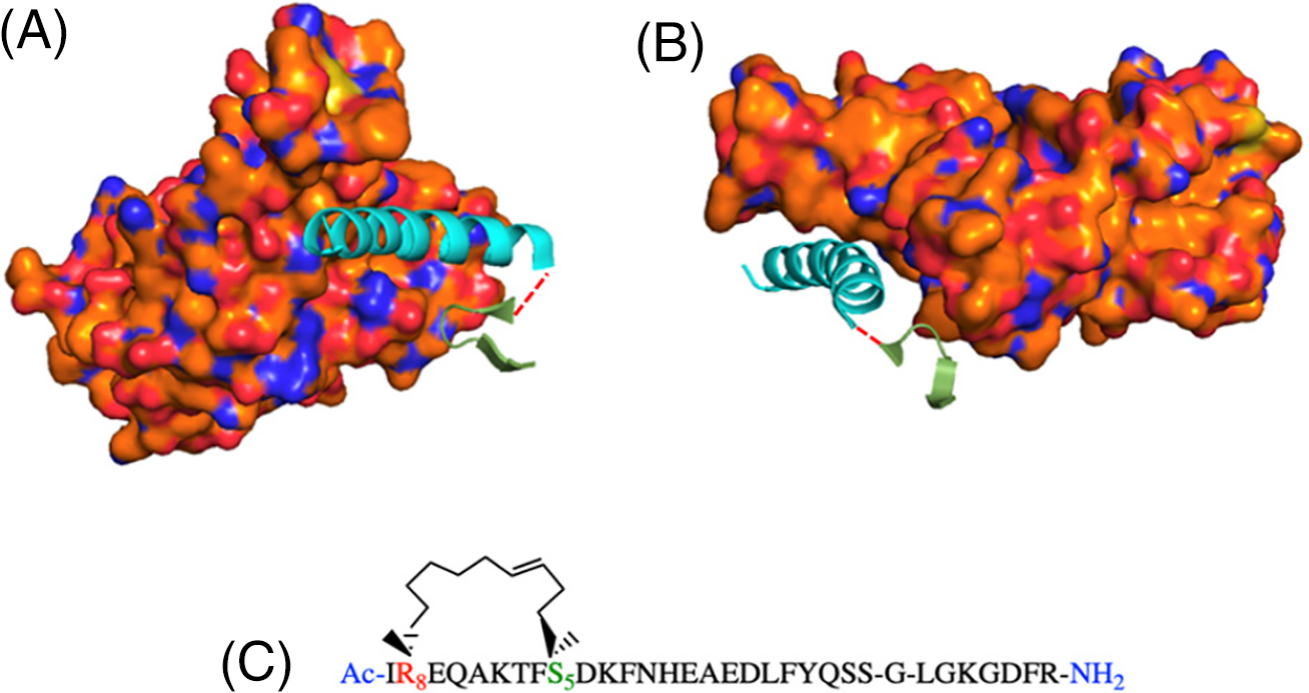
The ACE2 PD helix α1 (cyan) plus the discontinuous fragment (green). A, Front view. B, Side view. In previous work by Han *et al*. on SARS‐CoV, the peptide fragments were joined by a glycine (dashed red line). C, The longer G‐link peptides were conformationally constrained using an i, i + 7 alkene staple. (PDB: 6M07)

The RBD/ACE2 binding interfaces of SARS‐CoV and SARS‐CoV‐2 are similar^**[**^ ^25^ ^]^ and so we prepared this longer peptide (**G‐Link**) and assessed its activity in our SARS‐CoV‐2 pseudovirus assay. In addition, an analogue of this peptide with an amino acid substitution (**D30E**) previously reported to enhance binding affinity of ACE2 for the RBD was prepared.^**[**^ ^26^ ^]^ To constrain the helical bioactive conformation of the α1 sequence a stapled peptide analogue of this longer peptide was also synthesized (Table 2 and Figure 7C).

Finally, an analogue of this extended G‐link peptide was designed based on a series of amino acid mutations previously reported in the literature (Table 2).^**[**^ ^14^, ^27^, ^28^ ^]^ Conformational analysis using CD demonstrated that the native **G‐link** peptide adopts a random coil conformation. However, upon stapling the α1 component of this sequence an enhancement in α‐helical character was observed (Figure S9).

These second‐generation peptides were tested in the same neutralization, immunofluorescence and FP binding assays, again with soluble ACE2 as a positive control.

**FIGURE 8:**
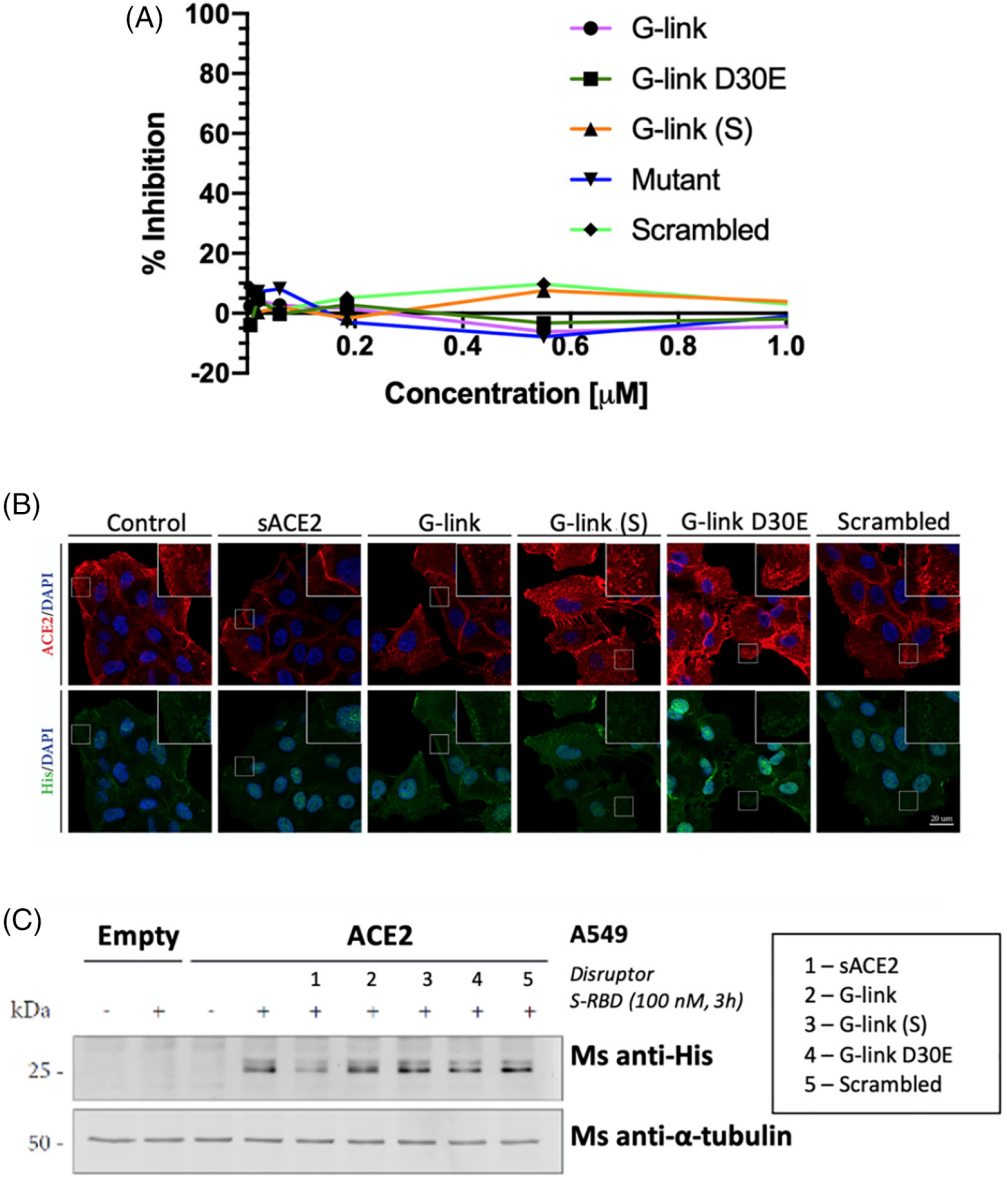
A, Neutralization data for the second generation peptides. B, Immunofluorescence data shows that cells treated with ACE2 can block the entry of His‐tagged SARS‐CoV RBD (green). The cells that were treated with the second gen. Peptides showed entry of the His‐tagged SARS‐CoV RBD into cells. Stapled peptides (10 μM concentration) or soluble hACE2 (100 nM) were incubated with 100 nM S‐RBD for 30 minutes at RT and later added to A549 cells overexpressing ACE2 for 3 hours. S‐RBD induces ACE2 internalization but recognition by His antibody is very weak. Scale bar, 20 μm. C, The Western Blot indicates that the peptides show no ability to block entry of the SARS‐CoV‐2 RBD

These longer peptides also showed no inhibition in the pseudovirus neutralization assay (A) or the immunofluorescence assay (B) (Figure 8). A western blot (WB) also indicated that the peptides show no ability to block entry of the SARS‐CoV‐2 RBD (Figure 8C), yet the positive control, sACE2, has the ability to block entry.

**FIGURE 9:**
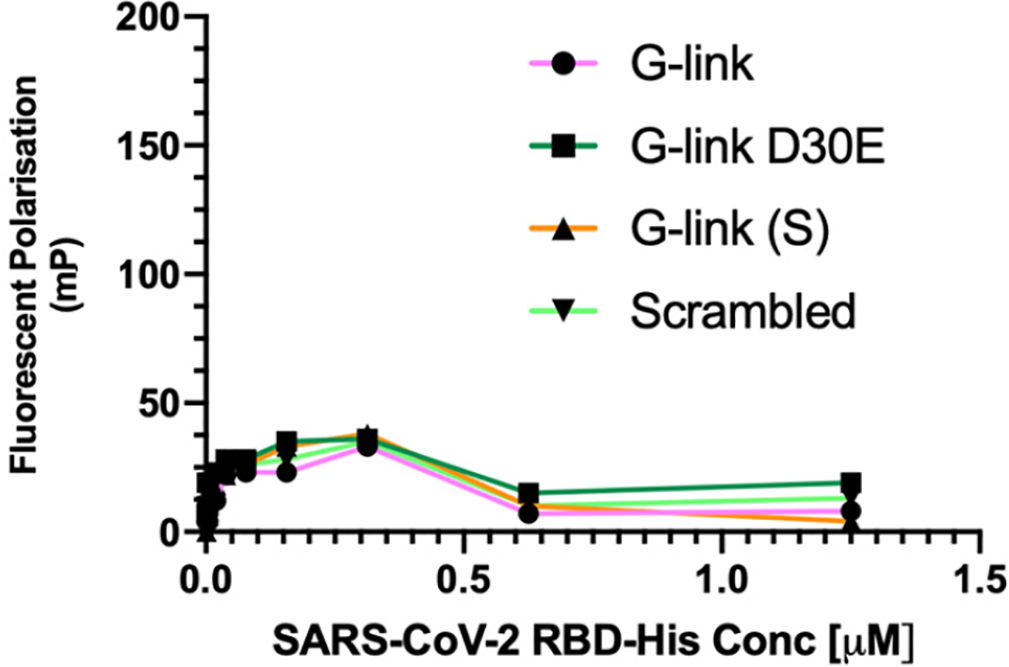
Direct binding assay (fluorescent polarization (FP) data for the second generation peptides—Fluorescent polarization measurements taken at 30 minutes post‐incubation at room temperature

FP was then used to assess if the second generation G‐link peptides bind to isolated, soluble RBD protein. Again, we were surprised that no binding was observed up to 1.25 μM concentration (Figure 9). FP was then used to assess if the second generation G‐link peptides bind to isolated, soluble RBD protein. Again, we were surprised that no binding was observed up to 1.25 μM concentration (Figure 9).

## 4 CONCLUSIONS

We have designed and synthesized a series of peptidomimetics based on the α1 helix interaction hotspot of native ACE2 PD. Our aim was to develop conformationally constrained peptides that would prevent viral infection through disrupting the SARS‐CoV‐2/ACE2 PPI. The preparation of 13 different stapled peptidomimetic analogues and 2 mutant peptides was successfully achieved using microwave assisted SPPS. Peptide stapling effectively constrained the helical structure in solution as confirmed by CD. However, none of these peptides showed activity in a neutralization assay. Furthermore, no evidence of binding to the target SARS‐CoV‐2 S‐protein RBD was detected in either immunofluorescence or in‐vitro fluorescence polarization assays.

While preparing this manuscript, Curreli *et al*. reported the design and synthesis of double stapled peptide analogues also based on native ACE2 PD α1.^[^ ^23^ ^]^ We also synthesized double stapled analogues of the α1 helix, however these were isolated as crude mixtures of cis/trans alkene isomers and when tested in the pseudovirus inhibition assay and they showed no anti‐viral activity (data not shown). In their studies, like us, they confirmed that the peptide designed by G. Zhang *et al*. did not show any binding. However, a pseudoviral assay determined that the most helical double stapled peptides did inhibit viral entry, suggesting that double stapling is a viable approach for inducing α1 helicity. Recently there have also been reports of miniprotein inhibitors of the SARS‐CoV‐2/ACE2 PPI with picomolar RBD binding affinity and comparable activity with ACE2 in cell‐based assays.^[^ ^29^ ^]^ These data, together with that presented here, indicate that larger ligands with enhanced binding interactions are required for effective SARS‐CoV‐2 S‐protein RBD binding, to outcompete membrane bound ACE2 and effectively prevent viral infection.

## CONFLICT OF INTEREST

The authors declare no competing interests.

## AUTHOR CONTRIBUTION

Andrew G. Jamieson designed the study. Danielle C. Morgan, Caroline Morris, Amit Mahindra, Connor M. Blair, Gonzalo Tejeda, Imogen Herbert, Matthew L. Turnbull, Gauthier Lieber, and Nicola Logan performed the experiments. Danielle C. Morgan and Andrew G. Jamieson wrote the manuscript. Brian Smith, Brian J. Willett, Andrew B. Tobin, David Bhella, and George Baillie provided critical support and supervised the study. All authors read and approved the manuscript.

5

## Supporting information


**Appendix S1**. Supporting Information

## Data Availability

The data that supports the findings of this study are available in the supplementary material of this article including experimental procedures, neutralization, immunofluorescence, fluorescent polarization data and characterization data for all compounds, including analytical HPLC traces for final compounds.
