# Supplementary material for: Stapled ACE2 peptidomimetics designed to target the SARS‐CoV‐2 spike protein do not prevent virus internalization
Source: Pept Sci (Hoboken). 2021 Jan 8;113(4):e24217. doi: 10.1002/pep2.24217 (PMC7883042; doi:10.1002/pep2.24217)

Supporting information

Stapled ACE2 peptidomimetics designed to target the SARS-CoV-2 spike protein do not prevent virus internalisation

Danielle C. Morgan,[†#] Caroline Morris,[†#] Amit Mahindra,[†#] Connor M. Blair, Gonzalo Tejeda,[‡] Imogen Herbert,[^] Matthew L. Turnbull,[^] Gauthier Lieber,[^] Brian J. Willett,[^] Nicola Logan,[^] Brian Smith,[‡] Andrew B. Tobin,[‡] David Bhella,[˜] George Baillie and Andrew G. Jamieson[†]*.

[†]*School of Chemistry, University of Glasgow, Joseph Black Building, University Avenue, Glasgow G12 8QQ, U.K.*
^MRC-University of Glasgow Centre for Virus Research, Henry Wellcome Building, Glasgow G61 1QH, U.K.
[˜]*Sir Michael Stoker Building, Garscube Campus, 464 Bearsden Road, Glasgow G61 1QH, U.K.*
[‡]*Centre for Translational Pharmacology, Institute of Molecular Cell and Systems Biology, Davidson Building, University of Glasgow, Glasgow G12 8QQ, U.K.*
**Corresponding author: andrew.jamieson.2@glasgow.ac.uk*

	Page Number
Materials and Methods	***2-4***
Peptide Characterization Tables:	
Table S1	***5***
Table S2	***6***
Table S3	***6-7***
Table S4	***7***
Table S5	***7***
Circular Dichroism (CD):	
Equation S1 - % Helicity	***8***
Table S6 - % Helicity	***8***
Neutralisation Data - Figure S1	***9***
Immunofluorescence (IF) Data - Figure S2	***10***
Fluorescent Polarisation (FP) Data:	
Figure S3 - 30 min incubation	***11***
Figure S4 - 60 min incubation	***12***
Figure S5 - 120 min incubation	***13***
Figure S6 - 240 min incubation	***14***
Figure S7 - Overnight incubation	***15***
Figure S8 - C-terminal FITC peptides	***16***
Figure S9 - G-link Circular Dichroism (CD) Data	***16***
References	***17***
Acetylated Peptides HPLC Traces	***17-24***
FITC Peptides HPLC Traces	***24-32***
Acetylated Peptides LCMS Traces	***32-39***
FITC Peptides LCMS Traces	***40-47***

Materials and Methods

Reagents. Fmoc-protected amino acids were purchased from CEM Corporation, and Pepceuticals. N,N-Dimethylformamide (DMF) and diethyl ether (Et_2O) were purchased from Rathburn. (*R*)-N-Fmoc-*α*-(7-Octenyl)alanine, (*S*)-N-Fmoc-*α*-(4-pentenyl)alanine, triisopropylsilane (TIPS), 1,2-dichloroethane (DCE) and Grubbs 1st Catalyst were purchased from Sigma Aldrich. Trifluoroacetic acid (TFA), (DIPEA), *N,N'*-diisopropylcarbodiimide (DIC), ethyl (hydroxyamino)cyanoacetate (Oxyma Pure), fluorescein-5-isothiocyanate (FITC), Fmoc-Lys(Alloc)-OH, Fmoc-6-Ahx-OH, palladium (O)tetrakis(triphenyphosphine) ($Pd(PPh_3)_4$) and phenylsilane were purchased from Fluorochem. Morpholine was purchased from Alfa Aesar. Dichloromethane (DCM) was purchased from VWR. Acetonitrile (MeCN) was purchased from Honeywell. TentaGel S RAM resin was purchased from Rapp Polymere. All other reagents were purchased from Sigma Aldrich. S-RBD was purchased from Genscript (S-RBD, P330-F541, Product no. Z03479) and AcroBiosystems (S-RBD, R319-F541, Product no. SPD-C52H3) based on commercial availability and lead times.

Solid phase peptide synthesis (SPPS) and peptide modifications. Peptides were synthesized on a 0.1 mmol scale using either a Biotage Initiator+ Alstra (Biotage) or CEM Liberty Blue microwave assisted peptide synthesizer, with a TentaGel S RAM resin (0.24 mmol/g). Coupling of Fmoc-protected amino acids (5 eq, 0.2 M in DMF) and unnatural/orthogonally-protected amino acids (2.5 eq, 0.1 M in DMF) was achieved by treatment with DIC (5 eq, 0.5 M in DMF) and Oxyma Pure (5 eq, 0.5 M in DMF) at 90 °C for 1 min. His residues were coupled at 50 °C for 8 min. b-branched amino acids and those following unnatural amino acids were double coupled. Deprotection was achieved by treatment with morpholine (20 % in DMF with 5 % formic acid, 4 ml) at 90 °C for 1 min. The resin was washed with DMF between deprotection and coupling (4 x 4 ml), and after coupling (2 x 4 ml).

Peptides requiring *N*-terminal acetylation were treated on-resin with acetic anhydride (3 eq), DIPEA (4.5 eq) and DMF (7 ml for 0.1 mmol of resin) for 20 min with agitation. The resin was then washed with DMF (3 x 5 ml) and DCM (3 x 5 ml) prior to peptide cleave and global deprotection.

Peptides containing (*R*)-N-Fmoc-*α*-(7-Octenyl)alanine and (*S*)-N-Fmoc-*α*-(4-pentenyl)alanine were stapled by on-resin ring-closing metathesis (RCM). The resin was suspended in dry DCE before adding Grubbs 1st Catalyst (20 mol%) in DCE (4 ml for 0.1 mmol of resin), leaving for 2 h with agitation and excluding light. The resin was washed with DCE (3 ml) before repeating the RCM. The resin was then washed with DCM (2 x 5 ml) prior to peptide cleave and global deprotection.

Peptides containing Fmoc-Lys(Alloc)-OH were treated on-resin to selectively remove the Alloc protecting group. $Pd(PPh_3)_4$ (0.25 eq) and phenylsilane (25 eq) were pre-mixed in DCM (2 ml for 0.1 mmol of resin) and added to the resin, leaving for 4 h with agitation. The resin was then washed with DMF (2 x 5 ml) and DCM (2 x 5 ml) prior to peptide cleave and global deprotection.

Peptides requiring an *N*-terminal fluorescent label were treated on-resin with FITC (2 eq), DIPEA (8 eq) and DMF (4 ml for 0.1 mmol of resin) following coupling of Fmoc-6-Ahx-OH as a spacer. The resin was then washed with DMF (2 x 5 ml) and DCM (2 x 5 ml) prior to peptide cleave and global deprotection.

Peptides requiring a *C*-terminal fluorescent label were treated on-resin with FITC (2 eq), DIPEA (8 eq) and DMF (4 ml for 0.1 mmol of resin) following alloc deprotection of an orthogonally-protected Lys. The resin was then washed with DMF (2 x 5 ml) and DCM (2 x 5 ml) prior to peptide cleave and global deprotection.

Peptide cleavage and global deprotection. Peptides were cleaved from the resin using a cocktail of TFA (95 %), TIPS (2.5 %) and H_2O (2.5 %) for 1 h with agitation. The resin was subsequently filtered and the TFA evaporated using a stream of N_2, the peptide with precipitated with cold Et_2O and centrifuged (4500 rpm for 5 min). Peptides were dissolved in a mixture of H_2O and MeCN with 0.1 % TFA and lyophilized on a Christ Alpha 2-4 LO plus freeze dryer.

Peptide purification. Crude peptides were purified by reverse-phase high-performance liquid chromatography (RP-HPLC) using either an Agilent Technologies 1260 Infinity RP-HPLC system or a Dionex RP-HPLC system with Dionex P680 pumps and a Dionex UVD170U UV-vis detector, each with a Phenomenex Gemini column (5 mm C18, 250 x 21.2 mm). Purified peptides were analysed on a Shimadzu RP-HPLC system with Shimadzu LC-20AT pumps, a Shimadzu SIL20A autosampler and a Shimadzu SPD-20A UV-vis detector using a Phenomenex Aeris column (5 mm C18, 100 Å, 150 x 10 mm). Peptides were eluted with linear gradients at column-dependent flow rates (1 ml/min for the Aeris, 10 ml/min for the Gemini), where buffer A = 0.1 % TFA in H_2O and buffer B = 0.1 % TFA in MeCN. Liquid chromatography mass spectrometry (LCMS) was performed on a Thermo Scientific LCQ Fleet Ion Trap Mass Spectrometer using positive mode electrospray ionisation (ESI^+). Where buffer A = 0.1 % TFA in H_2O and buffer B = 0.1 % TFA in MeCN, a linear gradient of 0-100 % B over 20 min with a flow rate of 1 ml/min was used with a Reprosil-Gold column (3 mm C18, 150 x 4 mm).

Circular dichroism (CD) spectroscopy. CD spectra were obtained at room temperature using a JASCO J-810 CD spectrometer. A range of 190 – 260 nm was scanned at a speed of 50 nm/min, with a 1 nm data pitch, a 1 nm bandwidth and an 8 s response time. Samples were prepared (100 µM) in phosphate buffered saline (PBS; pH 7.4), and CD spectra measured in a 1 mm quartz cuvette. Raw data (mdeg) were converted to mean residue ellipticity (MRE; deg cm^2 $dmol^{-1}$ res^{-1}) by normalizing for path length, peptide concentration, and number of amide bonds.

Neutralisation Assay

HEK 293 cells expressing Human ACE2

ΔSfiI-ΔRFP-SCRPSY-ACE2 lentivirus vector design. pΔSfiI-ΔRFP-SCRPSY is a derivative of the lentiviral vector SCRPSY (GenBank accession number KT368137.1) (Kane *et al.*, 2016). The SfiI site at position 7582 was ablated using overlap extension PCR (7583CGG7585 to 7583AT-7585). To remove TagRFP expression, the 5'-NheI-SphI-3' fragment containing the TagRFP ORF was replaced with the puromycin-N-acetyltransferase selection gene ORF flanked by corresponding 5'NheI and 3'SphI sites (chemically synthesised in pUC57-AmpR by GeneWiz). To make pΔSfiI-ΔRFP-SCRPSY-ACE2, the sequence of Homo sapiens angiotensin I converting enzyme II (ACE2) ORF (GenBank NM_001371415.1) with flanking SfiI sites (chemically synthesised in pEX-A258 (Eurofins) was sub-cloned into pΔSfiI-ΔRFP-SCRPSY. The ACE2 ORF sequence was confirmed using Sanger sequencing (Eurofins).

Transduction of 293 or A549 cells for stable ACE2 overexpression. 293T cells were transiently transfected with 5 µg of pΔSfiI-ΔRFP-SCRPSY-ACE2 or the empty vector pΔSfiI-ΔRFP-SCRPSY as a control, 5 µg of GagPol expression plasmid pNLGP, and 1 µg of the VSV glycoprotein expression plasmid pVSV-G, which have been described previously (Rihn *et al.*, 2019). Lentiviral vector-containing supernatant was passed through a 0.2 µm pore size filter prior to transduction of 293 or A549 cells (van Diepen *et al.*, 2010; Daniloski *et al.*, 2020). Transduced cells were selected for using puromycin dihydrochloride (2 µg/µl) (Melford).

Cell maintenance. HEK 293T cells and HEK 293 cells stably expressing human ACE2 were maintained in Dulbecco's Modified Eagle Medium (DMEM) supplemented with 10% foetal bovine serum, 100 IU/ml penicillin, 100 lg/ml streptomycin, 2 mM glutamine and 0.11 mg/ml sodium pyruvate. 293T cells that stably express human ACE2 was further supplemented puromycin at 2 ug/ml. All media and supplements were obtained from Life Technologies Ltd., Paisley, UK.

Pseudotype virus preparation. 293T cells were cultured in DMEM supplemented with 10% foetal bovine serum at 37°C, 5% CO_2. Cells were seeded (2x10^6) in 10cm dishes (Corning) 24 hours prior to transfection. Cells were transiently co-transfected with 1ug HIV pNL4-3-Luc-E$^-$R$^-$luc, *Env*-deleted pro virus containing the luciferase reported gene (Connor et al., 1995) and 0.9ug pCAGGS SARS-CoV-2 Spike (Wuhan-Hu-1 strain, NIBSC) using polyethyleneimine (Sigma-Aldrich). Cells were incubated at 37°C, 5% CO_2 for 72 hours after which the pseudotype virus was harvested and titrated on human ACE2 293T cells. Luciferase activity was measured after 48 hours by adding Steadylite plus™ (Perkin Elmer) substrate to the wells and reading luminescence on a EnSight Multimode Plate Reader machine (PerkinElmer).

Pseudotype-based inhibition assay. Three-fold dilutions of the peptides were prepared in complete DMEM starting from a 5uM concentration. Human sACE2 was prepared using three-fold dilutions starting from 1uM. DMSO negative controls were prepared using the same volumes as the tested peptides. 25ul of the diluted samples was added to white opaque 96 well tissue culture plate (Culturplate-96, Perkin Elmer, Coventry, UK) 25ul of the SARS CoV-2 S-protein expressing pseudotype virus was then added. Plates were incubated at 37°C, 5% CO_2 for 1 hour. 2x10^4 293T ACE2 expressing cells were added to each well. Plates were incubated at 37°C, 5% CO_2 for 48 hours. Luciferase activity was measured as described above. Inhibition was calculated as a percentage against DMSO negative controls.

Immunofluoresence Assay

Culture and treatment of A549 cells. A549 lung cancer cells were grown in high-glucose Dulbecco's Modified Eagle's Medium (DMEM) containing 10% foetal bovine serum (FBS), 2 mm L-glutamine, 100 U/ml penicillin and 100 µg/ml streptomycin (1% P/S) and 2 µg/ml puromycin to select cells transduced with the lentiviruses SCRPSY-ACE2 or the empty control.
Stapled peptides (2 or 10 uM final concentration) or soluble ACE2 Fc Chimera (hACE2, Genscript, Z03484) were incubated with 50 or 100 nM His-tagged SARS-CoV-2 Spike protein RBD (Genscript for the 1st generation peptides, AcroBiosystems for the 2nd generation peptides) for 30 min in culture media at 37° C. Afterwards, this solution was added to A549 cells and further incubated for 3 h.

Western blot analysis. Detergent-soluble proteins were isolated from cells by disruption in RIPA buffer (25 mM Tris–HCl, 150 mM NaCl, 0.5% sodium deoxycholate, 1% NP-40, 1 mM DTT, 0.1% SDS; pH 7.5), supplemented with protease and phosphatase inhibitors (Roche). Protein samples were boiled in SDS loading buffer (10% SDS, 300mM Tris-HCl, 0.05% bromothymol blue, 10% β-mercaptoethanol) and equal amounts of cell lysates were resolved on 10% SDS-polyacrylamide gels and transferred to nitrocellulose membranes (GE Healthcare). After Ponceau-S staining (0.2% Ponceau-S red, 1% acetic acid) and blocking in 5% non-fat dry milk in TBST (25 mM Tris-HCl; pH 7.6, 100 mM NaCl, 0.5% Tween-20), membranes were incubated with mouse anti-Histidine (Sigma-Aldrich, H1029), rabbit anti-ACE2 (Abcam, ab15348) or mouse anti-α tubulin (Sigma-Aldrich, T9026) primary antibodies overnight at 4 °C. After washing with TBST, the appropriate IRDye secondary antibody (925-68072 or 925-32213, Li-Cor Biosciences) was added to the membranes for 1 h. Finally, immunoreactive bands

were visualized using the Licor Odyssey system and densitometry of fluorescence was measured with Image Studio Lite (Licor Biosciences).

Immunocytochemistry and image analysis. Cells grown on coverslips were fixed with 4% paraformaldehyde for 1 h at room temperature and then washed with phosphate-buffered saline (PBS). Coverslips were blocked and permeabilized with PBS supplemented with 1% BSA and 0.2% Triton X-100 for 1 h. Afterwards, cells were incubated overnight at 4°C in a humidity chamber with primary antibodies diluted in the same solution recognizing Histidine (Sigma-Aldrich, H1029) and ACE2 (Abcam, ab15348). Detection was achieved using secondary antibodies conjugated to Alexa Fluor 488 or Alexa Fluor 594 (Life Technologies) and coverslips were mounted on glass slides using ProLong Gold with DAPI (P36935, ThermoFisher Scientific). All confocal images are single sections acquired with an inverted Zeiss Pascal laser-scanning confocal microscope (LSM) 880 with an oil immersion objective using the ZEN image acquisition software. Co-localization analysis was performed by calculating Pearson's Correlation coefficient (PCC) in a minimum of 5 images per condition using the Coloc 2 plugin for ImageJ Fiji (NIH Image).

Fluorescence Polarisation Assay

Fluorescent polarisation (FP) assay was used to assess direct binding kinetics between ACE2 peptides and SARS-CoV-2 S-RBD – polyhistidine tag protein (AcroBiosystems) (Moerke, N.J. 2009). Direct binding kinetics were assessed under the following conditions: black, flat bottom, non-stick 384 well plates (corning) contained 100nM of respective N-FITC tag ACE2 peptide (diluted in PBS, 0.01% Tween-20, 1mM DTT, pH 8.0) and an increasing concentration of purified S-RBD2 protein prepared in serial dilution (1:2 dilutions) in PBS buffer. FP measurements were taken following 30, 60, 120 and 240-minutes post-incubation. Non-specific binding was assessed using a negative control N-FITC tag ACE2 scrambled peptide. Fluorescence polarisation (mP) was measured at an excitation/emission wavelength of 485nm/535nm using a Mithras LB 940 plate reader (Berthold Technologies) and data analysed in GraphPad Prism 6.0.

Name	Sequence	Mass* (Da)		Purity %
		Calculated	Observed $[M+H]^+$	
**Native 1** (Ac)	Ac–IEEQAKTFLDKFNHEAEDLFYQS–NH_2	2842.32	2843.01	96
**2** (Ac, S)	Ac–IR_8EQAKTFS_5LKFNHEAEDLFYQS–NH_2	2892.43	2891.49	95
**3** (Ac, S)	Ac–IEEQR_8KTFLDKS_5NHEAEDLFYQS–NH_2	2916.45	2917.26	95
**4** (Ac, S)	Ac–IEEQAR_8TFLDKFS_5HEAEDLFYQS–NH_2	2892.42	2892.99	90
**5** (Ac, S)	Ac–IEEQAKTFR_8DKFNHES_5EDLFYQS–NH_2	2950.44	2951.25	98
**6** (Ac, S)	Ac–IEEQAKTFLDKR_8NHEAEDS_5FYQS–NH_2	2874.41	2874.99	98
**7** (Ac, S)	Ac–IEEQAKTFLDKFR_8HEAEDLS_5YQS–NH_2	2873.44	2874	91
**8** (Ac, S)	Ac–IEEQAKTFLDKFNHER_8EDLFYQS_5–NH_2	2976.49	2977.5	98
**9** (Ac)	Ac–HEAEDLFYQS–NH_2	1278.55	1278.5	100
**10** (Ac, S)	Ac–HES_5EDLS_5YQS–NH_2	1310.61	1310.66	96
**11** (Ac)	Ac–IEEQAKTFLDKFNHE–NH_2	1888.93	1889.34	100
**12** (Ac, S)	Ac–IEEQR_8KTFLDKS_5NHE–NH_2	1963.04	1963.5	100
**13** (Ac)	Ac–TFLDKFNHEAEDL–NH_2	1618.76	1619	100
**14** (Ac, S)	Ac–TFR_8DKFNHES_5EDL–NH_2	1726.86	1727.34	96
**15** (Ac, S)	Ac–TS_5LDKS_5NHEAEDL–NH_2	1574.79	1575.16	96
**Scrambled negative control** (Ac)	Ac–FHTSEYDEQNEIEAAQLFKDFLK–NH_2	2842.32	2843.25	98
**Stapled negative control** (Ac, S)	Ac–IEEQAKR_8FLDKFNS_5EAEDLFYQS–NH_2	2898.47	2897.01	99

Table S1: *Name, sequence and mass of peptides with N-terminal acetylation. Abbreviations: Ac = acetylated, NH_2 = C-terminal amide, S = stapled, R_8 = (R)-N-Fmoc-α-(7-Octenyl)alanine, S_5 = (S)-N-Fmoc-α-(4-pentenyl)alanine. *Calculated and observed masses for peptides containing unnatural amino acids after stapling.*

Name	Sequence	Mass* (Da)		Purity (%)
		Calculated	Observed $[M+H]^+$	
**G-link** (Ac)	Ac-IEEQAKTFLDKFNHEAEDLFYQSS-G-LGKGDFR-NH_2	3760.74	3759.82	99
**D30E** (Ac)	Ac-IEEQAKTFLEKFNHEAEDLFYQSS-G-LGKGDFR-NH_2	3774.84	3773.84	95
**G-link** (Ac, S)	Ac-IR_8EQAKTFS_5DKFNHEAEDLFYQSS-G-LGKGDFR-NH_2	3894.99	3893.96	99
**Mutant** (Ac)	Ac-IEEQAKYFLEWFNPEAEDLFYLSS-G-FGKGDFR-NH_2	3873.84	3873.75	98

Table S2: *Name, sequence and mass of G-link and mutant peptides. Abbreviations: Ac = acetylated, NH_2 = C-terminal am*ide.

Name	Sequence	Mass* (Da)		Purity (%)
		Calculated	Observed $[M+H]^+$	
**Native 1** (FITC)	FITC-X-IEEQAKTFLDKFNHEAEDLFYQS-NH_2	3302.45	3303.51	99
**2** (FITC, S)	FITC-X-IR_8EQAKTFS_5LKFNHEAEDLFYQS-NH_2	3352.54	3353.49	96
**3** (FITC, S)	FITC-X-IEEQR_8KTFLDKS_5NHEAEDLFYQS-NH_2	3376.56	3377.76	99
**4** (FITC, S)	FITC-X-IEEQAR_8TFLDKFS_5HEAEDLFYQS-NH_2	3352.53	3353.49	97
**5** (FITC, S)	FITC-X-IEEQAKTFR_8DKFNHES_5EDLFYQS-NH_2	3410.55	3411.75	99
**6** (FITC, S)	FITC-X-IEEQAKTFLDKR_8NHEAEDS_5FYQS-NH_2	3334.52	3335.49	99
**7** (FITC, S)	FITC-X-IEEQAKTFLDKFR_8HEAEDLS_5YQS-NH_2	3333.56	3334.50	98
**8** (FITC, S)	FITC-X-IEEQAKTFLDKFNHER_8EDLFYQS_5-NH_2	3436.60	3437.76	99
**9** (FITC)	FITC-X-HEAEDLFYQS-NH_2	1738.66	1738.84	95
**10** (FITC, S)	FITC-X-HES_5EDLS_5YQS-NH_2	1770.72	1771.00	99
**11** (FITC)	FITC-X-IEEQAKTFLDKFNHE-NH_2	2349.04	2349.75	99
**12** (FITC, S)	FITC-X-IEEQR_8KTFLDKS_5NHE-NH_2	2423.15	2424.00	97
**13** (FITC)	FITC-X-TFLDKFNHEAEDL-NH_2	2078.87	2079.51	99
**14** (FITC, S)	FITC-X-TFR_8DKFNHES_5EDL-NH_2	2186.96	2187.75	99
**15** (FITC, S)	FITC-X-TS_5LDKS_5NHEAEDL-NH_2	2034.90	2035.34	99

**Scrambled negative control** (FITC)	FITC-X-FHTSEYDEQNEIEAAQLFKDFLK-NH$_2$	3302.45	3303.51	98
**Stapled negative control** (FITC, S)	FITC-X-IEEQAKR$_8$FLDKFNS$_5$EAEDLFYQS-NH$_2$	3358.58	3357.51	99

Table S3: *Name, sequence and mass of peptides with N-terminal fluorescent labelling. Abbreviations: FITC = fluorescein-5-isothiocyanate, X = Fmoc-6-Ahx-OH, NH$_2$ = C-terminal amide, S = stapled, R$_8$ = (R)-N-Fmoc-α-(7-Octenyl)alanine, S$_5$ = (S)-N-Fmoc-α-(4-pentenyl)alanine. *Calculated and observed masses for peptides containing unnatural amino acids after stapling.*

Name	Sequence	Mass* (Da)		Purity (%)
		Calculated	Observed [M+H]$^+$	
**G-link** (FITC)	FITC-X-IEEQAKTFLDKFNHEAEDLFYQSS-G-LGKGDFR-NH$_2$	4221.00	4219.43	99
**D30E** (FITC)	FITC-X-IEEQAKTFLEKFNHEAEDLFYQSS-G-LGKGDFR-NH$_2$	4233.94	4235.68	98
**G-link** (FITC, S)	FITC-X-IR$_8$EQAKTFS$_5$DKFNHEAEDLFYQSS-G-LGKGDFR-NH$_2$	4356.00	4354.03	99

Table S4: *Name, sequence and mass of peptides with N-terminal fluorescent labelling. Abbreviations: FITC = fluorescein-5-isothiocyanate, X = Fmoc-6-Ahx-OH, NH$_2$ = C-terminal amide, S = stapled, R$_8$ = (R)-N-Fmoc-α-(7-Octenyl)alanine, S$_5$ = (S)-N-Fmoc-α-(4-pentenyl)alanine. *Calculated and observed masses for peptides containing unnatural amino acids after stapling.*

Name	Sequence	Mass* (Da)		Purity (%)
		Calculated	Observed [M+H]$^+$	
**Native 1** (*C*-terminal FITC)	Ac-IEEQAKTFLDKFNHEAEDLFYQSK(FITC)-NH$_2$	3359.48	3360.75	97
**Scrambled** (*C*-terminal FITC)	Ac-FHTSEYDEQNEIEAAQLFKDFLKK(FITC)-NH$_2$	3359.48	3360.51	97

Table S5: *Name, sequence and mass of peptides with C-terminal fluorescent labelling. Abbreviations: FITC = fluorescein-5-isothiocyanate.*

Circular Dichroism (CD):

Equation S1: % Helicity Equation

$$(\mathbf{1})\ \%\, Helicity = \left(\frac{\theta_{222} - \theta_c}{\theta_{222\infty} - \theta_c}\right) \times\ 100$$

$$(\mathbf{2})\ \theta_c = 2220 - 53T$$

$$(\mathbf{3})\ \theta_{222\infty} = (-44000 + (250\ \times T)) \times \left(1 - \frac{k}{Np}\right)$$

Peptide	% Helicity
1	9.0
2	35.4
3	55.6
4	57.9
5	31.3
6	23.5
7	54.5
8	72.2
9	5.9
10	33.4
11	9.6
12	12.2
13	8.6
14	16.1
15	21.0

Table S6: *% helicities of peptides 1 – 15 using the MRE value at 222 nm and Equation S1.*

Neutralisation Data:

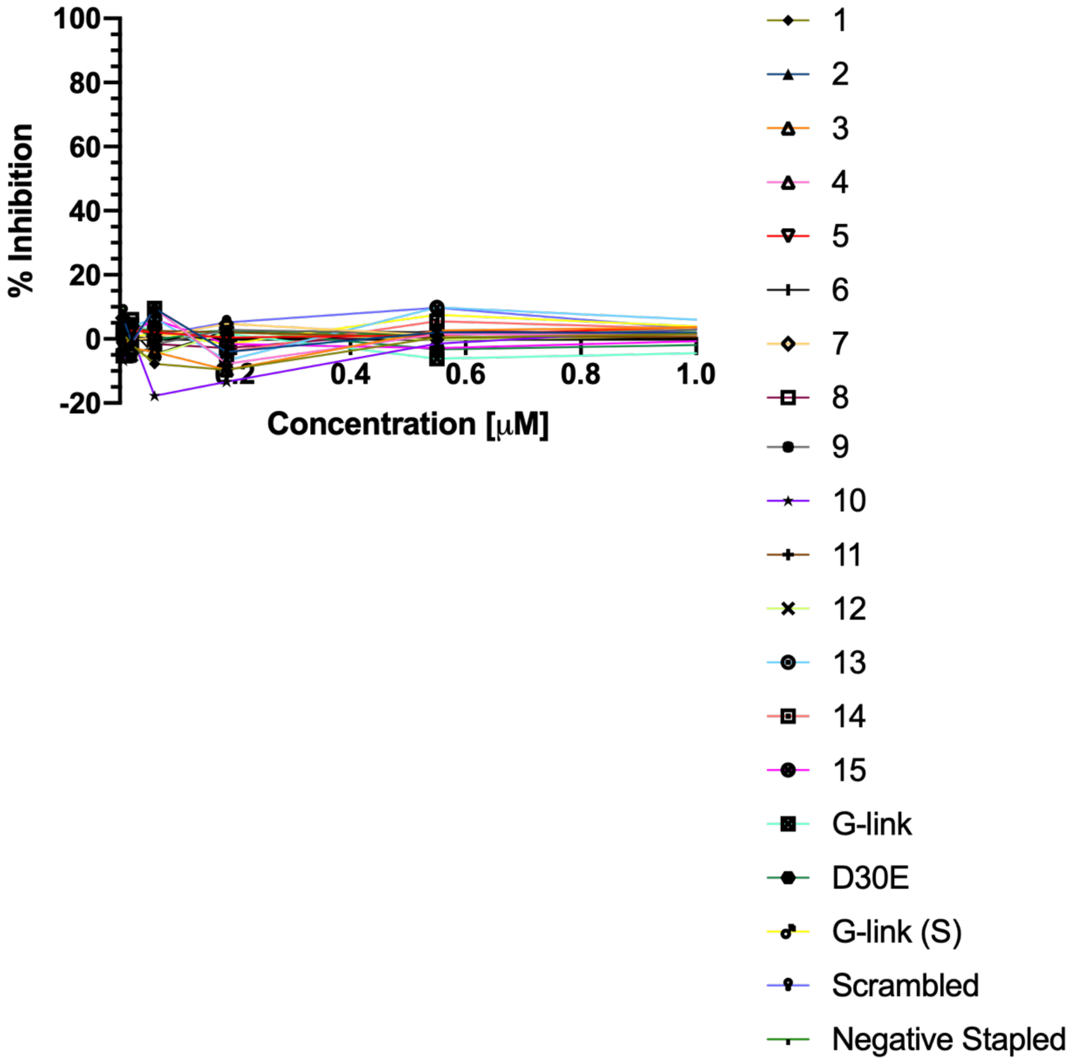

Figure S1: *Neutralisaiton data for all of the peptides.*

Immunofluorescence (IF) Data:

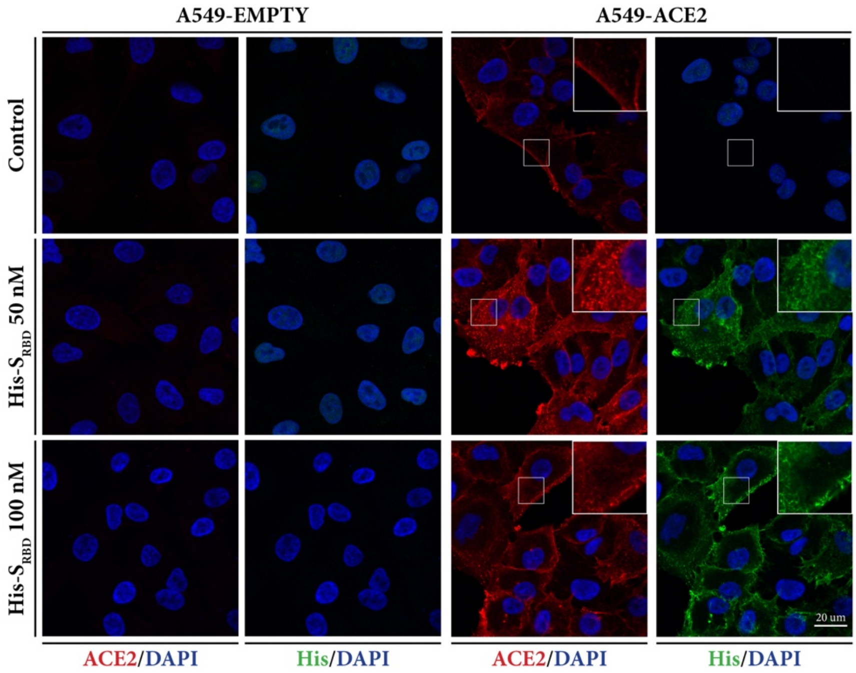

Figure S2: *The incubation of a commercial His-tagged S-RBD protein sourced from Genscript (P330-F541) stimulates the internalisation of ACE2 overexpressed in the lung cancerogenic cell line A549. No S-RBD signal was observed in control A549 cells, indicating that the internalisation is ACE2-dependent. 50 or 100 nM S_{RBD} (Genscript) for 3 h. Scale bar, 20 µM.*

Fluorescence Polarisation (FP) Data:

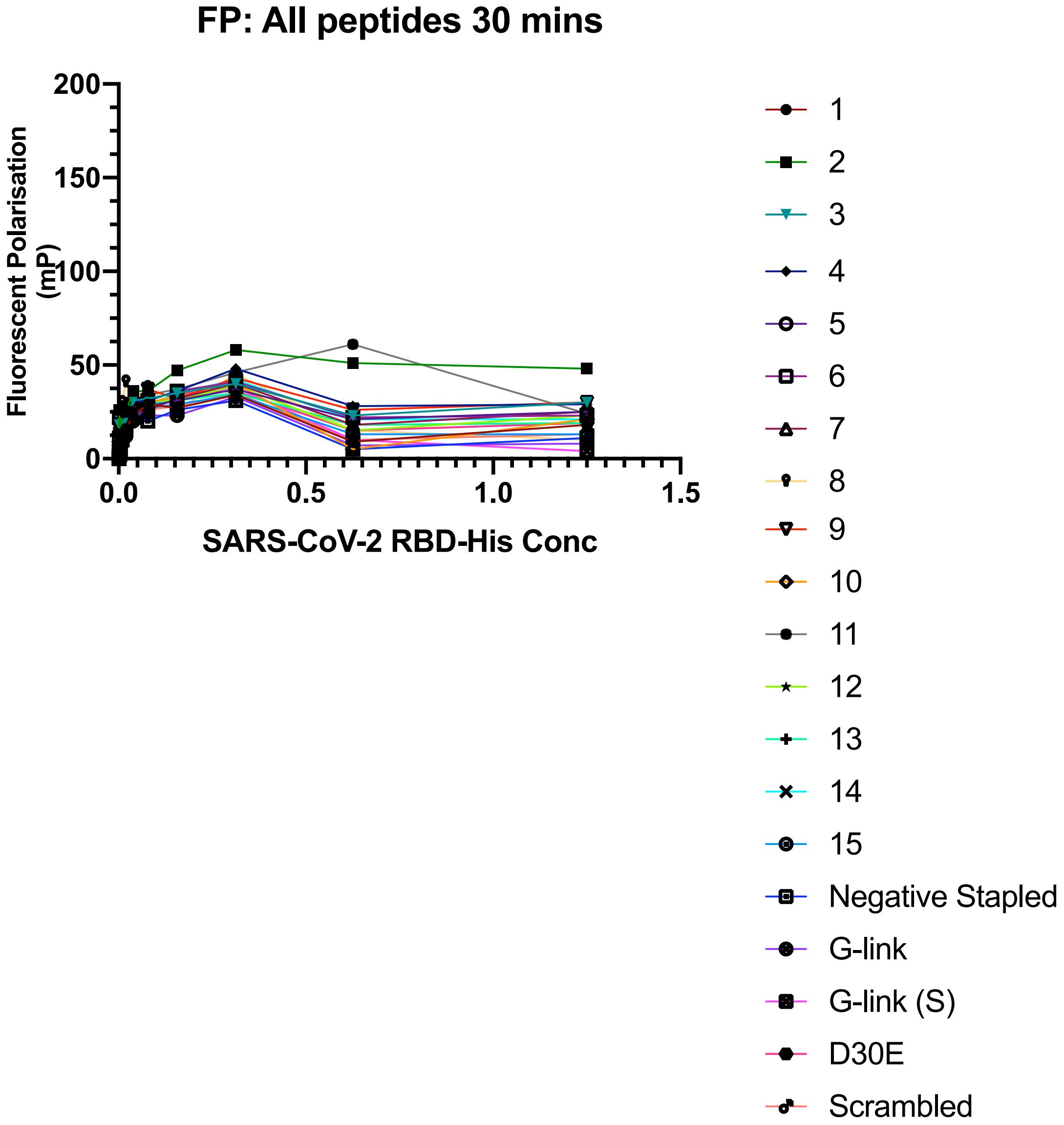

Figure S3: *Fluorescent polarisation measurements for all of the peptides taken at 30 minutes post-incubation at room temperature.*

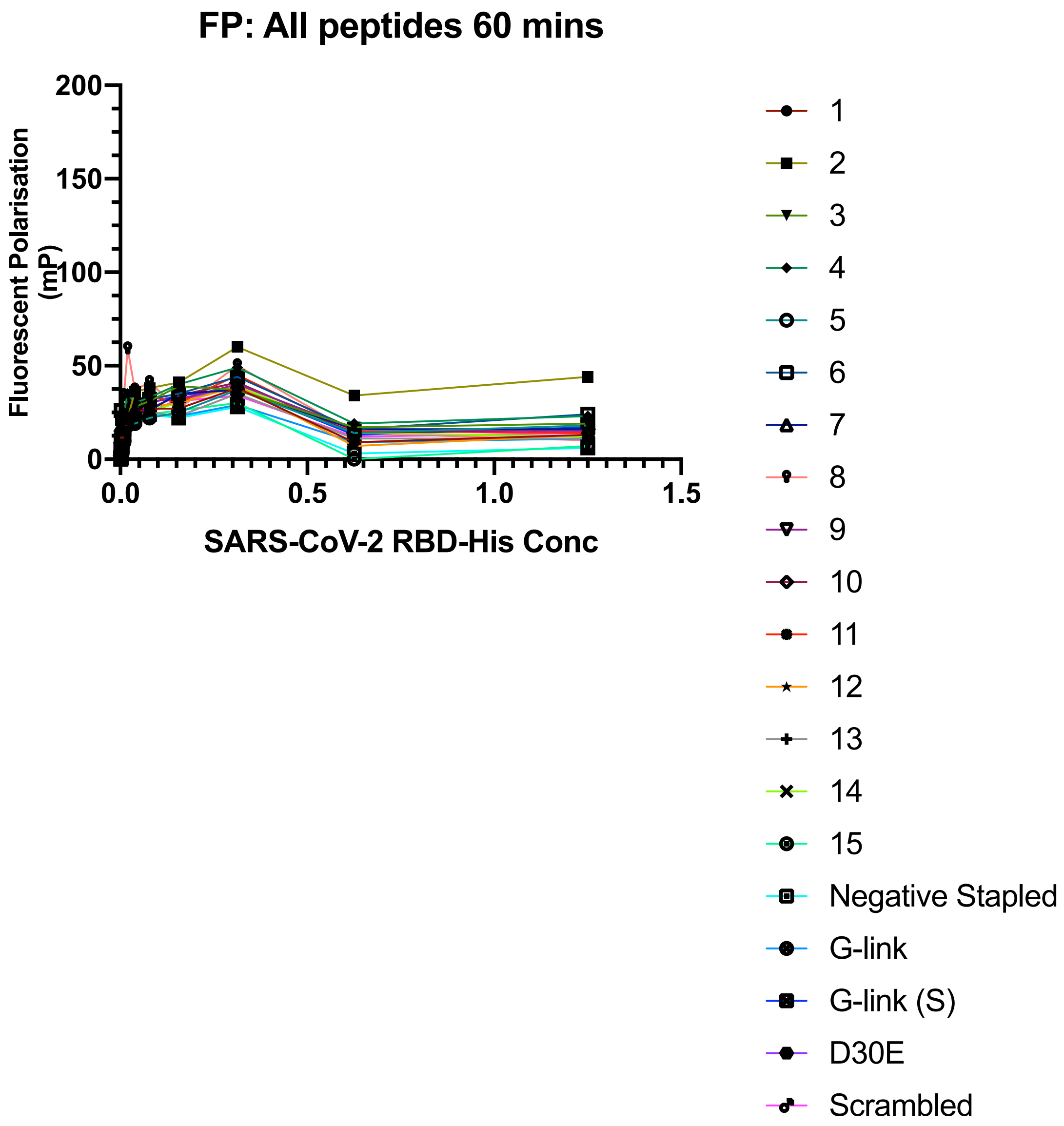

Figure S4: *Fluorescent polarisation measurements for all of the peptides taken at 60 minutes post-incubation at room temperature.*

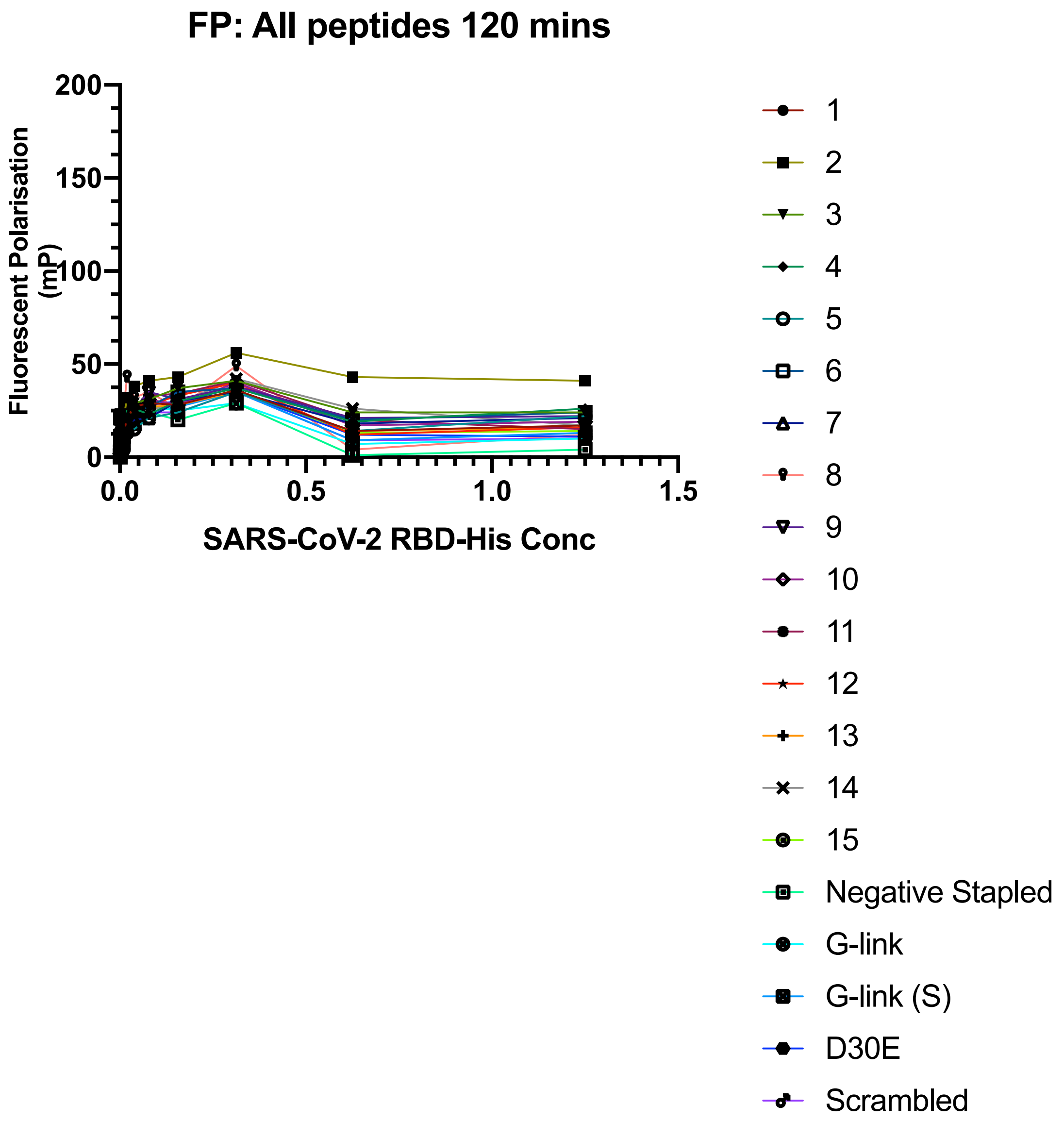

Figure S5: *Fluorescent polarisation measurements for all of the peptides taken at 120 minutes post-incubation at room temperature.*

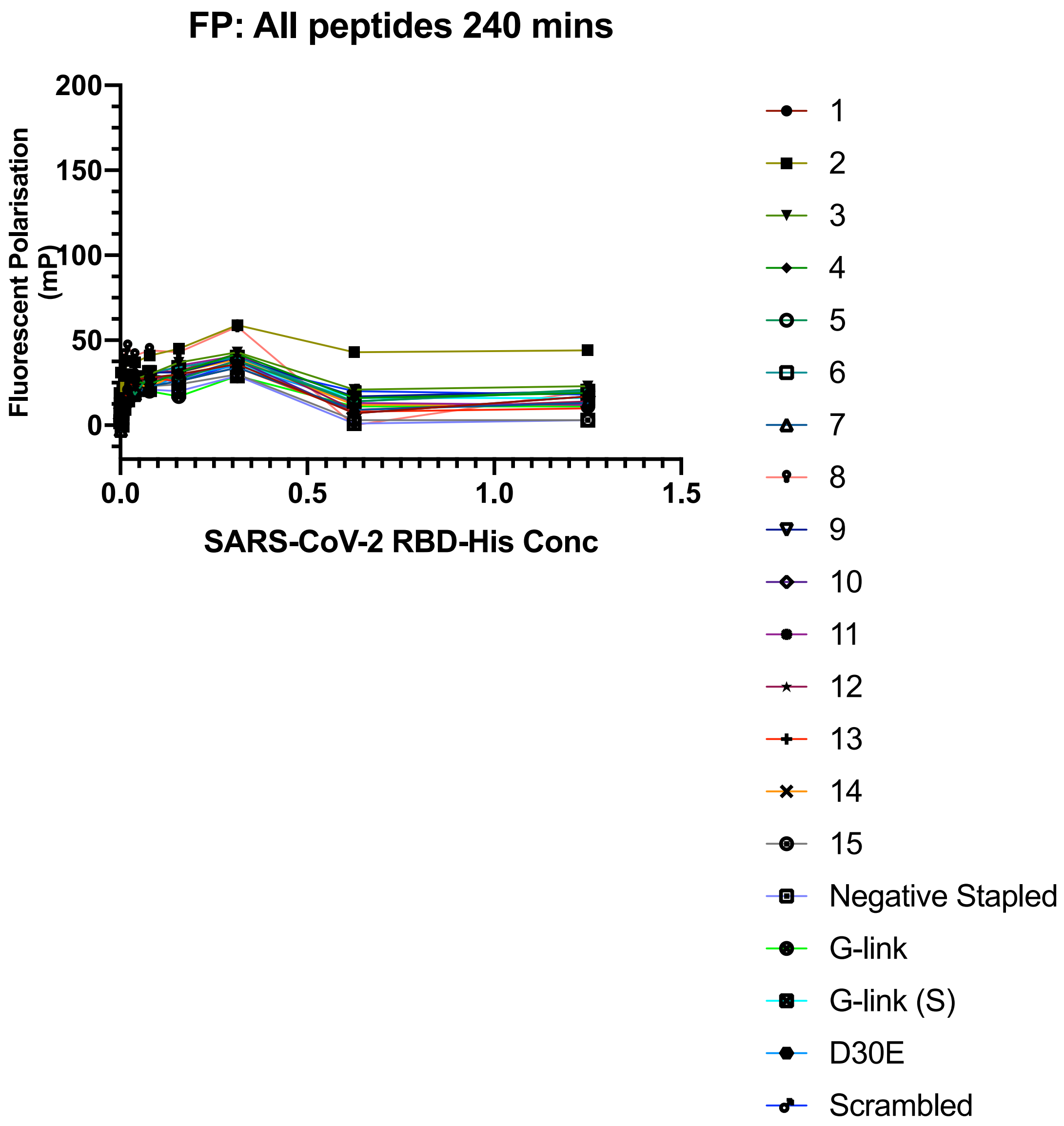

Figure S6: *Fluorescent polarisation measurements for all of the peptides taken at 240 minutes post-incubation at room temperature.*

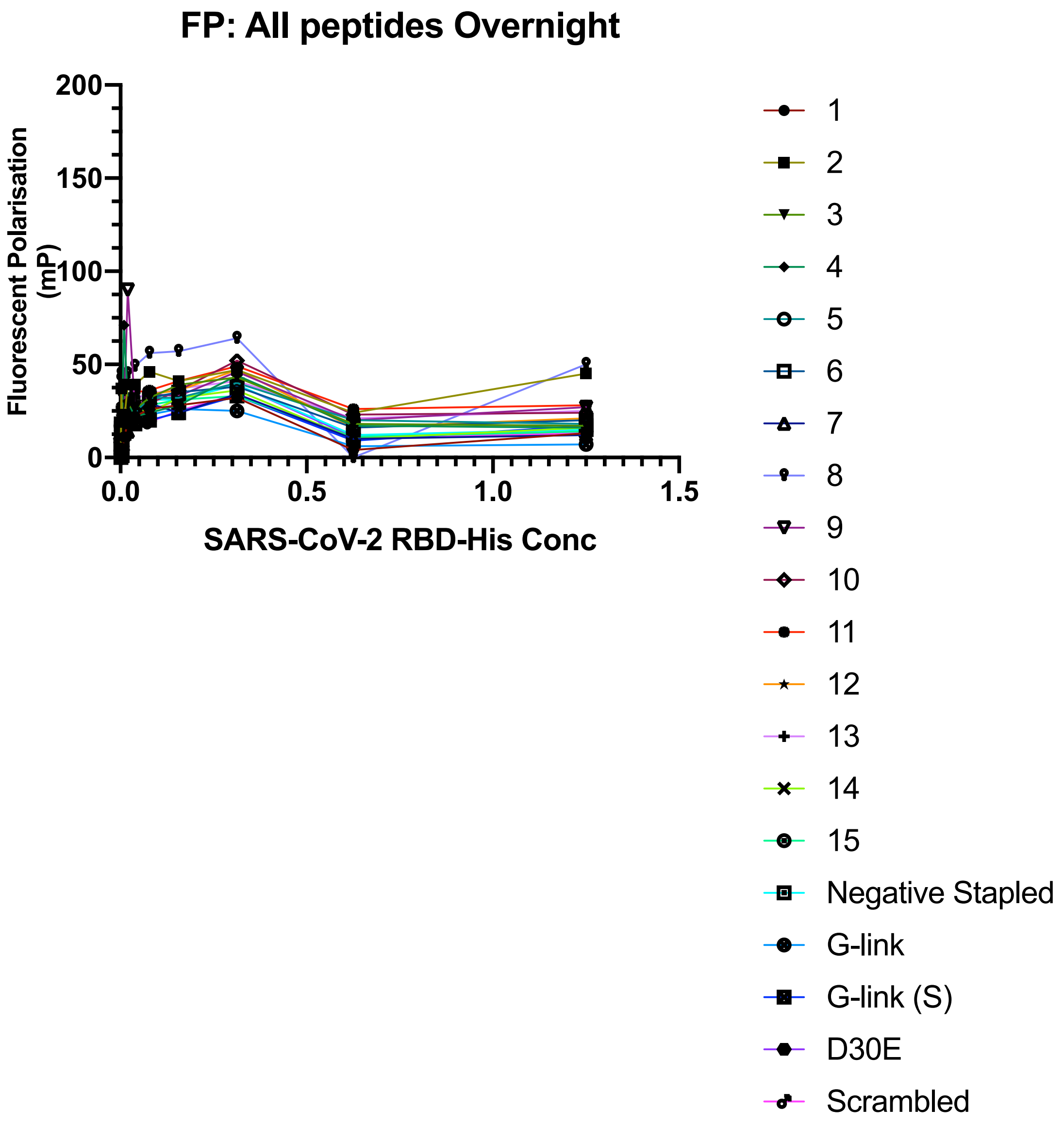

Figure S7: *Fluorescent polarisation measurements for all of the peptides taken overnight post-incubation at room temperature.*

C-terminal FITC Peptides - Peptide 1 vs. Scrambled

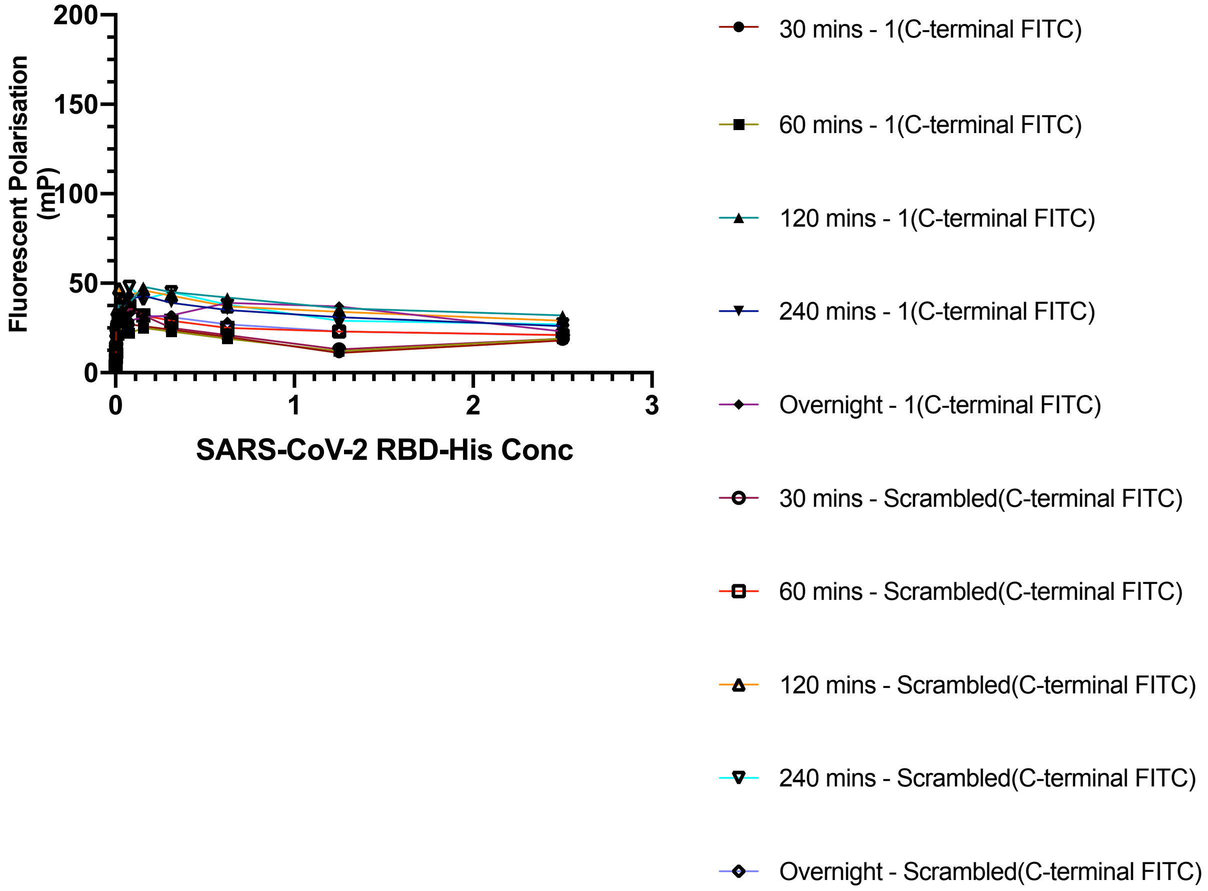

Figure S8: *Fluorescent polarisation measurements for the C-terminally FITC labelled peptide 1 and Scrambled peptide taken at 30 mins, 60 mins, 120 mins, 240 mins and overnight post-incubation at room temperature.*

G-link Peptides Circular Dichroism (CD):

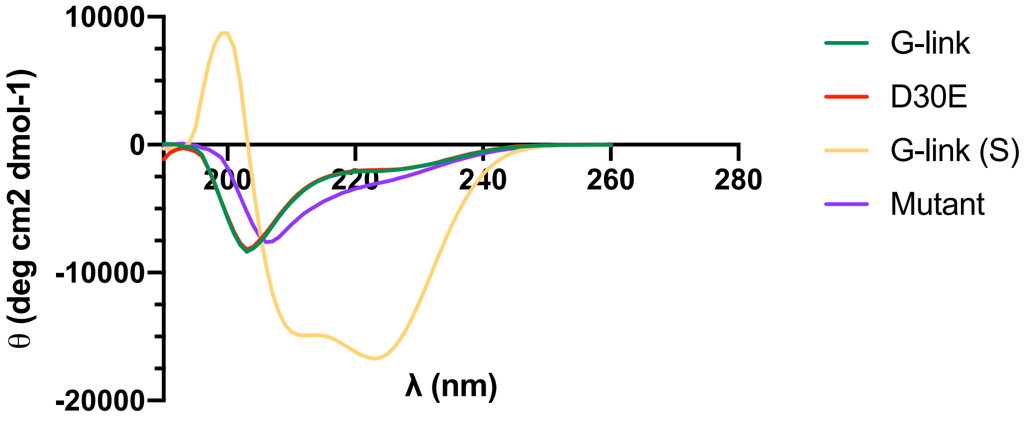

Figure S9: *Circular dichroism spectra of the G-link and Mutant peptidomimetics. Conditions: peptides 100 µM in PBS, pH 7.4. Spectra recorded between 190 – 260 nm.*

References:

Daniloski, Z., Jordan, T.X., Wessels, H.H., Hoagland, D.A., Kasela, S., Legut, M., Maniatis, S., Mimitou, E.P., Lu, L., Geller, E., Danziger, O., Rosenberg, B.R., Phatnani, H., Smibert, P., Lappalainen, T., tenOever, B.R., Sanjana, N.E., (2020) Identification of Required Host Factors for SARS-CoV-2 Infection in Human Cells. *Cell*, 24, 31394-31395.

Kane, M., Zang, T.M., Rihn, S.J., Zhang, F., Kueck, T., Alim, M., Schoggins, J., Rice, C.M., Wilson, S.J., and Bieniasz, P.D. (2016). Identification of Interferon-Stimulated Genes with Antiretroviral Activity. *Cell host & microbe,* 20, 392-405.

Rihn, S.J., Aziz, M.A., Stewart, D.G., Hughes, J., Turnbull, M.L., Varela, M., Sugrue, E., Herd, C.S., Stanifer, M., Sinkins, S.P., et al. (2019). TRIM69 Inhibits Vesicular Stomatitis Indiana Virus., *J. Virol.*, 93, e00951-00919.

Moerke, N.J. (2009). Fluorescence Polarization (FP) Assays for Monitoring Peptide-Protein or Nucleic Acid-Protein Binding. *Curr. Protoc. Chem. Biol.*, 1: 1-15.

van Diepen, A., Brand, H. K., Sama, I., Lambooy, L.H., van den Heuvel, L.P., van der Well. L., Huynen, M., Osterhaus, A.D., Andeweg, A.C., Hermans, P.W., (2010) Quantitative proteome profiling of respiratory virus-infected lung epithelial cells. *J Proteomics*. 5; 1680-1693.

Acetylated Peptides HPLC Traces:

1(Ac)_pure_50min

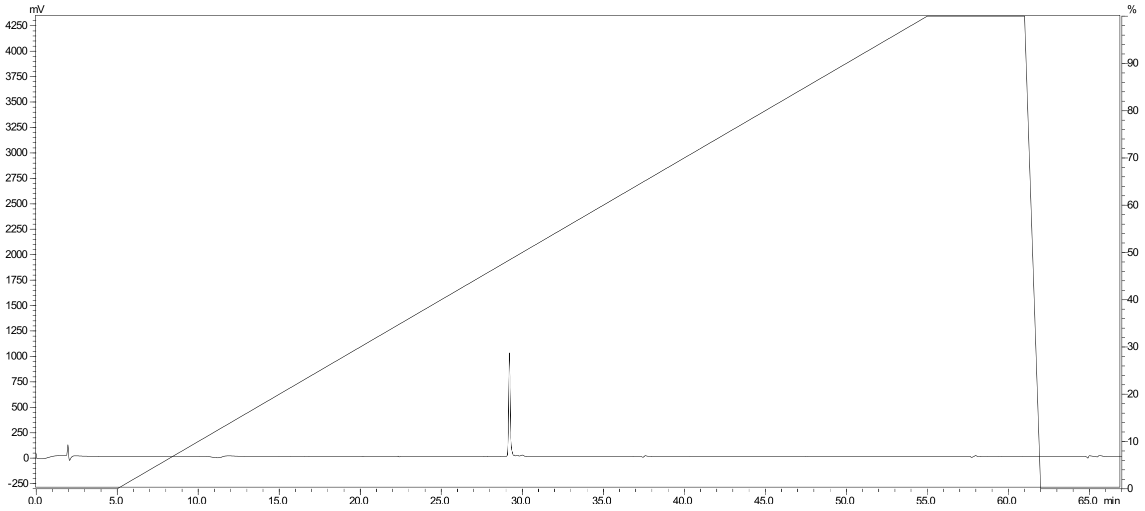

2(Ac)_pure_50min

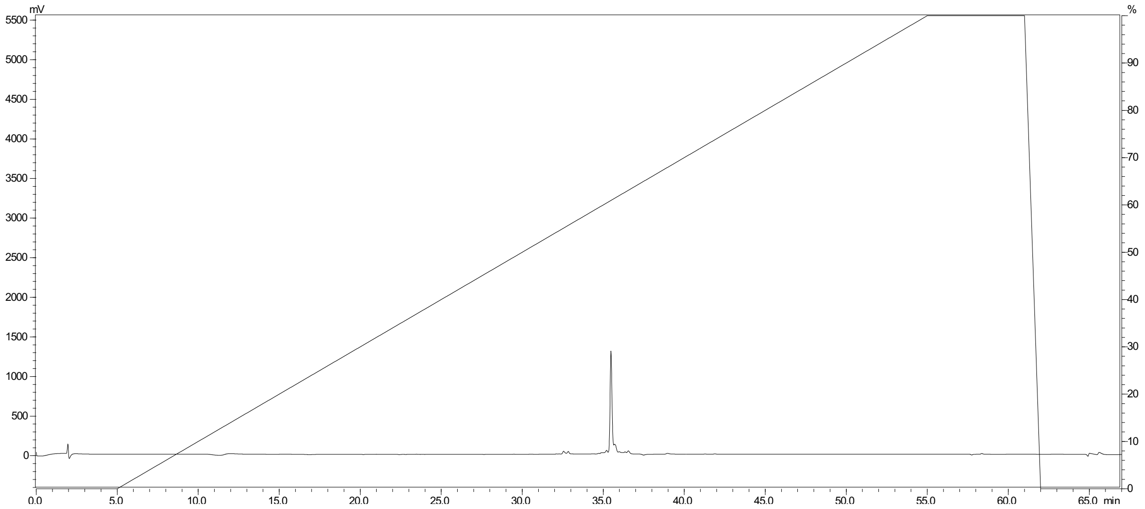

3(Ac)_pure_50min

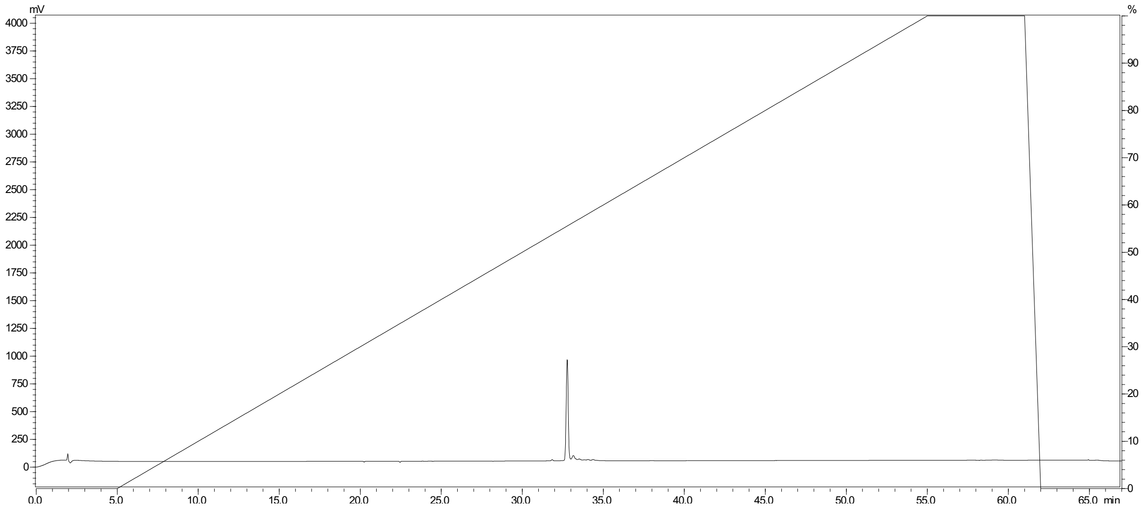

4(Ac)_pure_50min

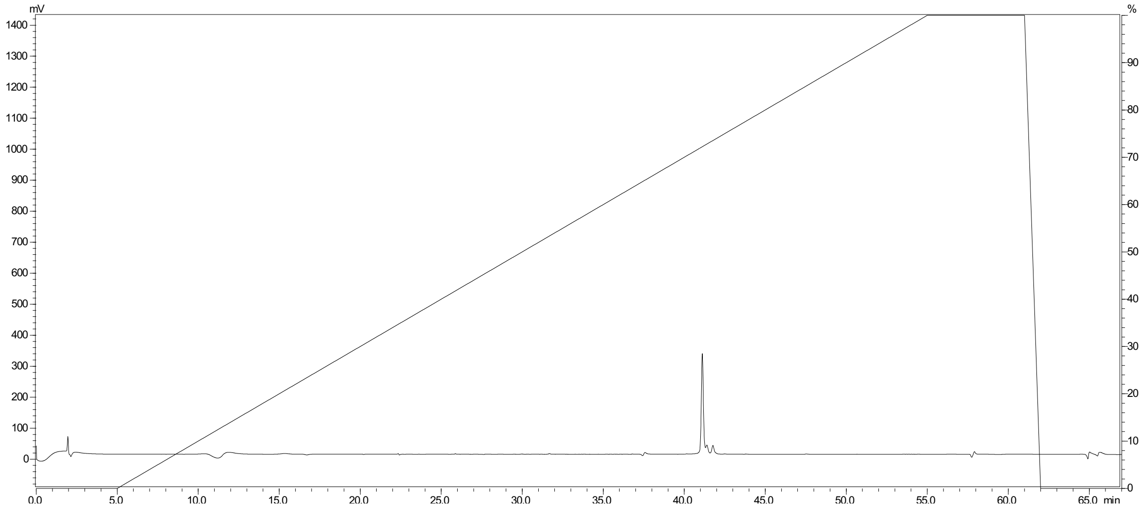

5(Ac)_pure_50min

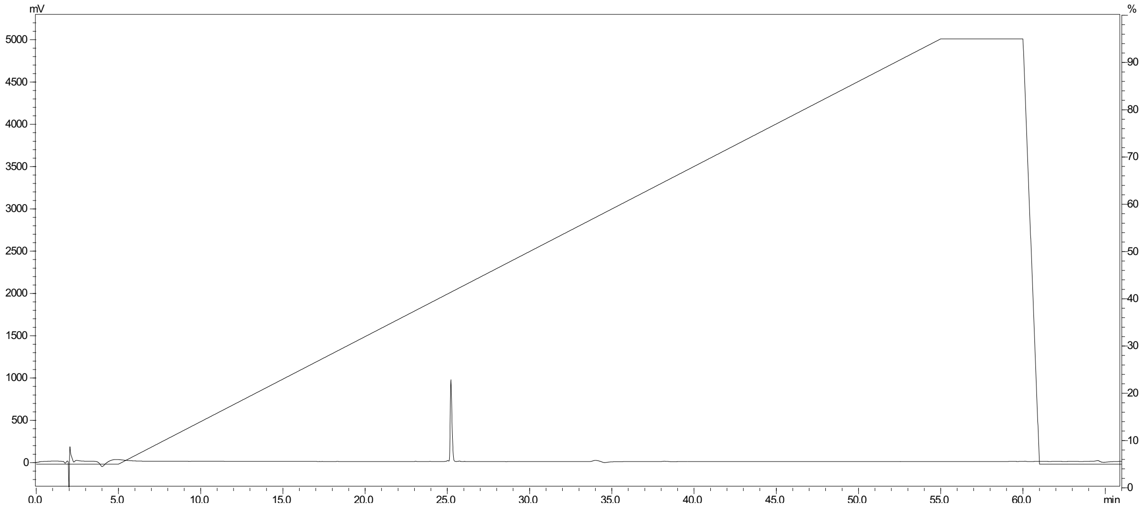

6(Ac)_pure_50min

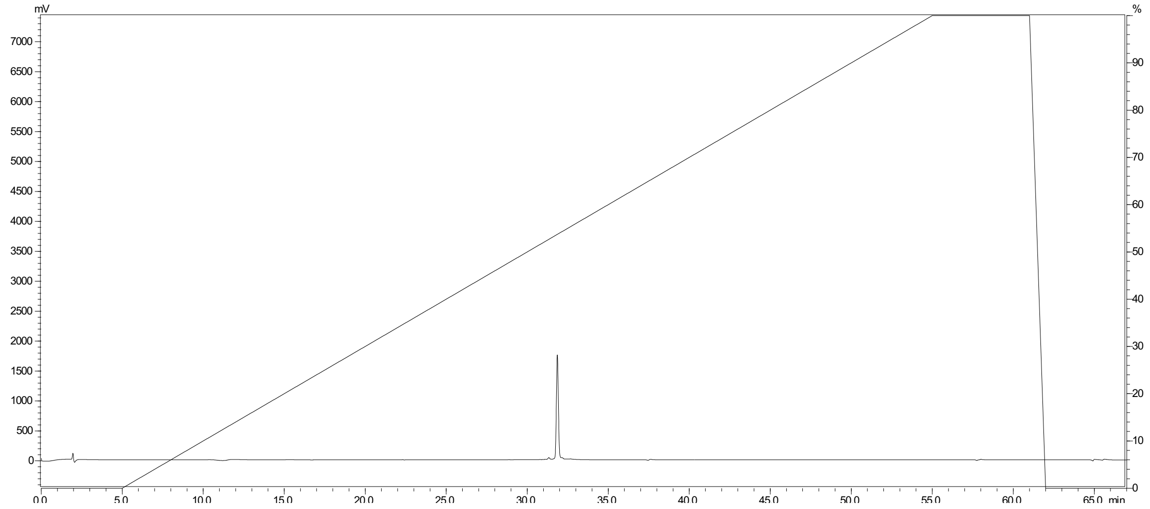

7(Ac)_pure_50min

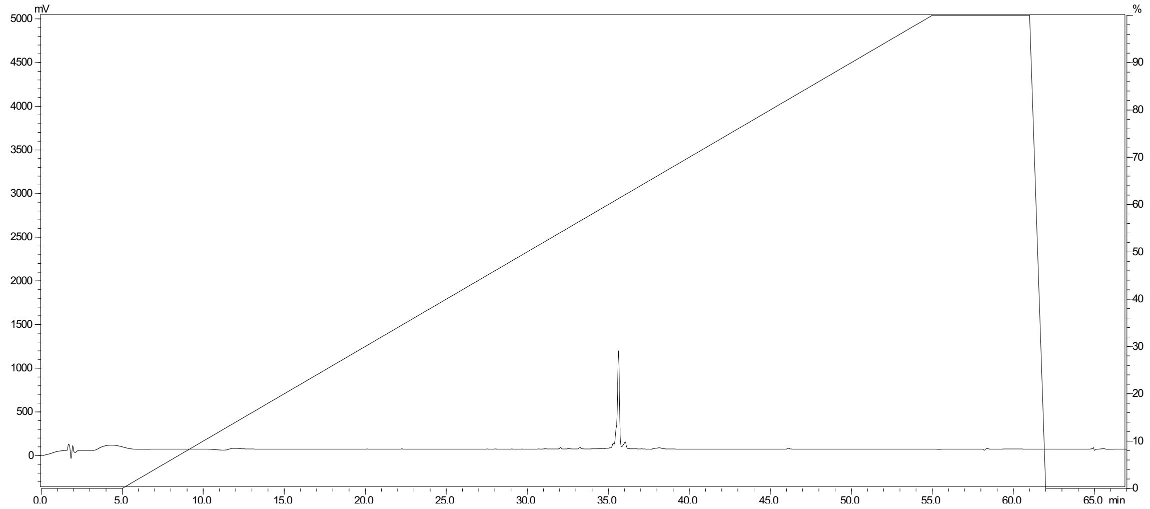

8(Ac)_pure_50min

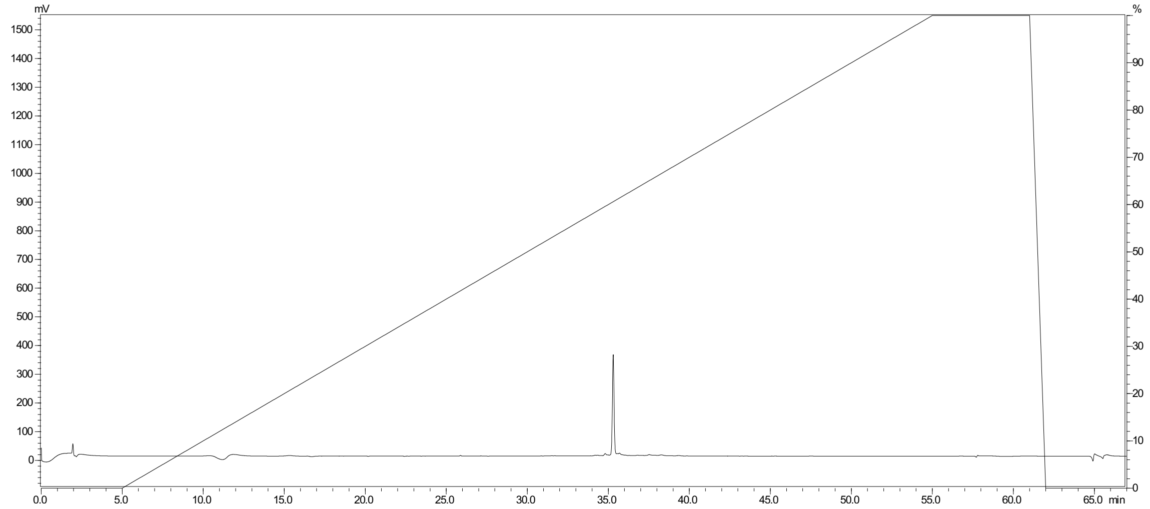

9(Ac)_pure_50min

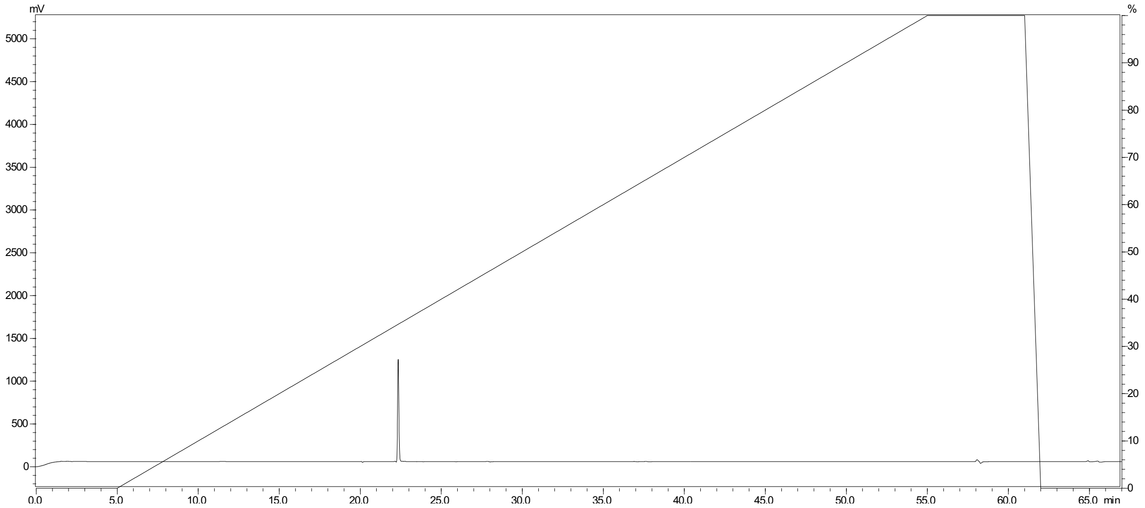

10(Ac)_pure_50min

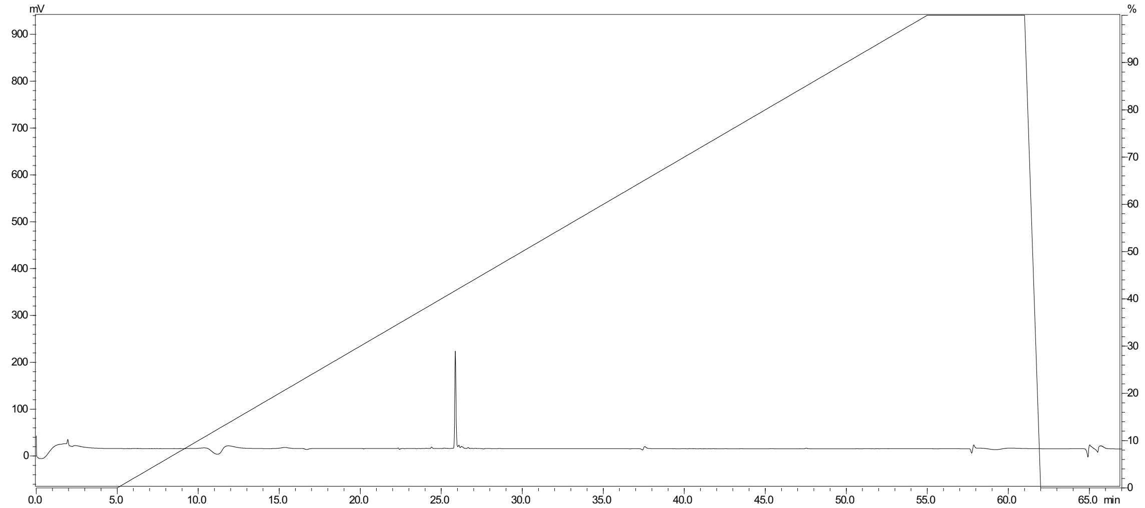

11(Ac)_pure_50min

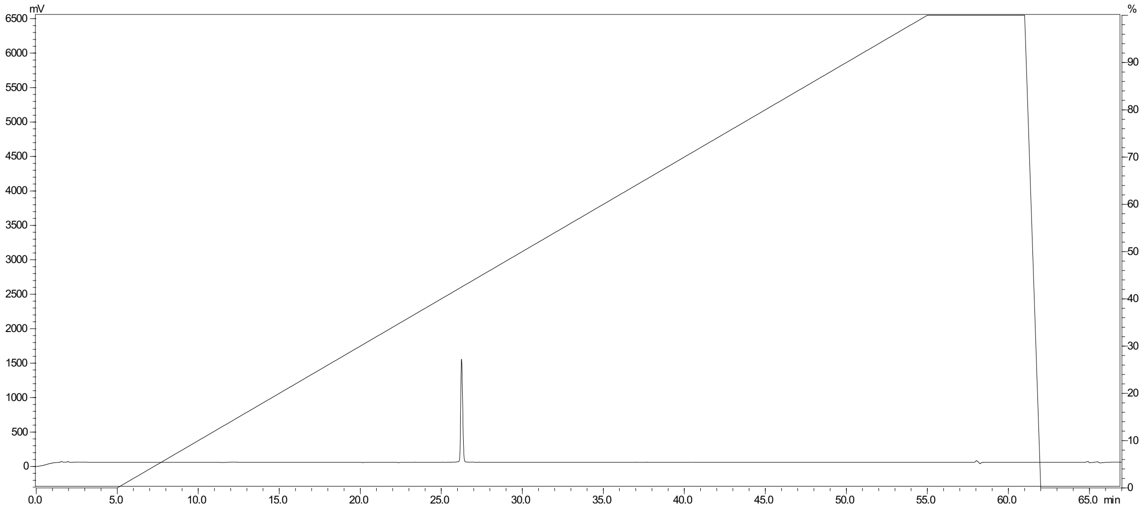

12(Ac)_pure_50min

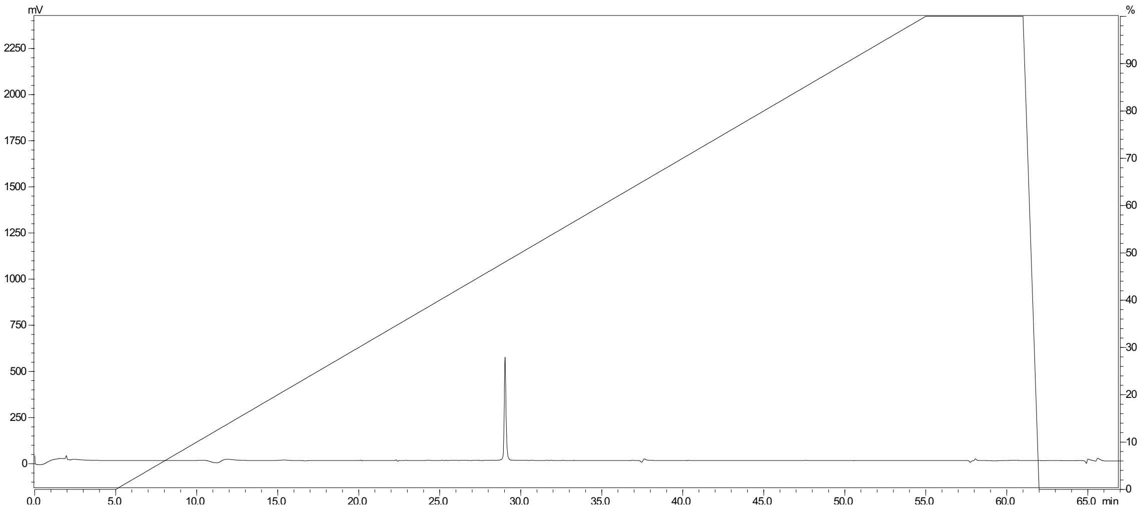

13(Ac)_pure_50min

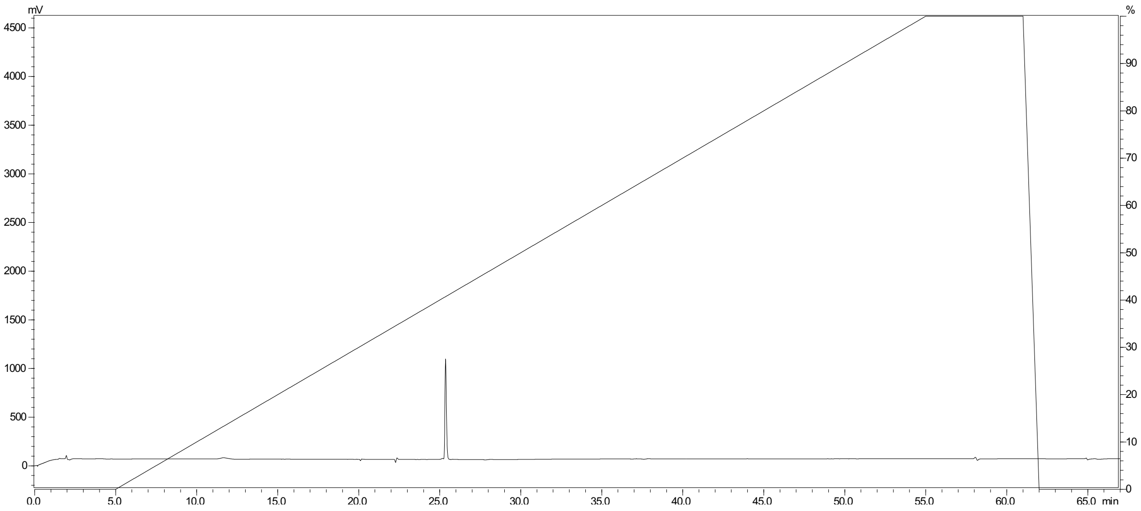

14(Ac)_pure_50min

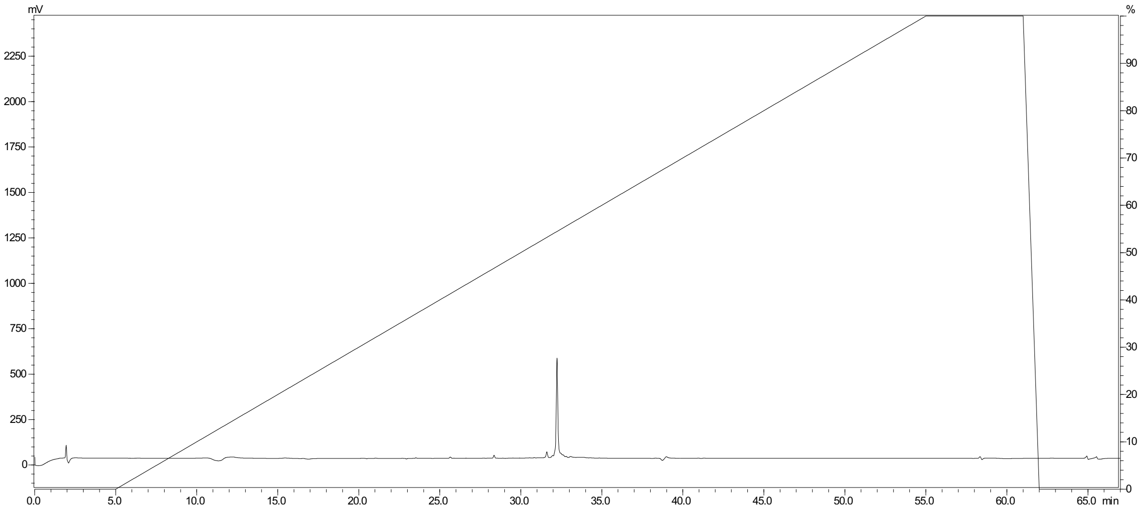

15(Ac)_pure_50min

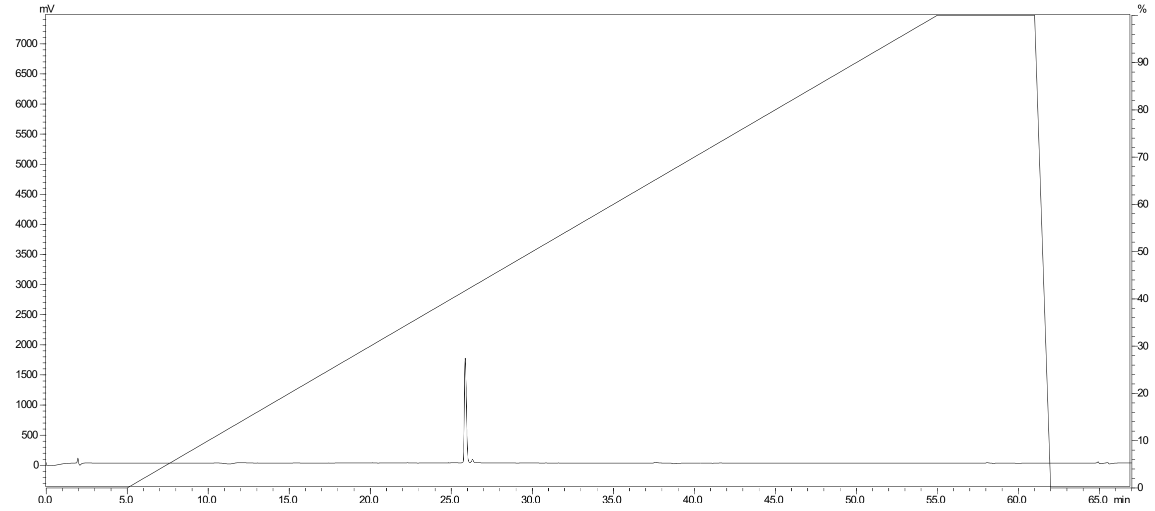

Scrambled(Ac)_pure_50min

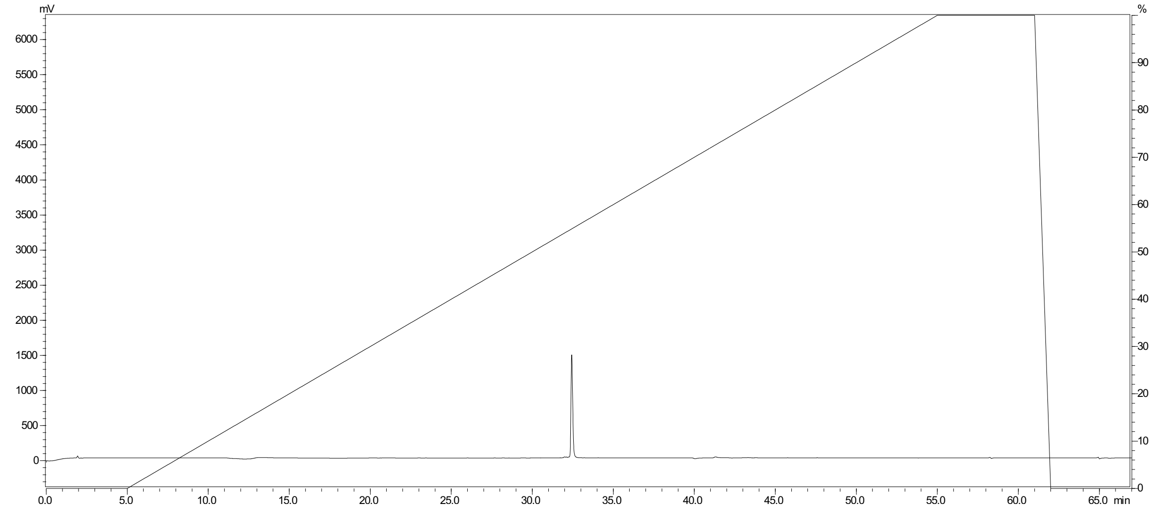

Negative Stapled(Ac)_pure_50min

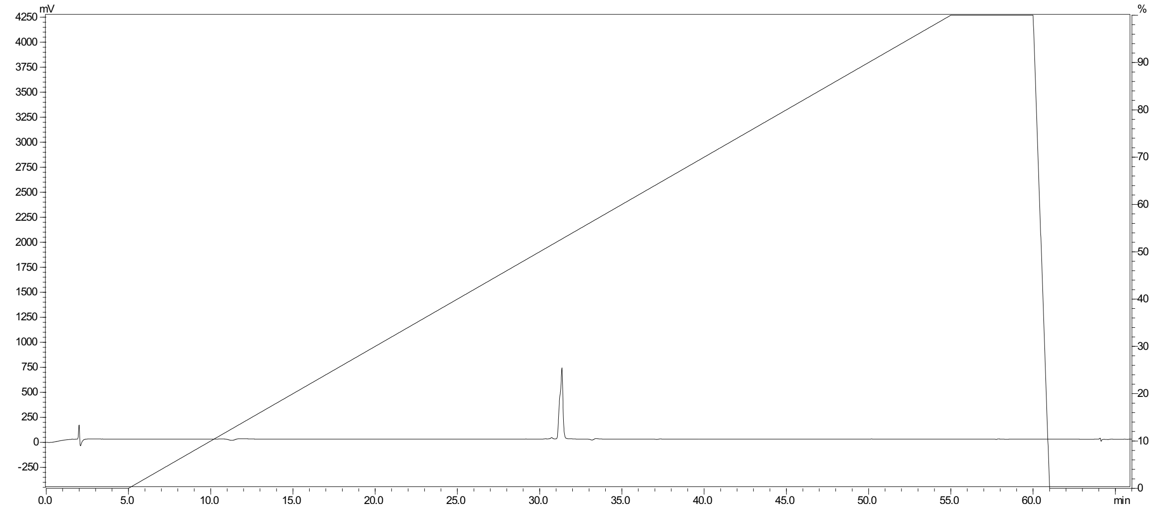

G-link(Ac)_pure_50min

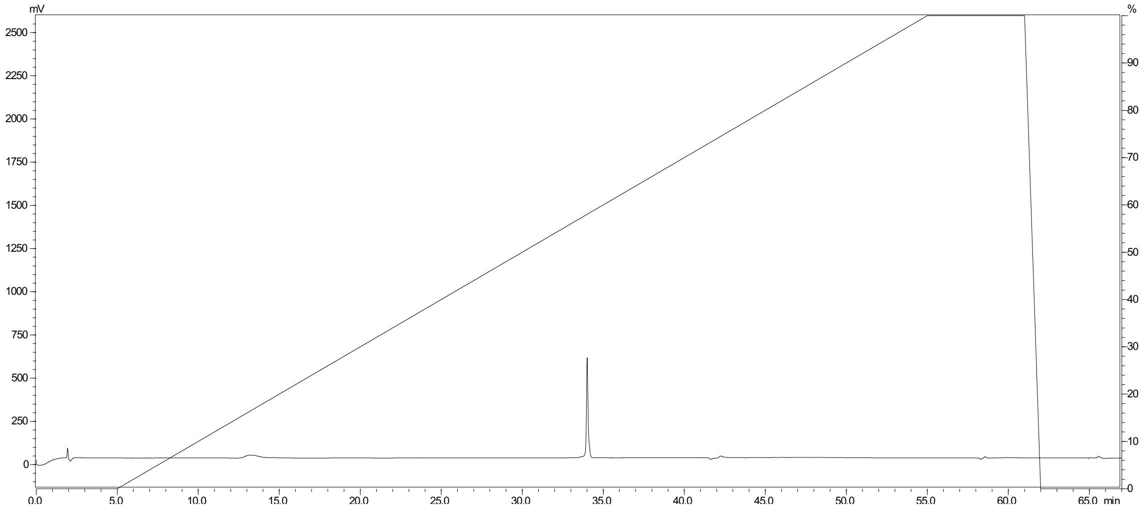

D30E(Ac)_pure_50min

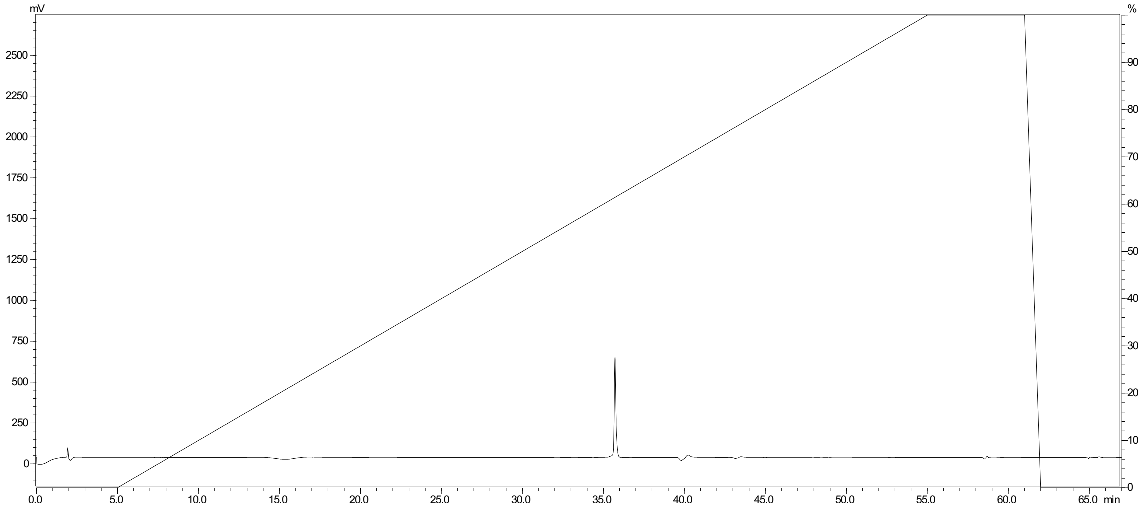

G-link Stapled(Ac)_pure_50min

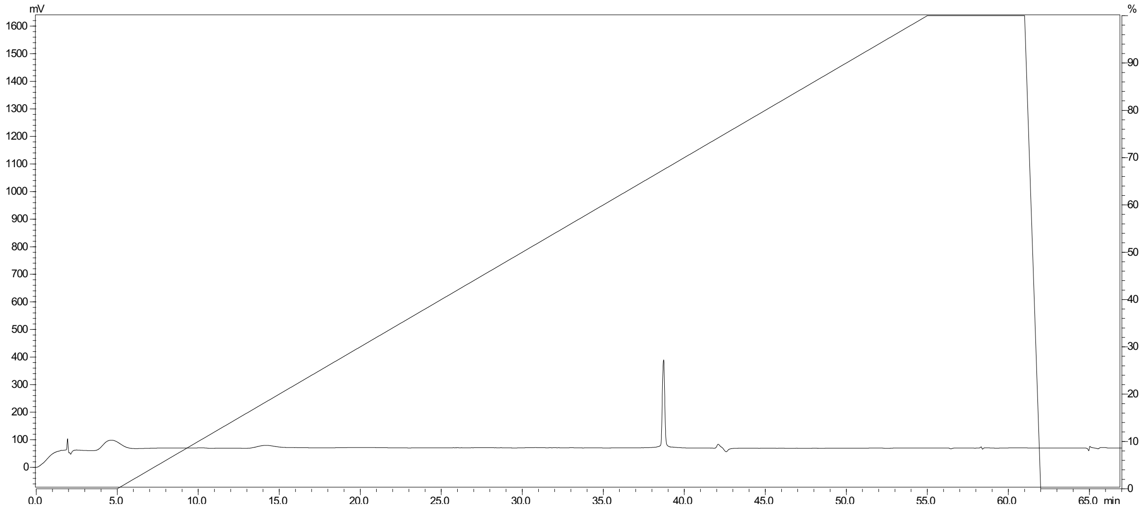

Mutant(Ac)_pure_50min

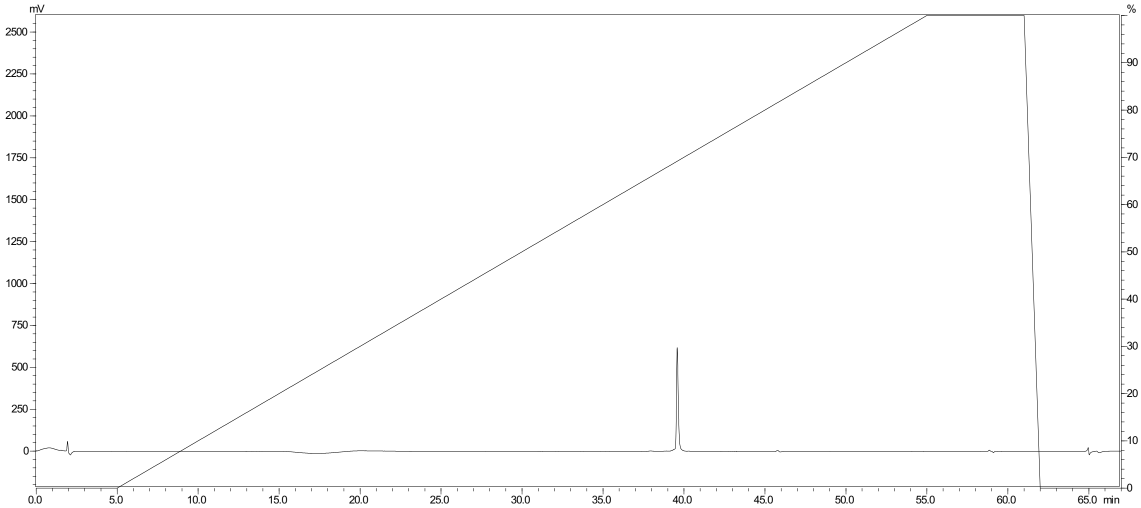

FITC Peptides HPLC Traces:

additional peaks due to FITC isomers

1(FITC)_pure_50min

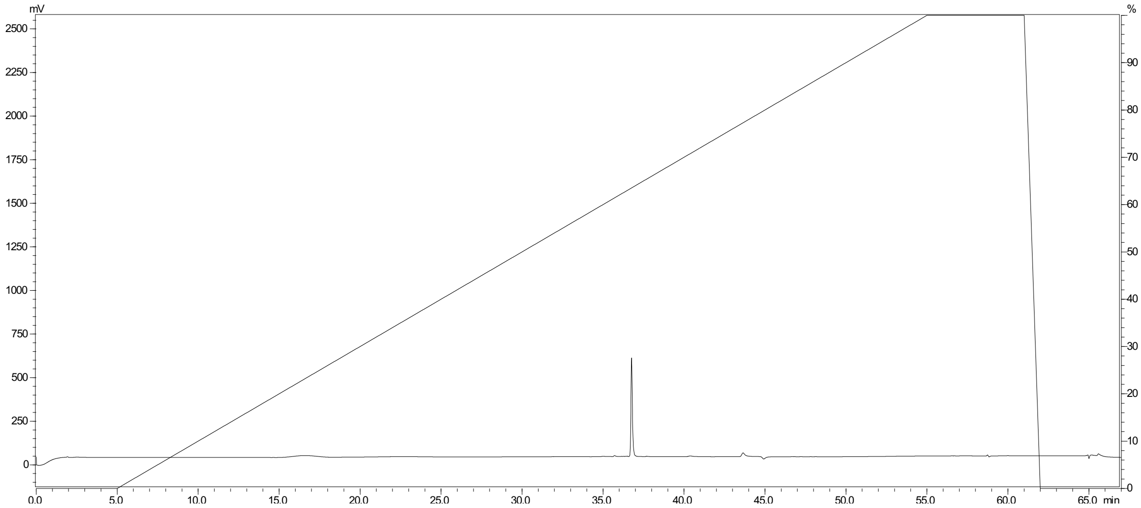

2(FITC)_pure_50min

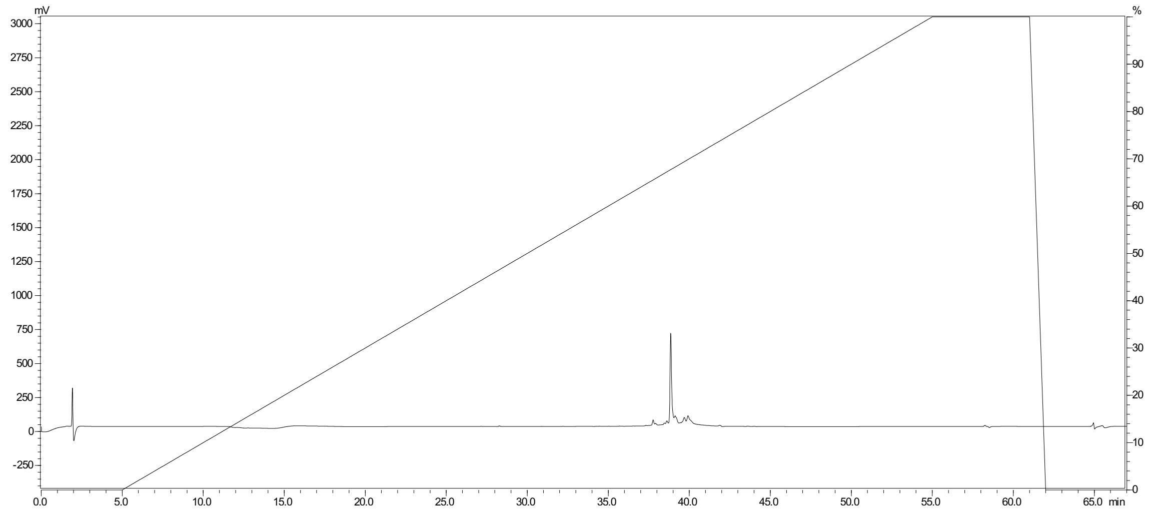

3(FITC)_pure_50min

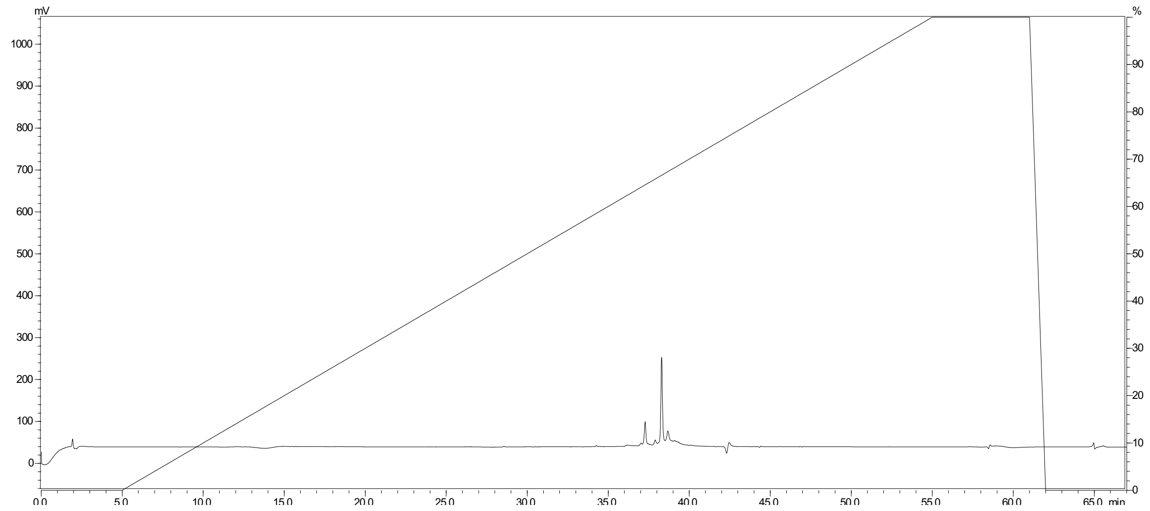

4(FITC)_pure_50min

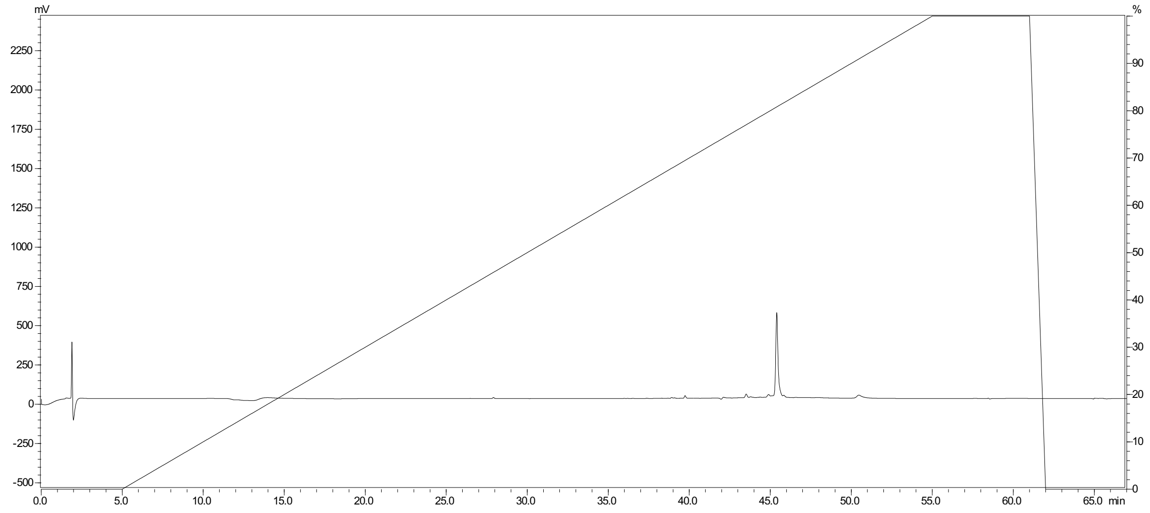

5(FITC)_pure_50min

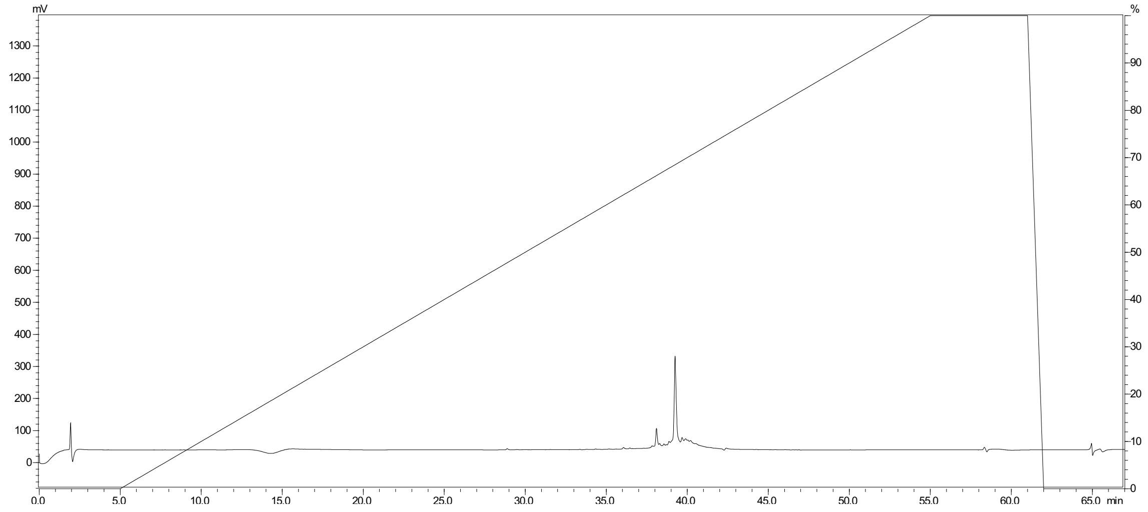

6(FITC)_pure_50min

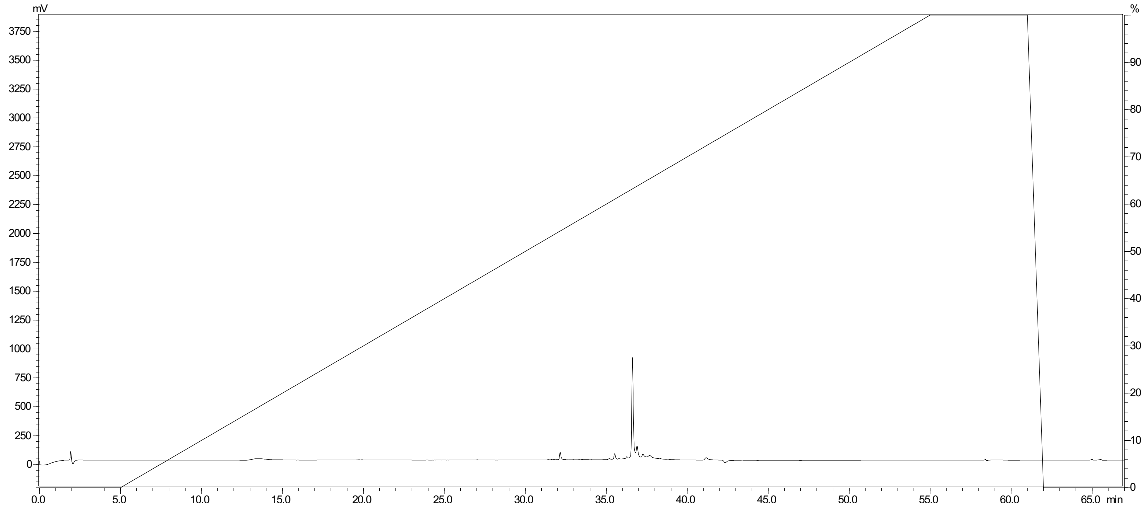

7(FITC)_pure_50min

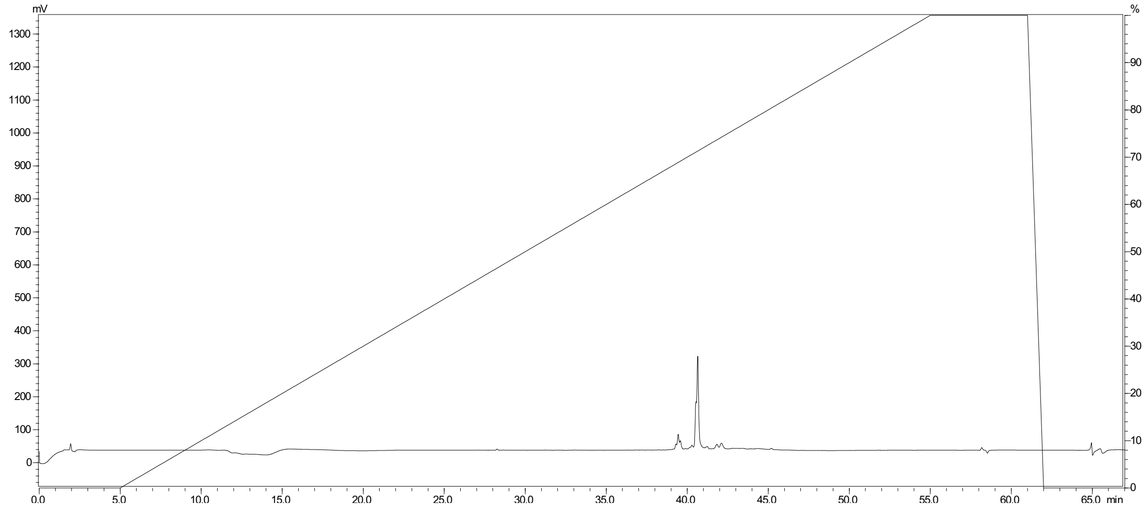

8(FITC)_pure_50min

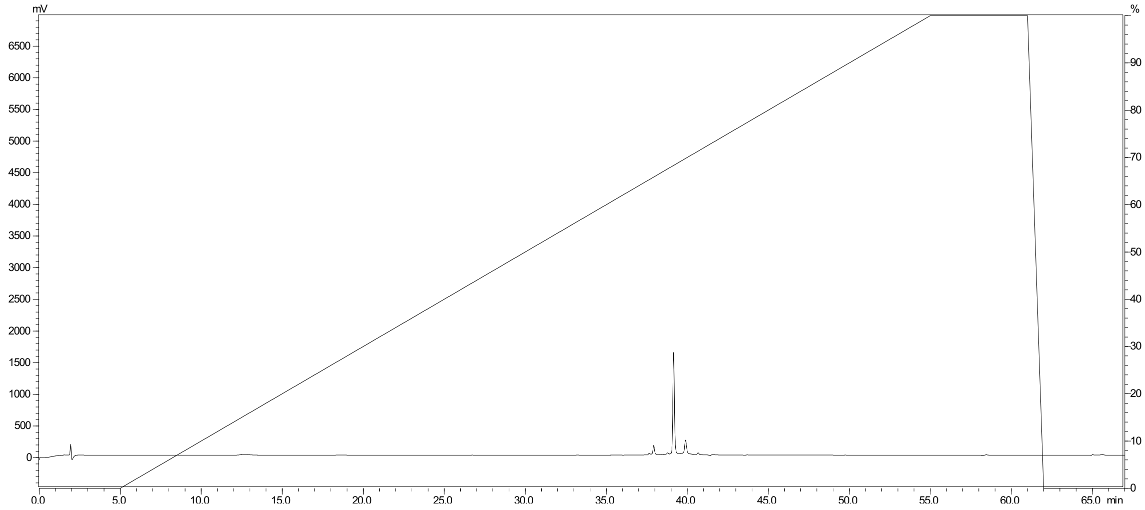

9(FITC)_pure_50min

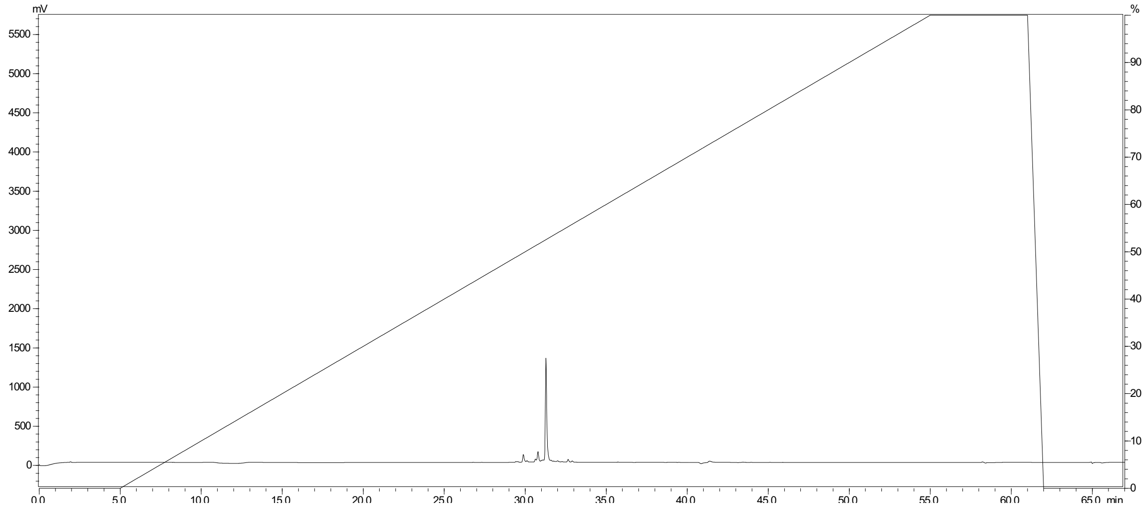

10(FITC)_pure_50min

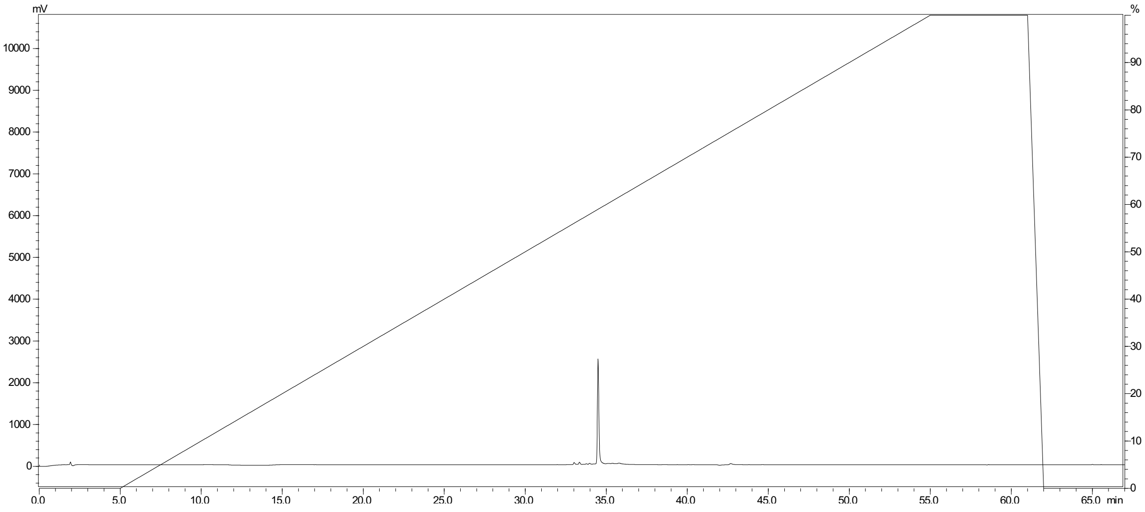

11(FITC)_pure_50min

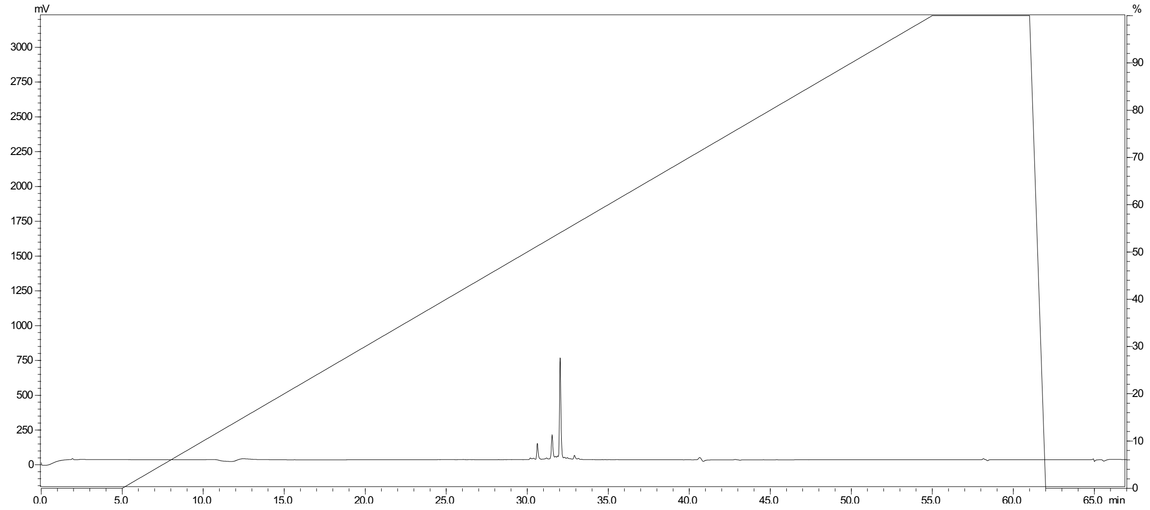

12(FITC)_pure_50min

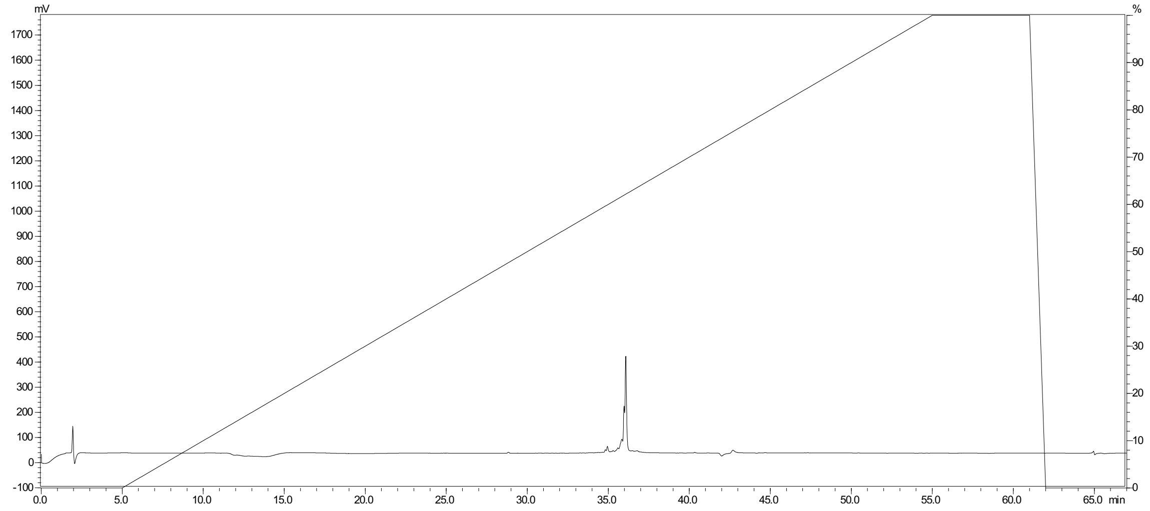

13(FITC)_pure_50min

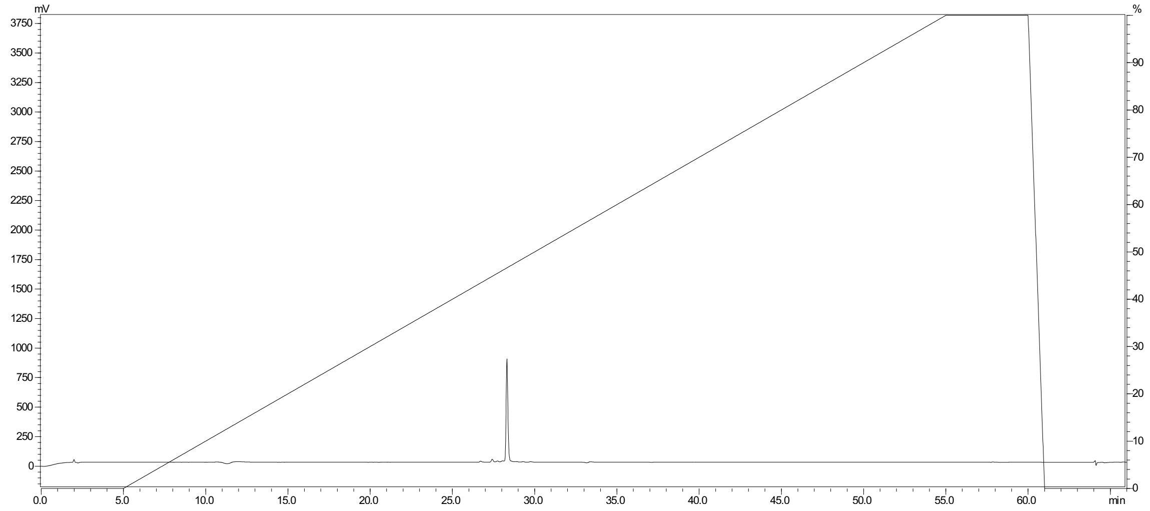

14(FITC)_pure_50min

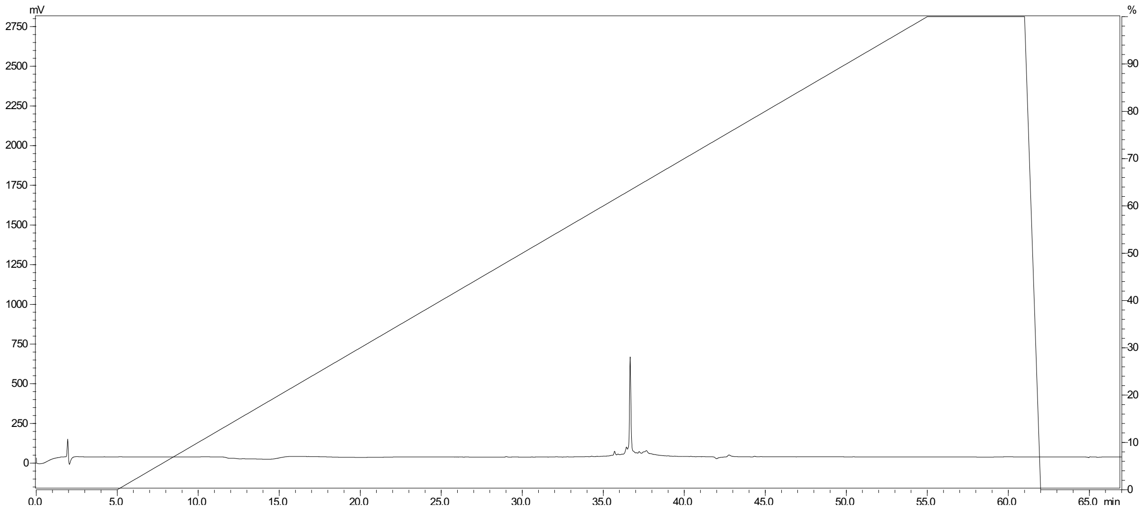

15(FITC)_pure_50min

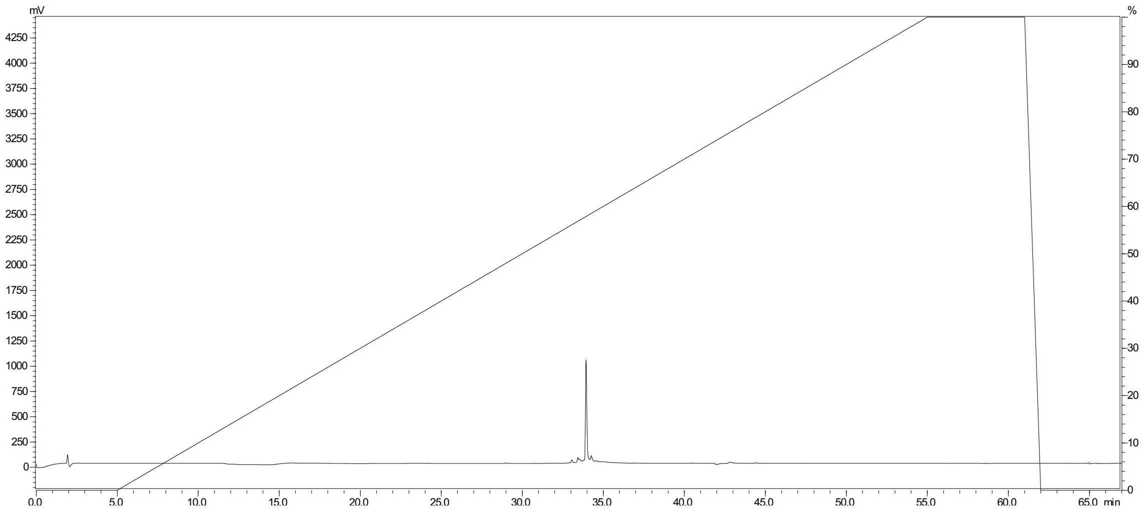

Scrambled(FITC)_pure_50min

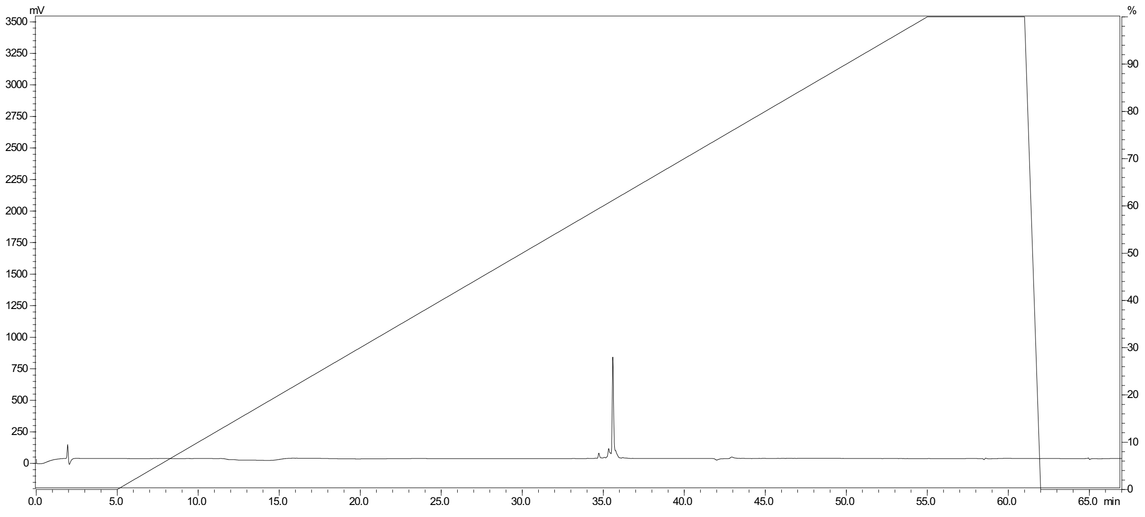

Negative Stapled(FITC)_pure_50min

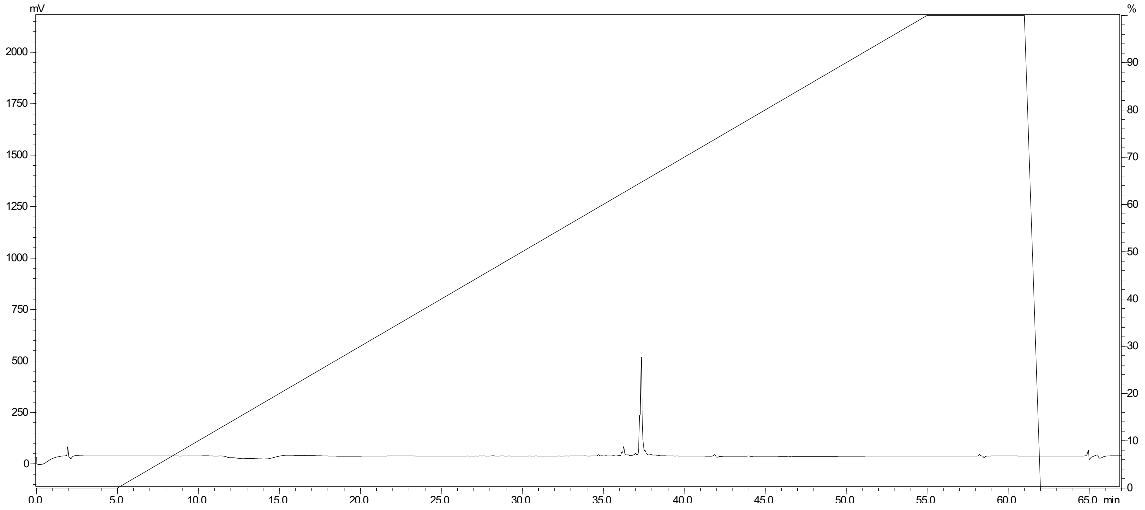

G-link(FITC)_pure_50min

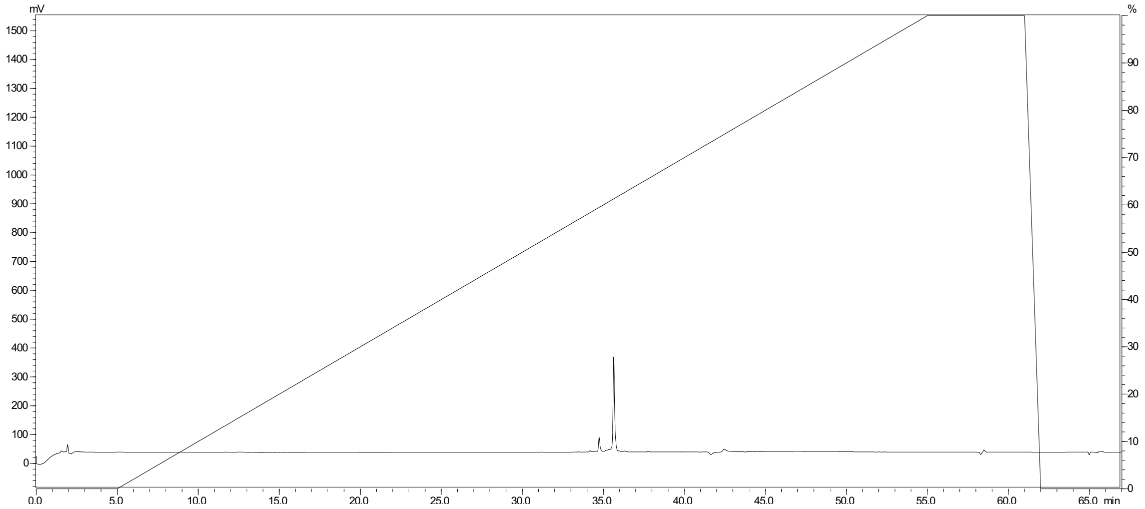

D30E(FITC)_pure_50min

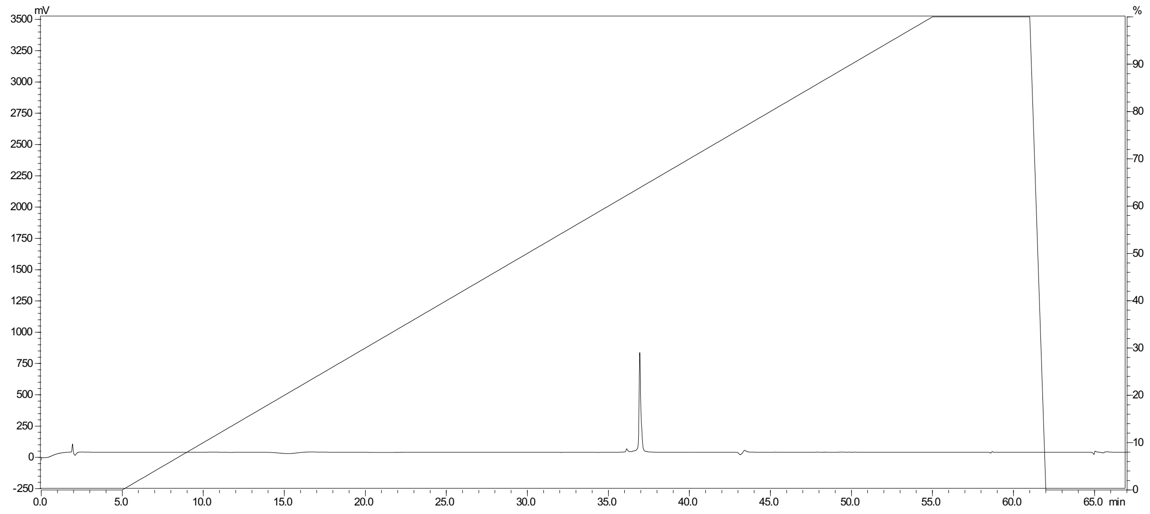

G-link Stapled(FITC)_pure_50min

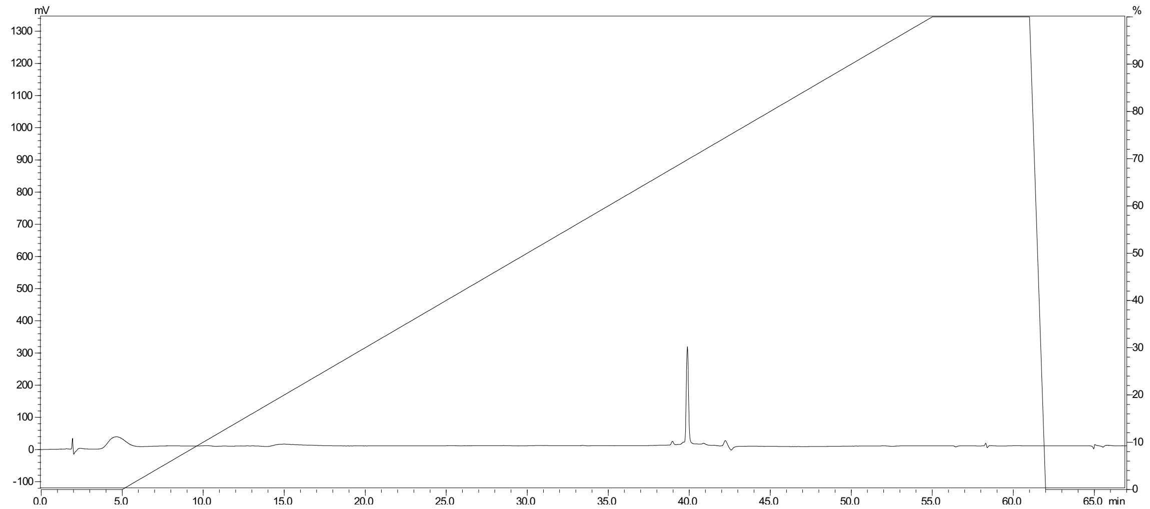

1(C-terminal_FITC)_pure_50min

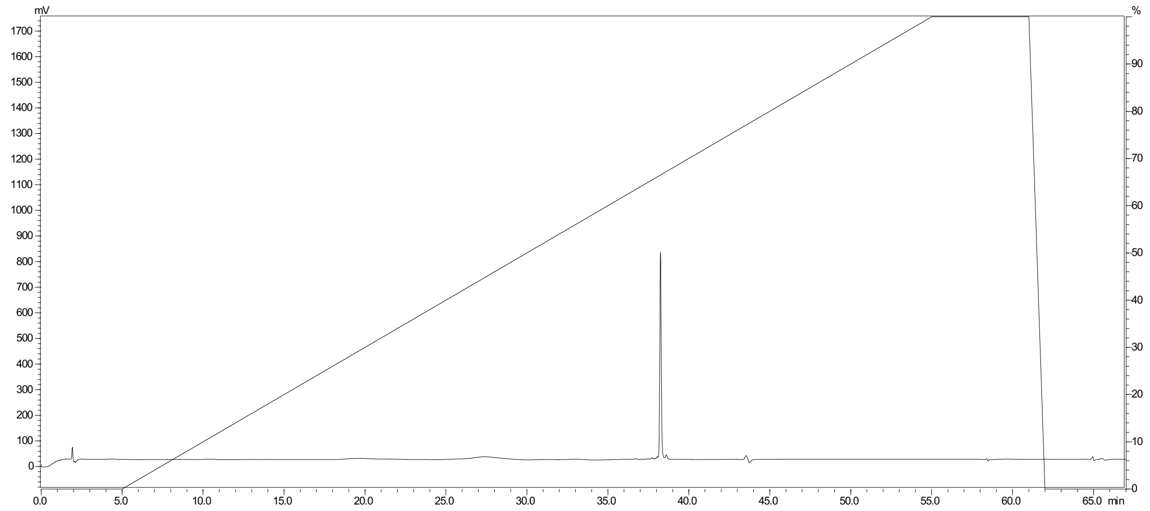

Scrambled(C-terminal_FITC)_pure_50min

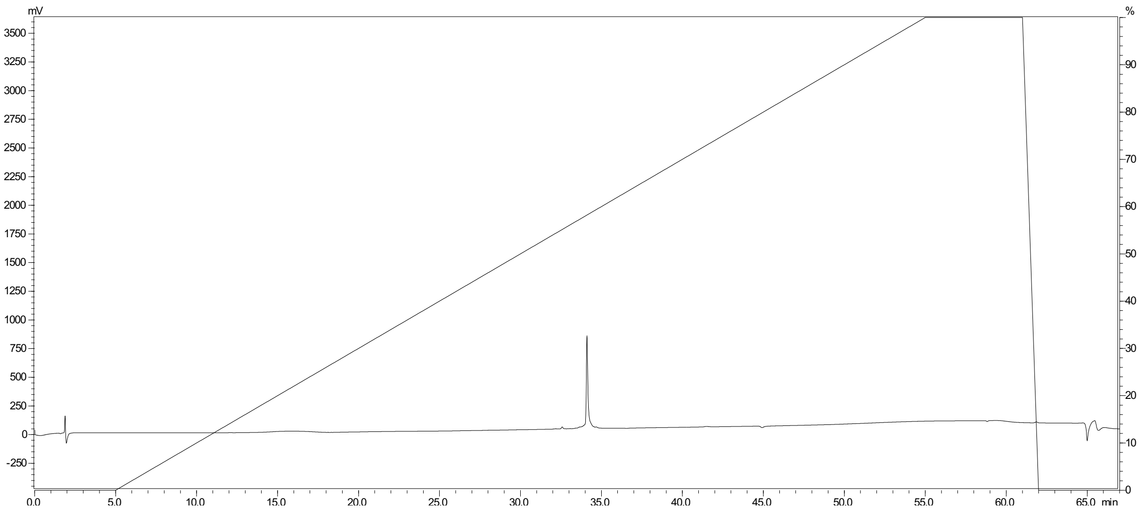

Acetylated Peptides LCMS Traces:

1(Ac)_pure

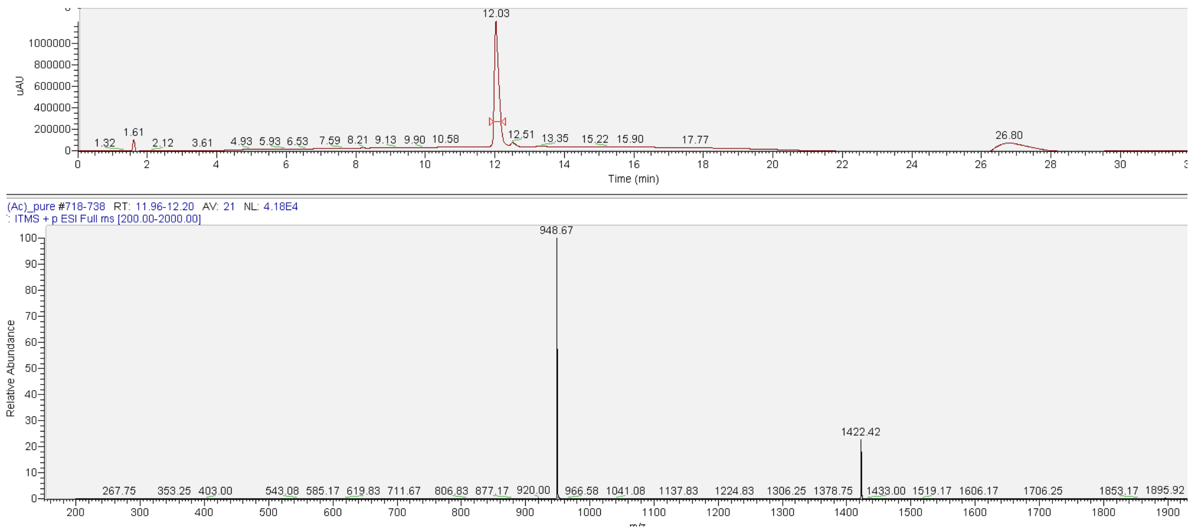

2(Ac)_pure

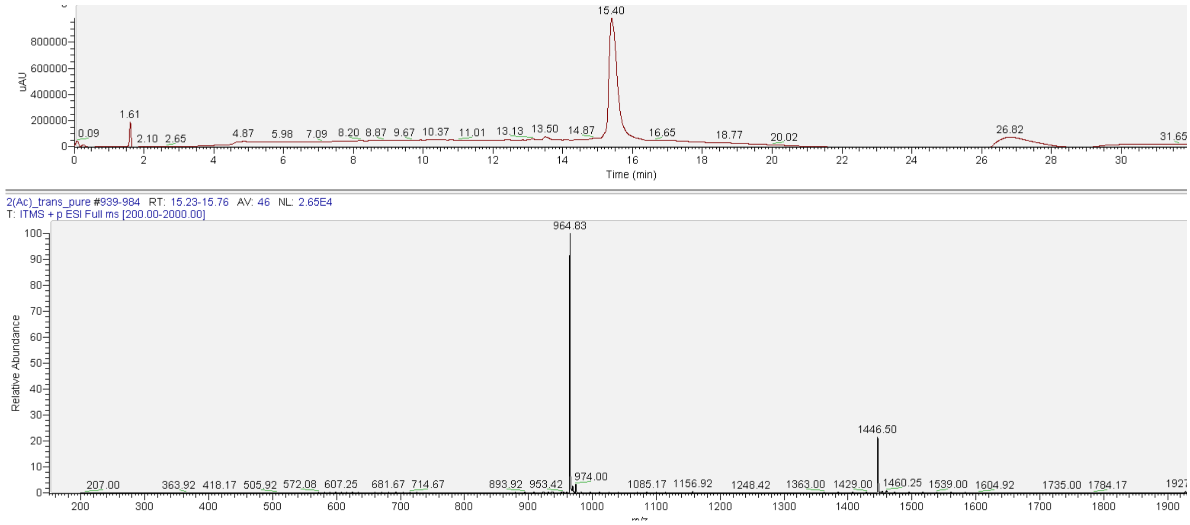

3(Ac)_pure

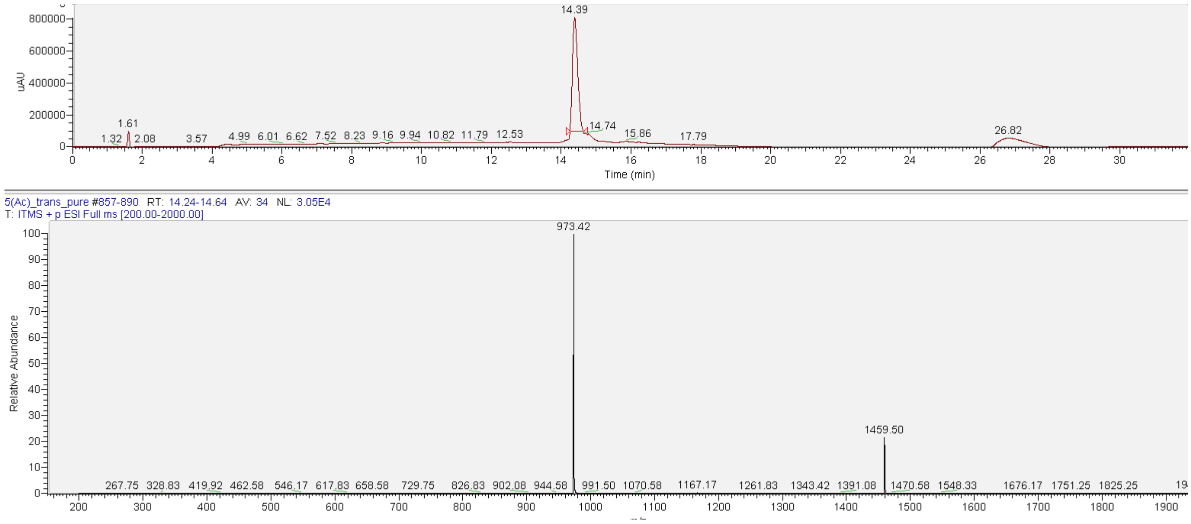

4(Ac)_pure

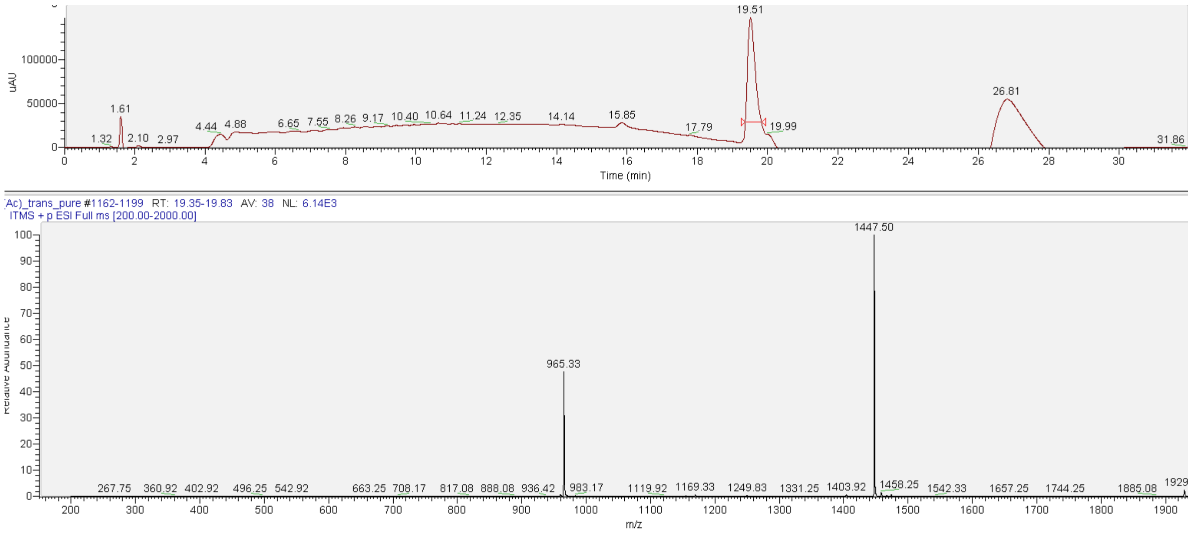

5(Ac)_pure

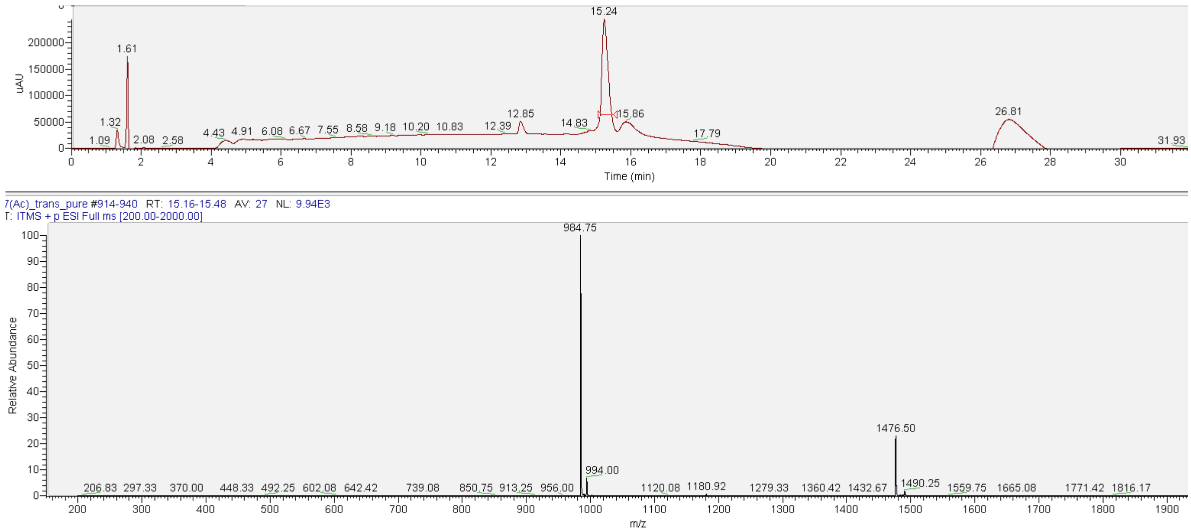

6(Ac)_pure

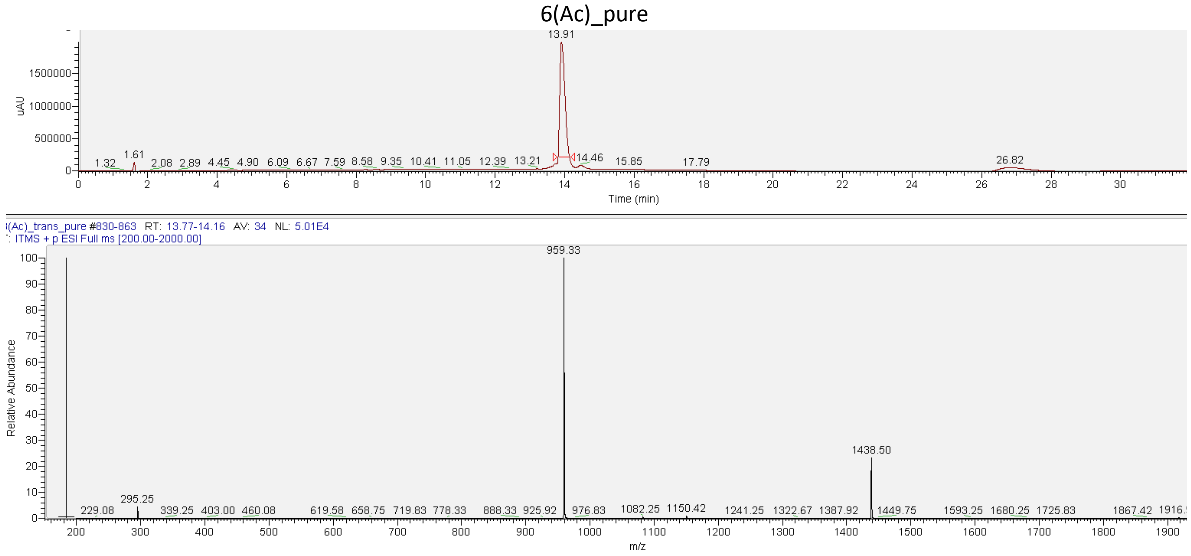

7(Ac)_pure

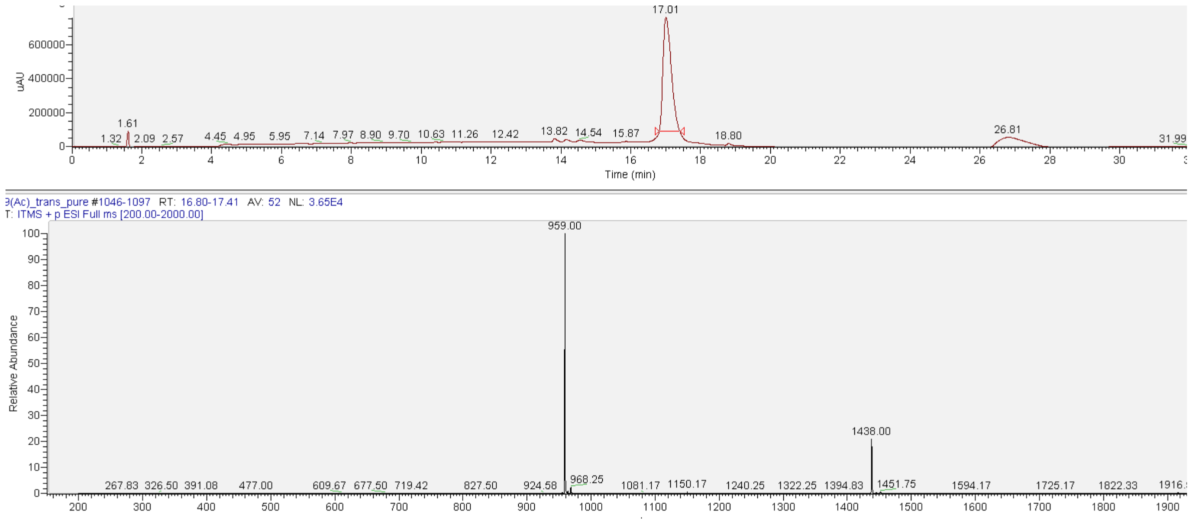

8(Ac)_pure

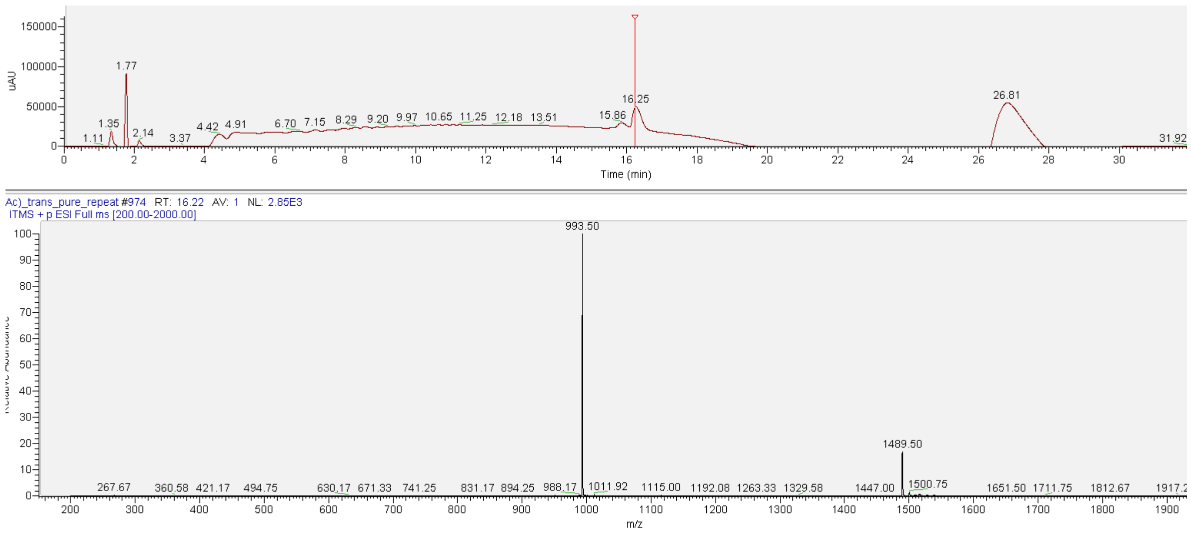

9(Ac)_pure

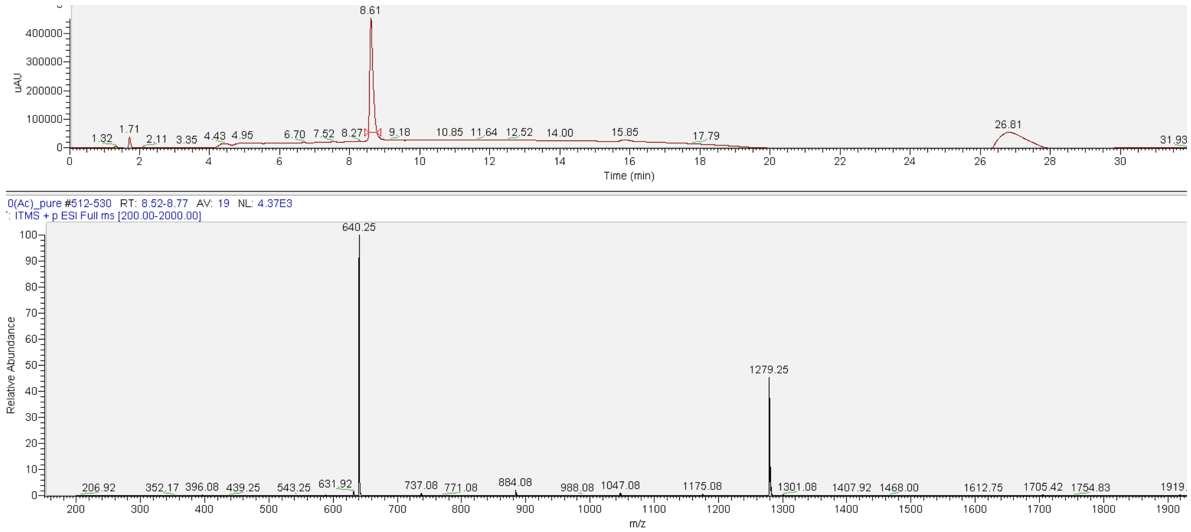

10(Ac)_pure

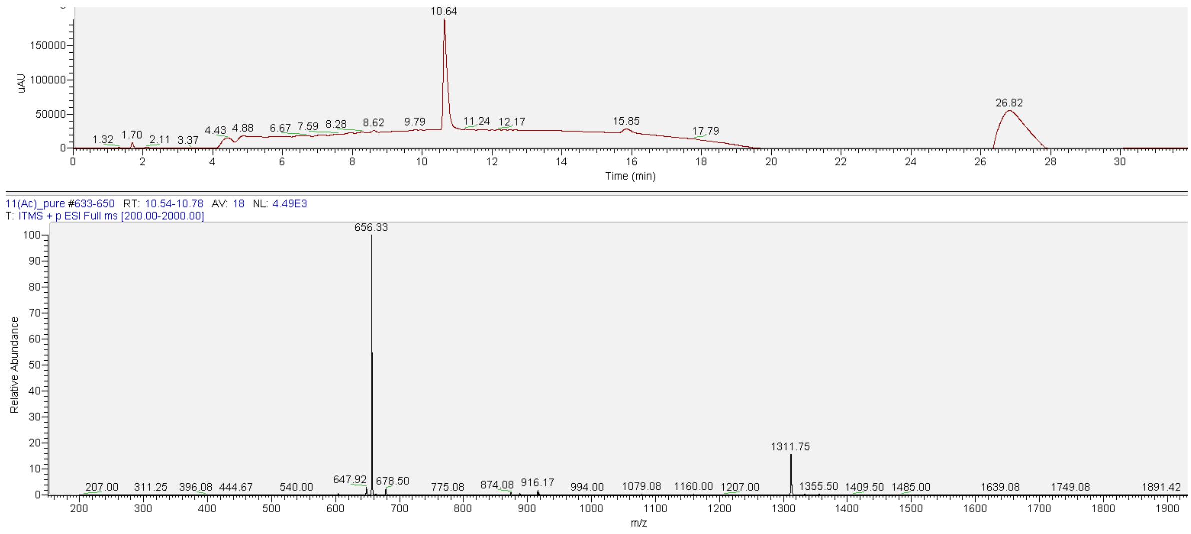

11(Ac)_pure

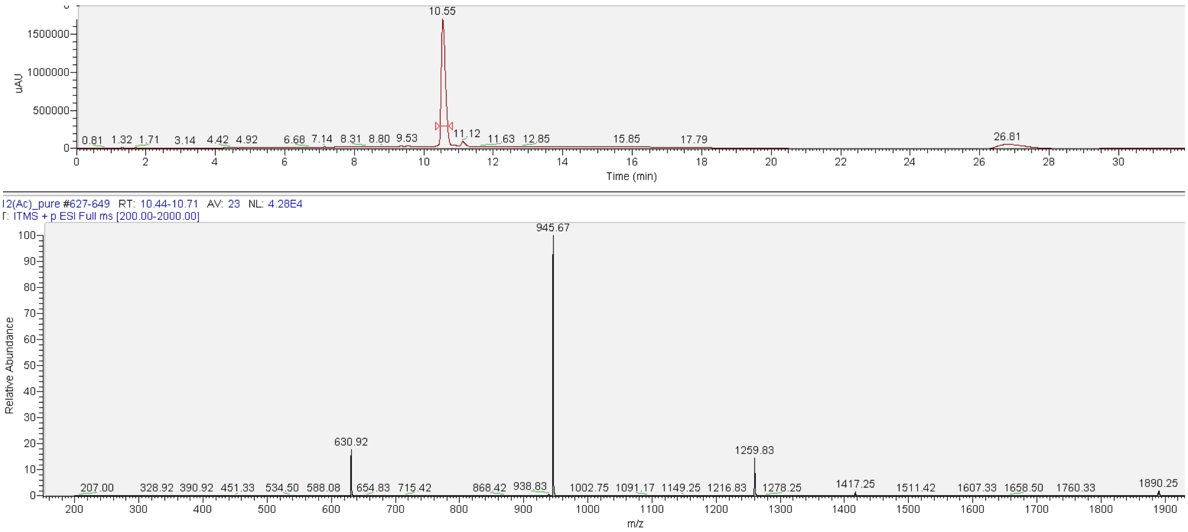

12(Ac)_pure

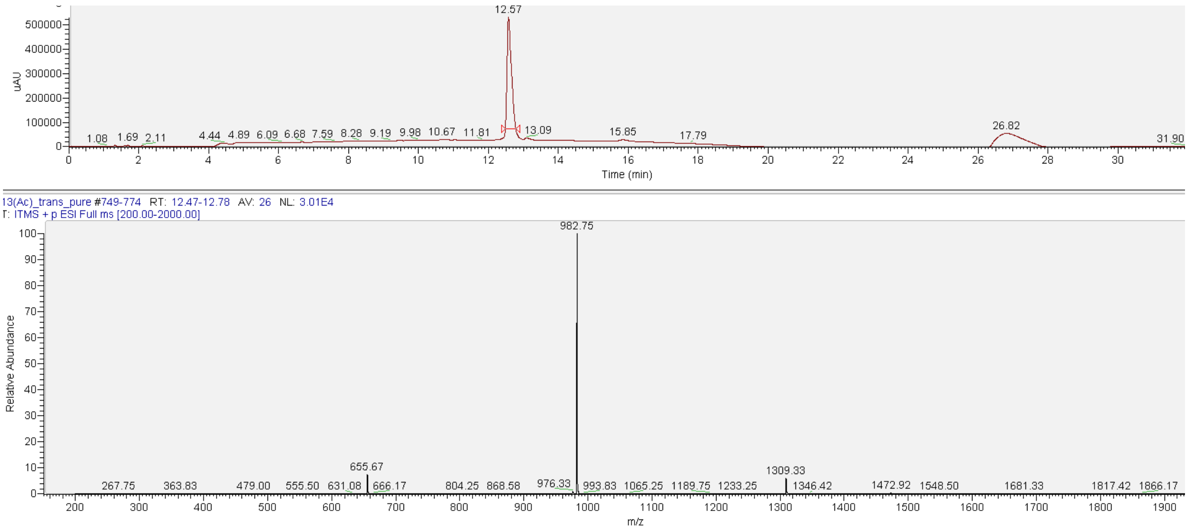

13(Ac)_pure

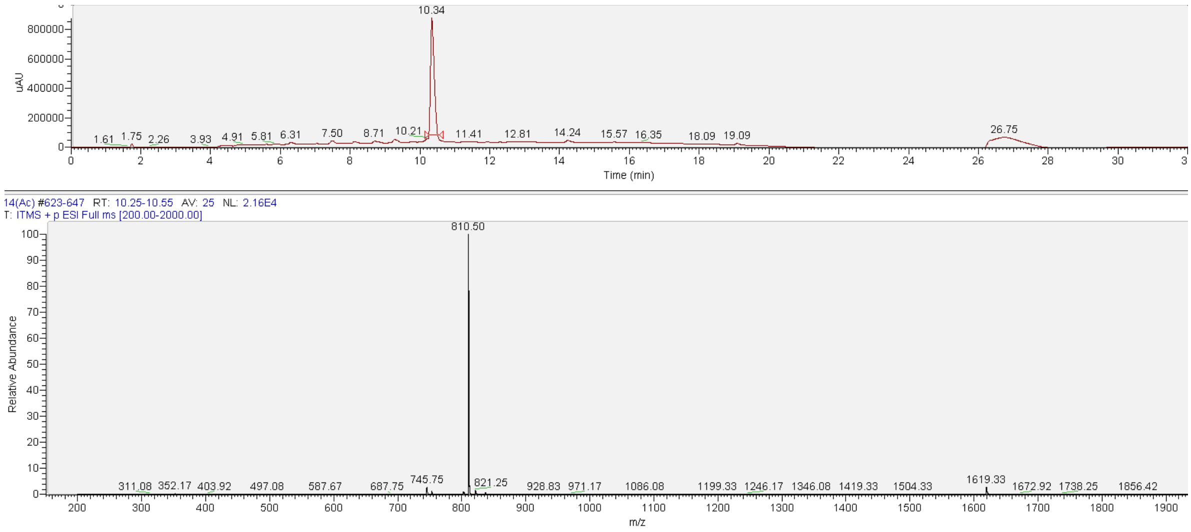

14(Ac)_pure

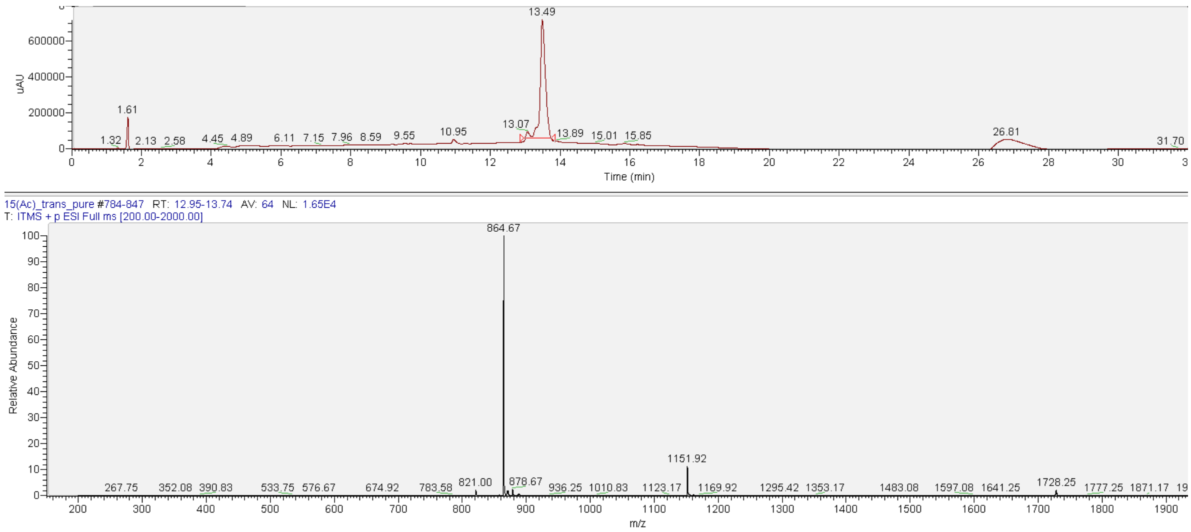

15(Ac)_pure

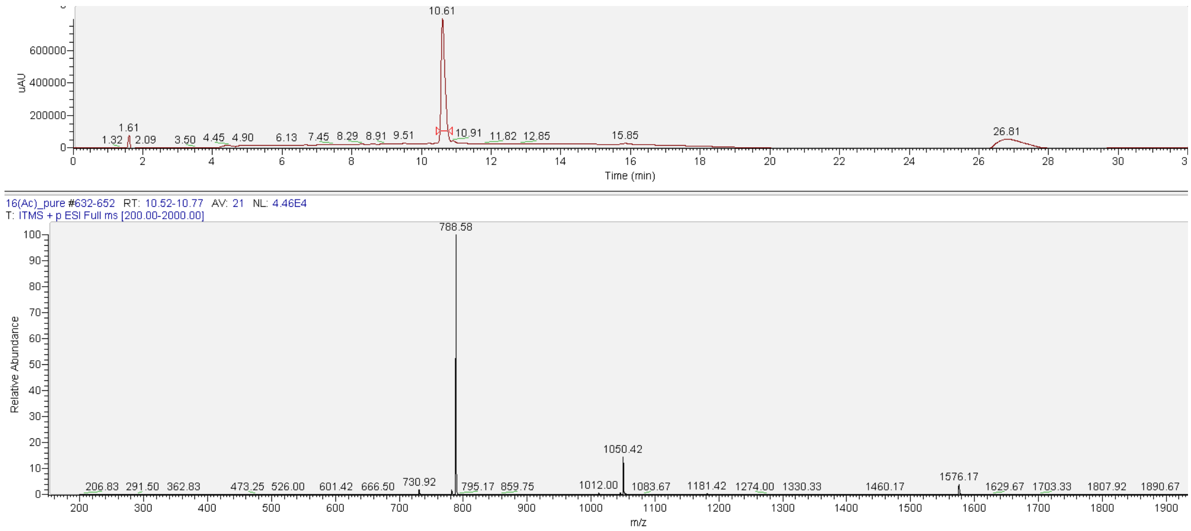

Scrambled(Ac)_pure

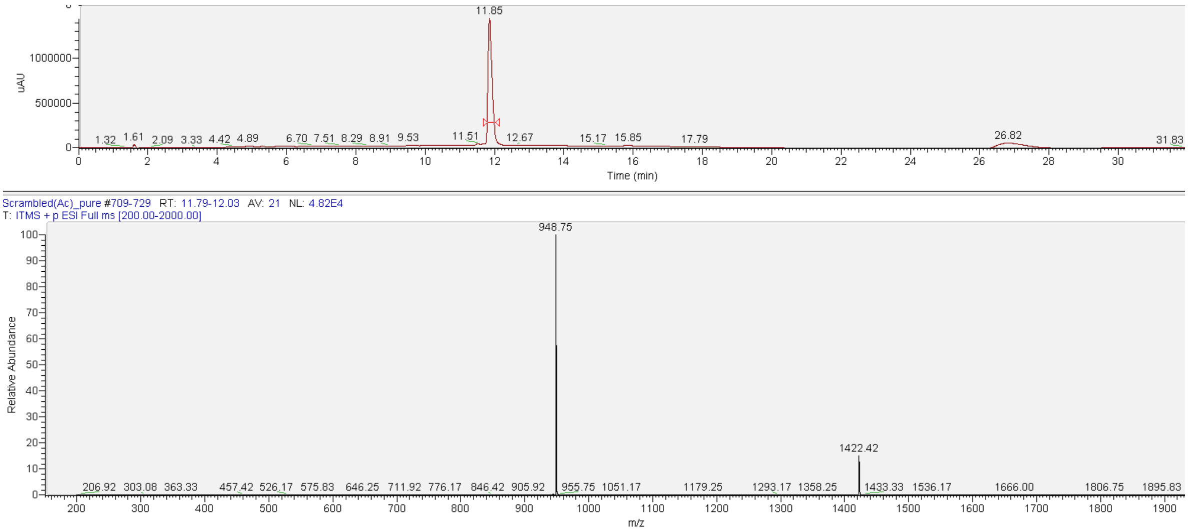

Negative Stapled(Ac)_pure

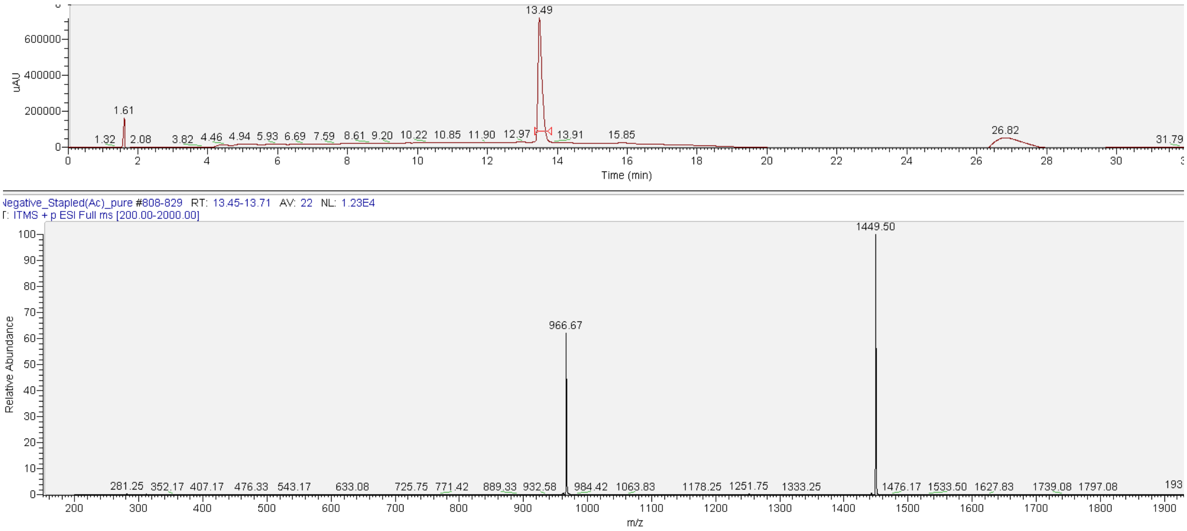

G-link(Ac)_pure

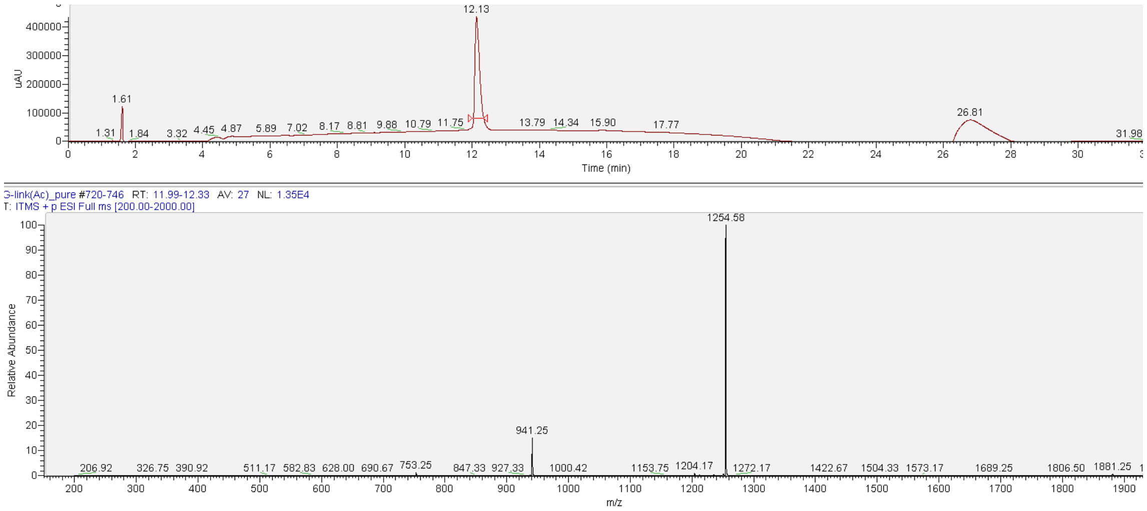

D30E(Ac)_pure

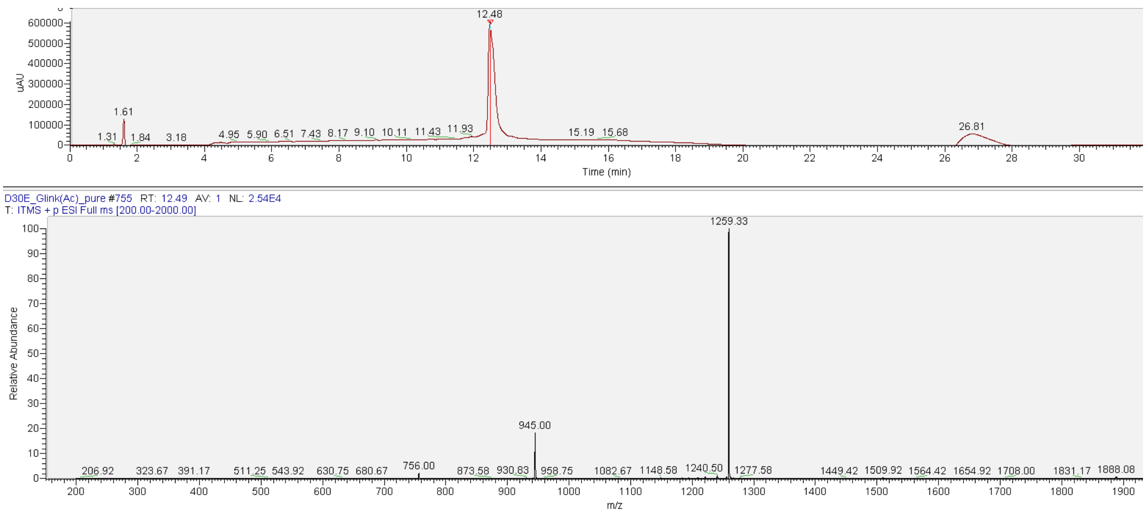

G-link Stapled(Ac)_pure

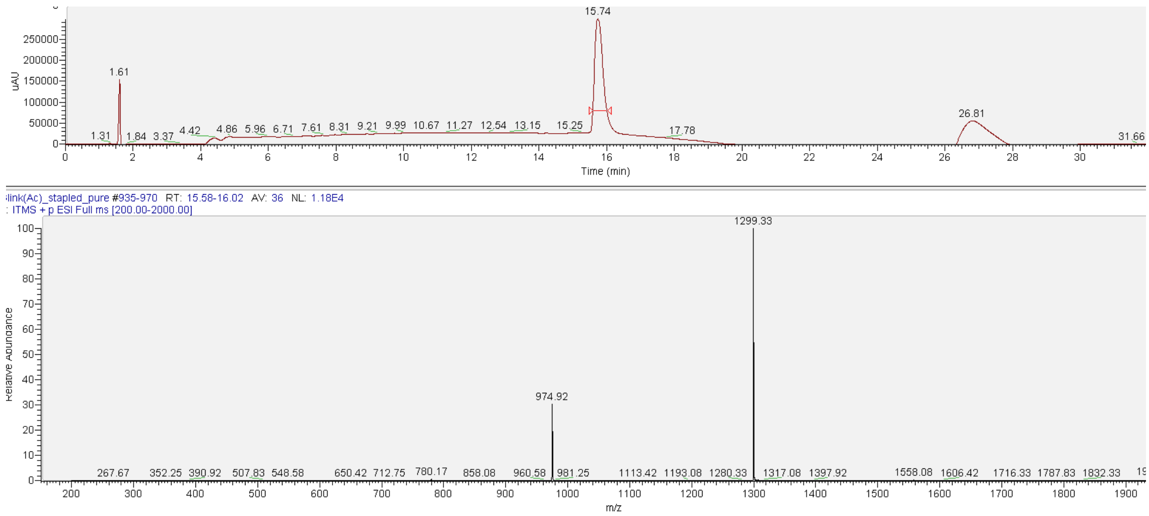

Mutant(Ac)_pure

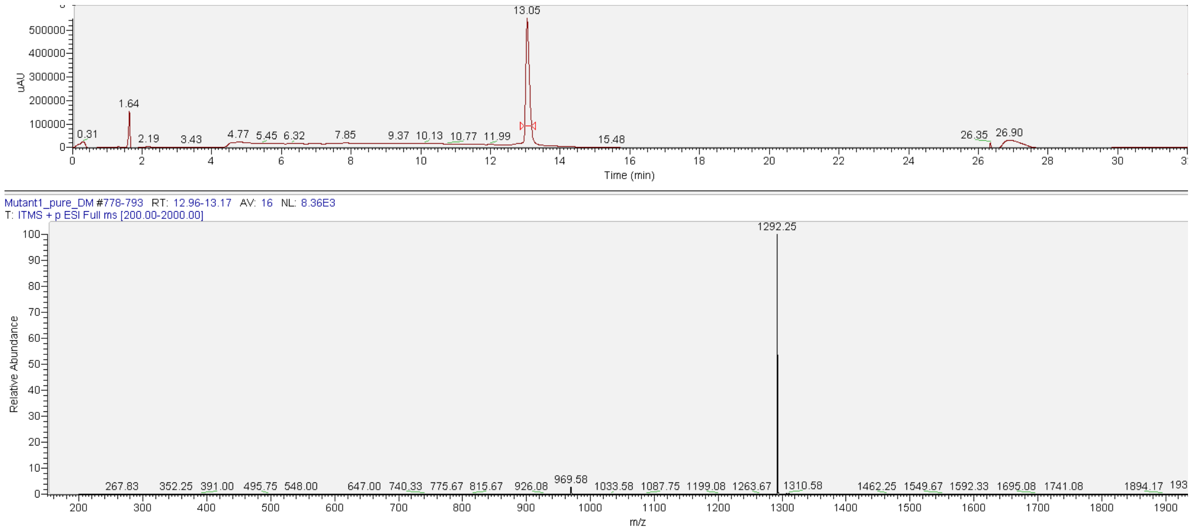

FITC Peptides LCMS Traces:

1(FITC)_pure

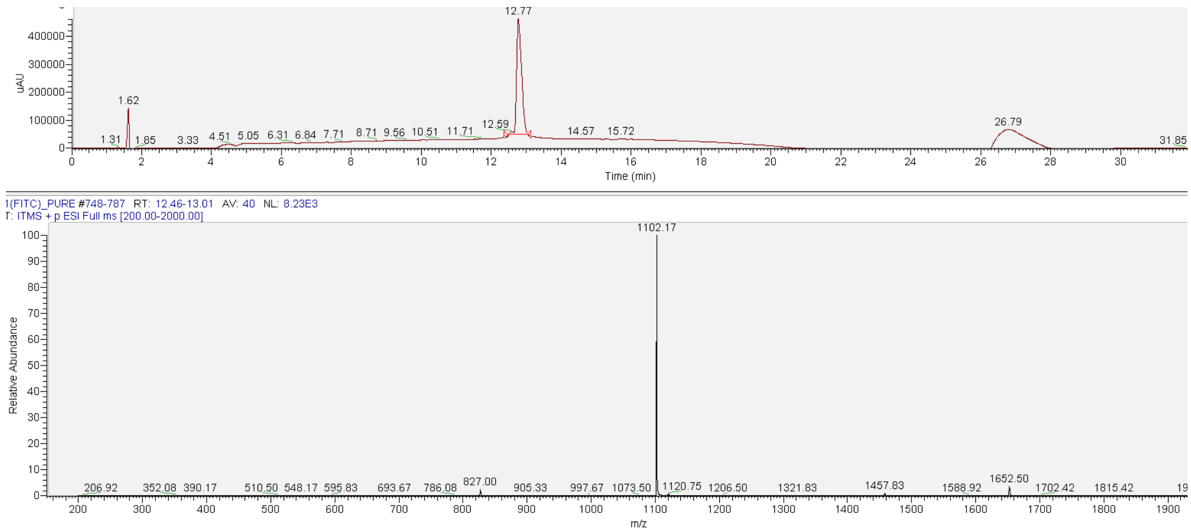

2(FITC)_pure

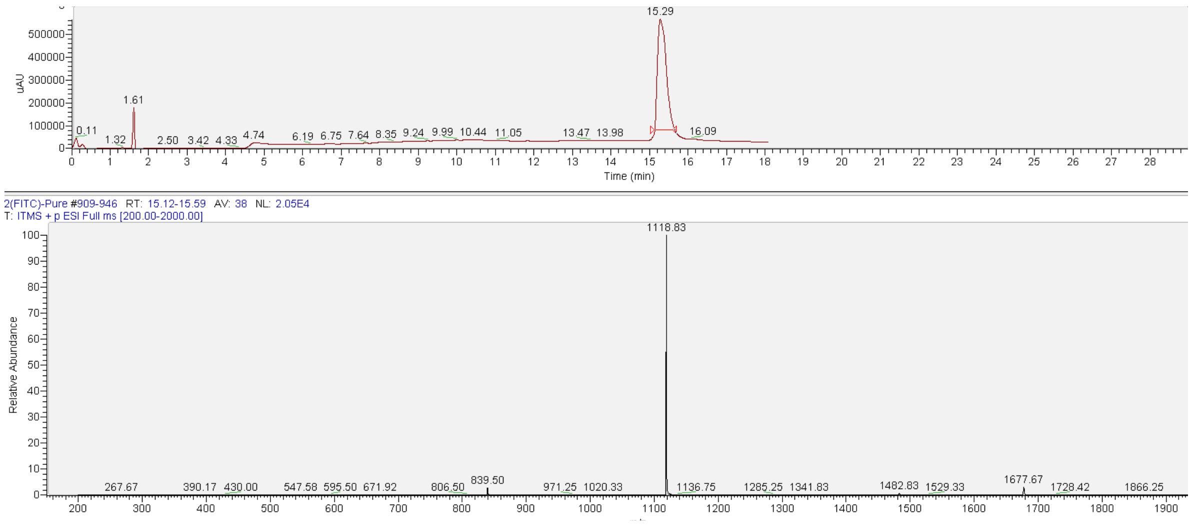

3(FITC)_pure

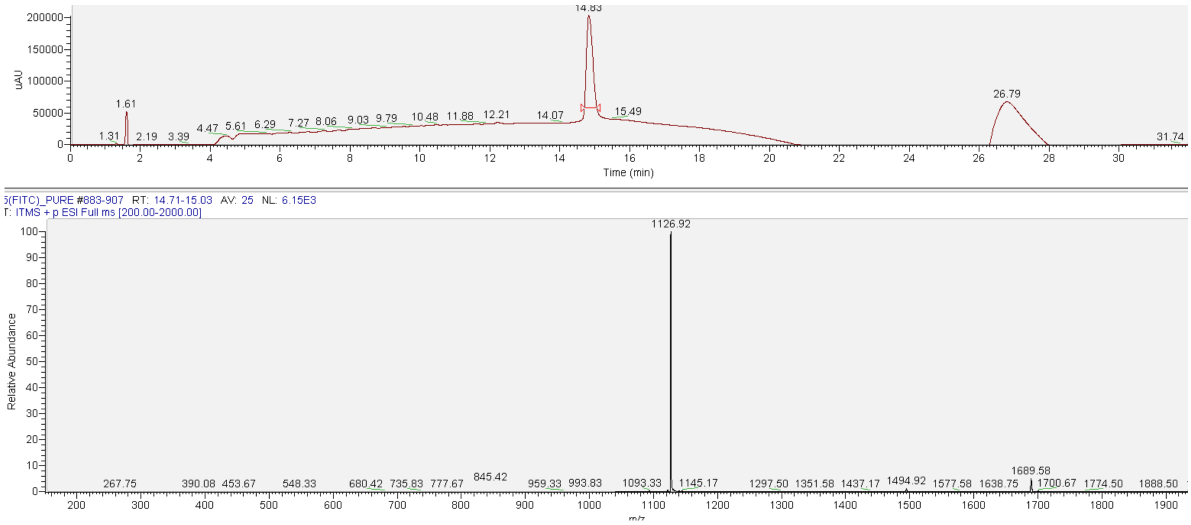

4(FITC)_pure

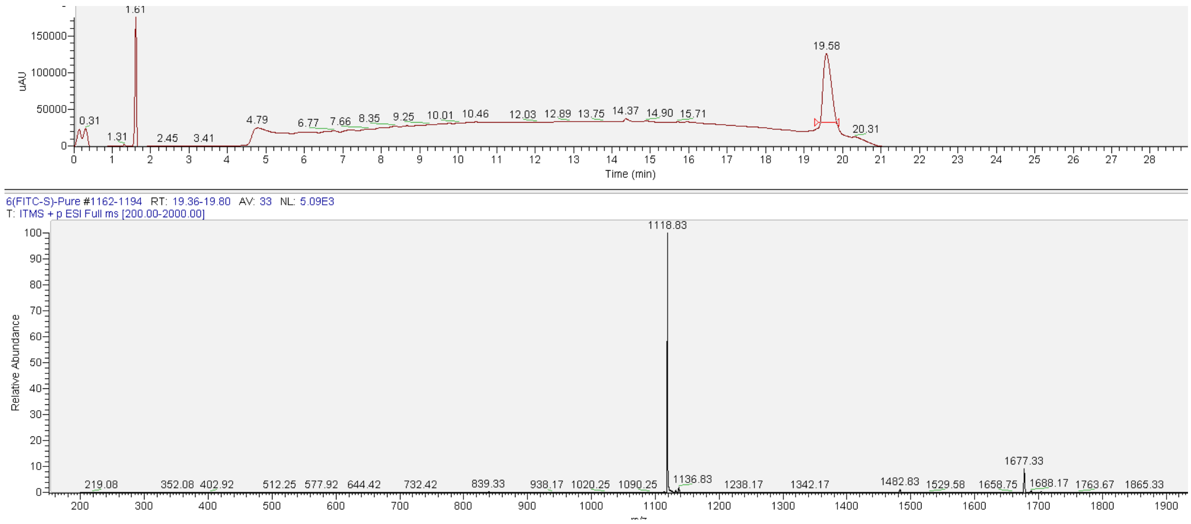

5(FITC)_pure

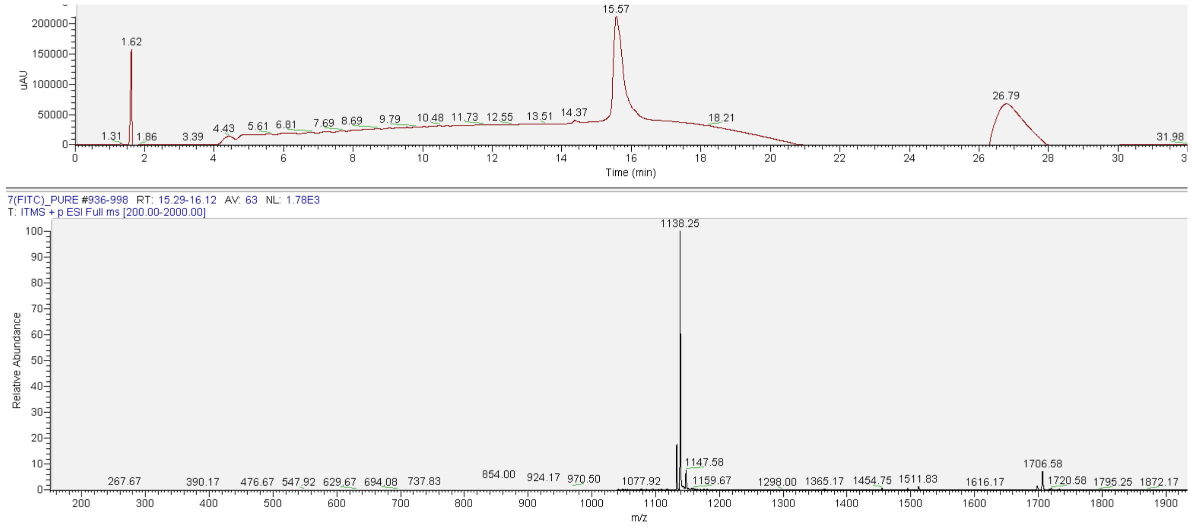

6(FITC)_pure

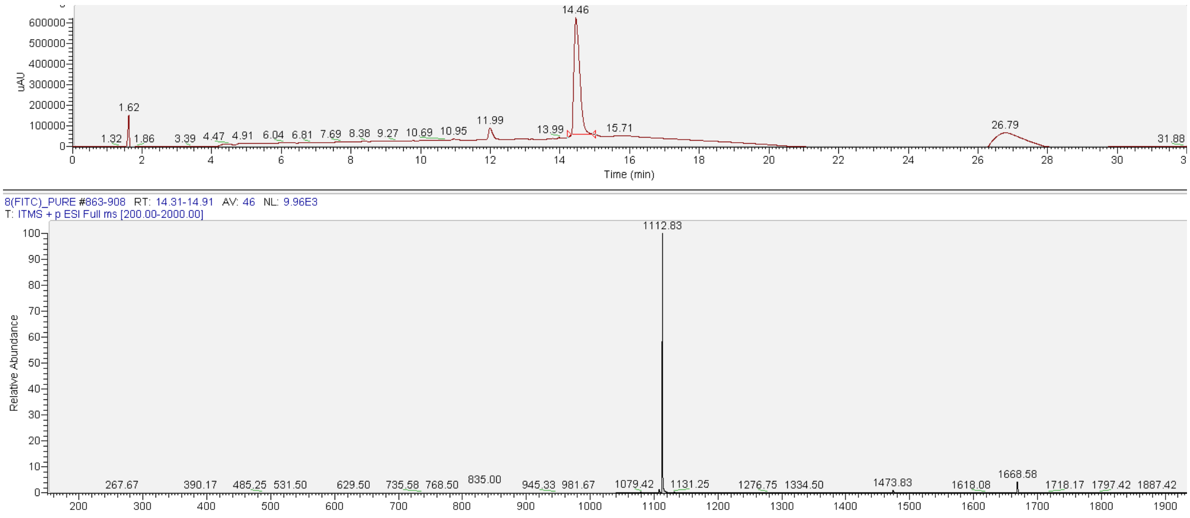

7(FITC)_pure

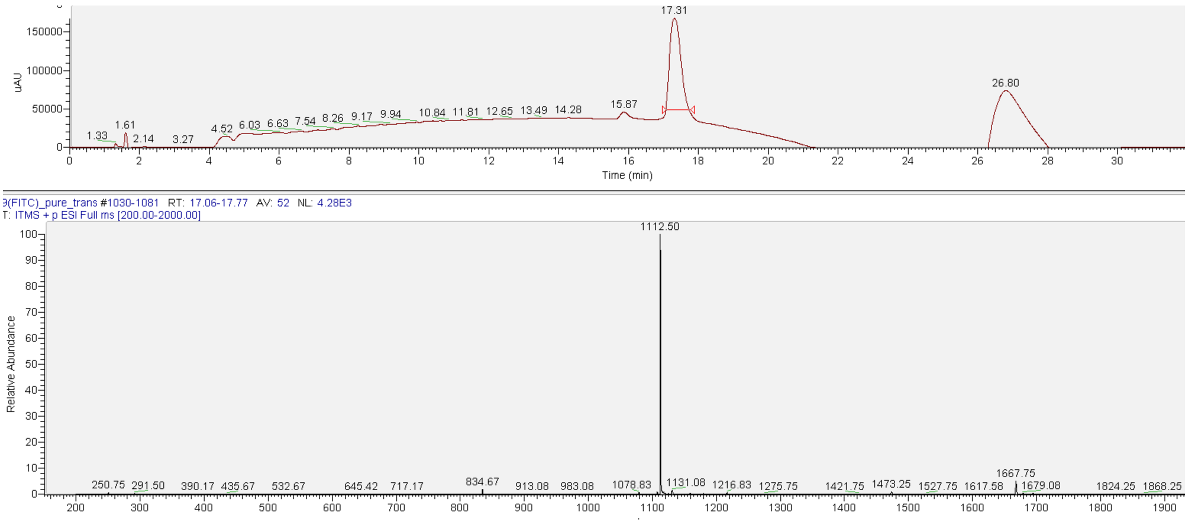

8(FITC)_pure

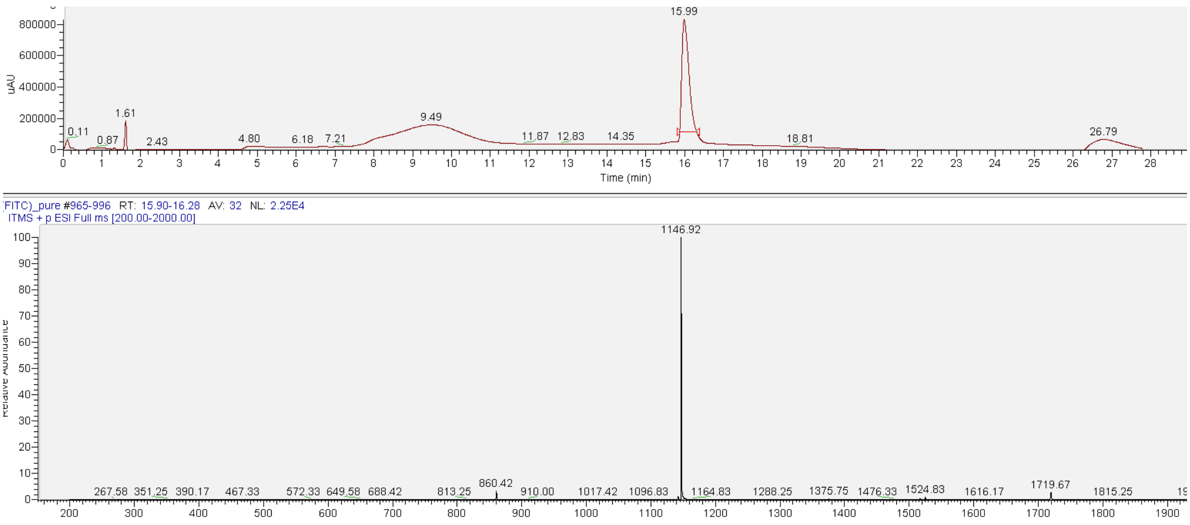

9(FITC)_pure

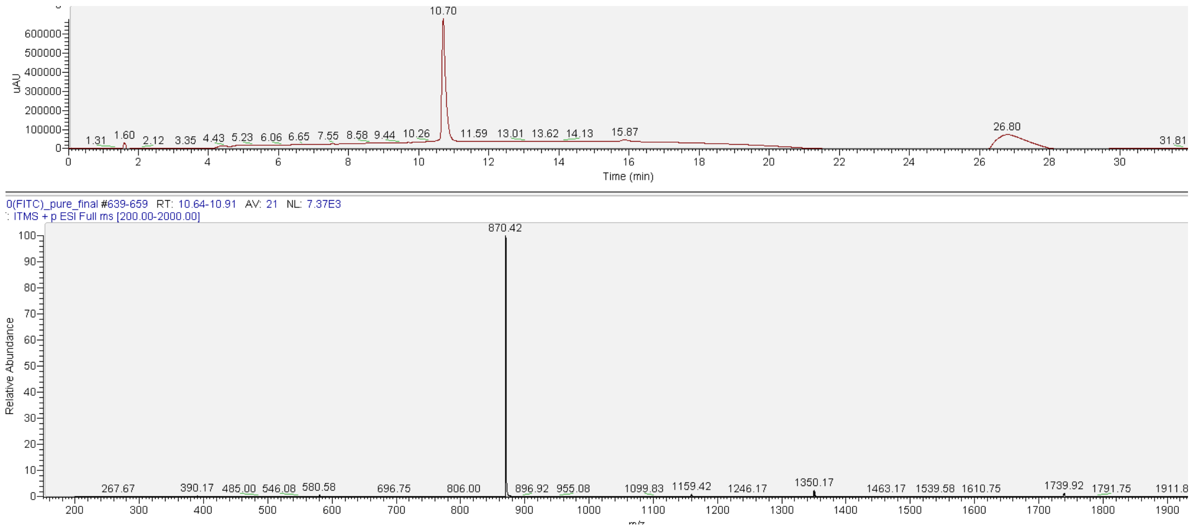

10(FITC)_pure

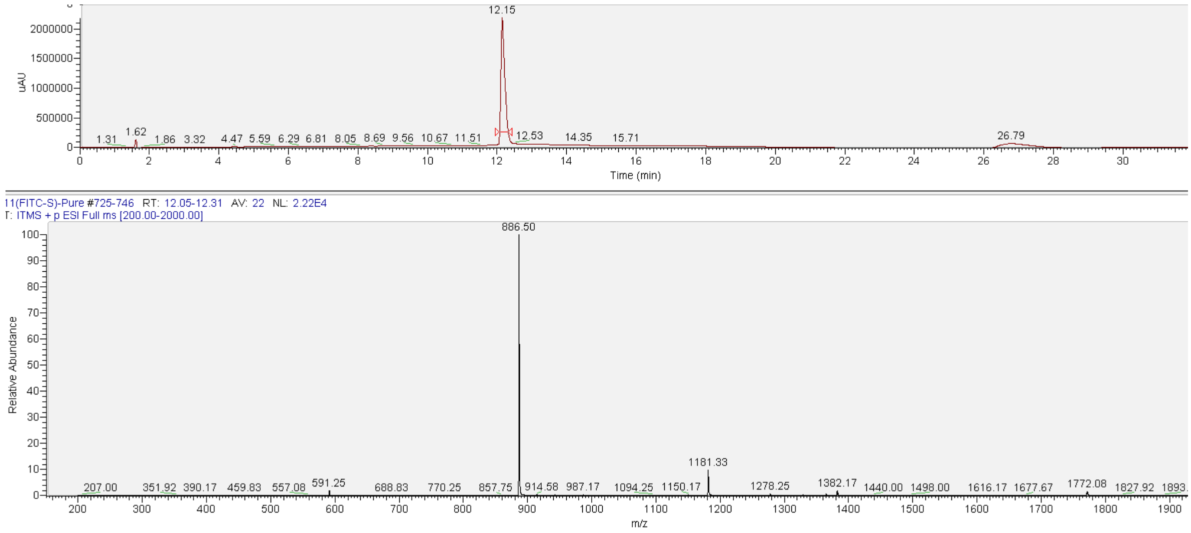

11(FITC)_pure

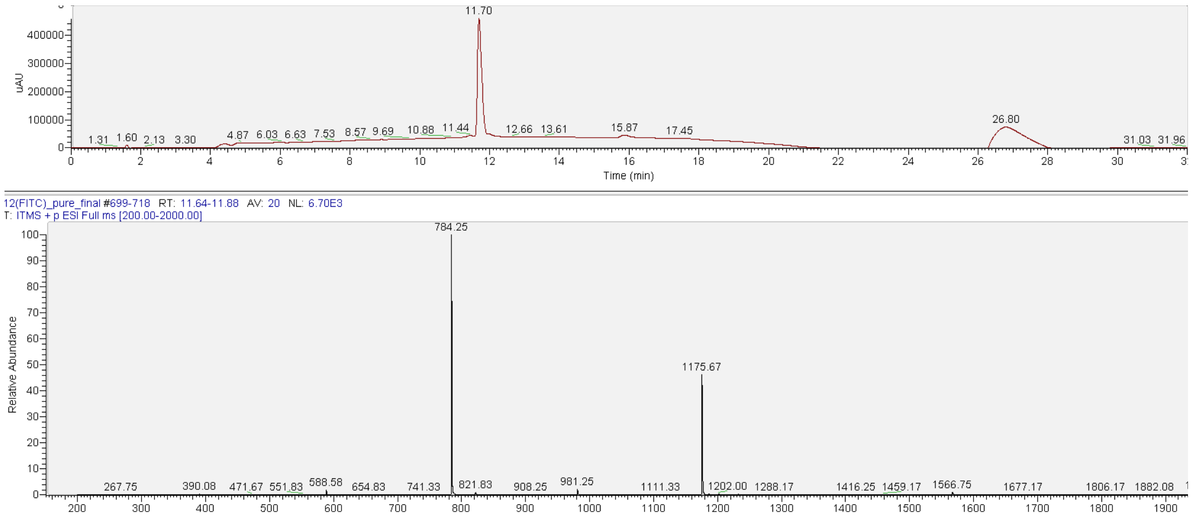

12(FITC)_pure

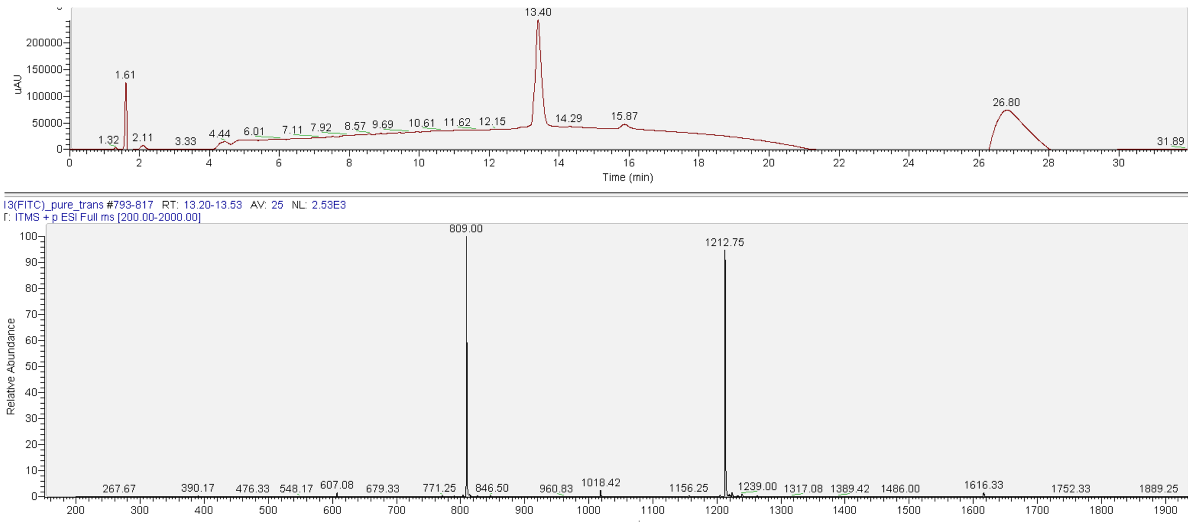

13(FITC)_pure

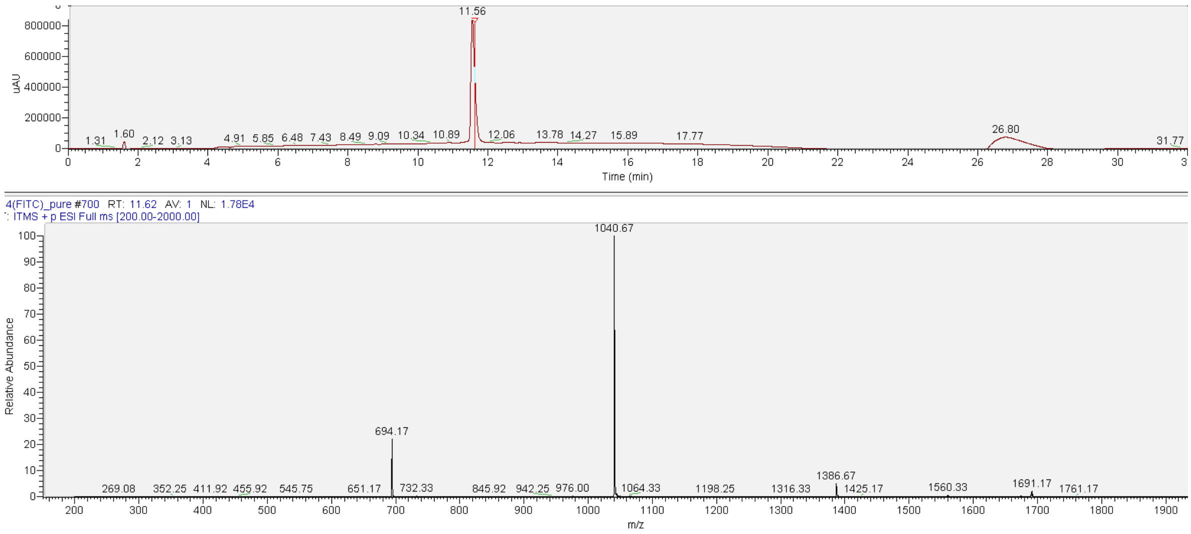

14(FITC)_pure

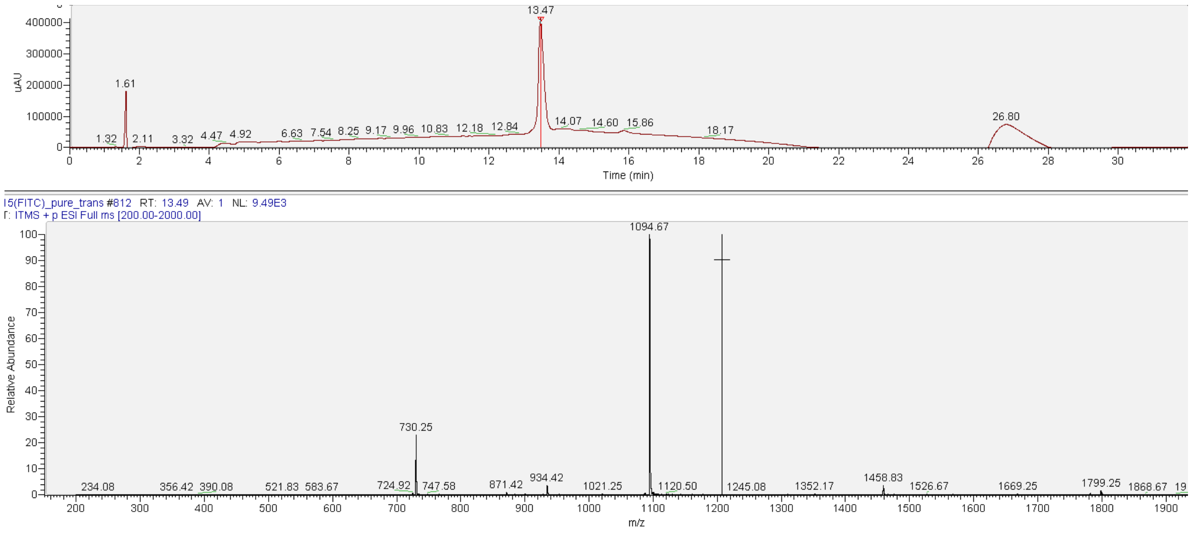

15(FITC)_pure

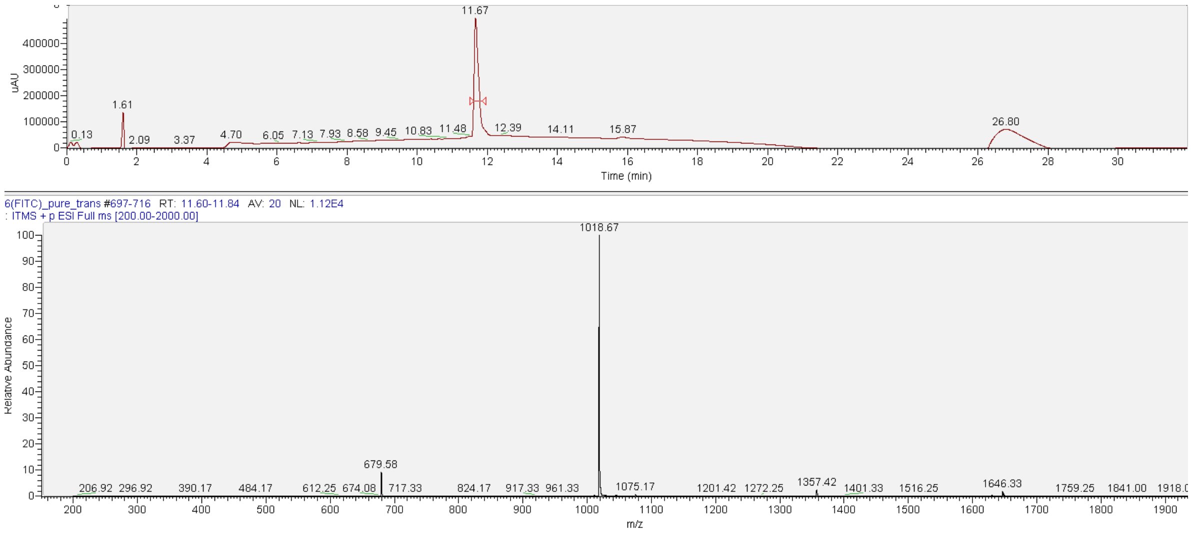

Scrambled(FITC)_pure

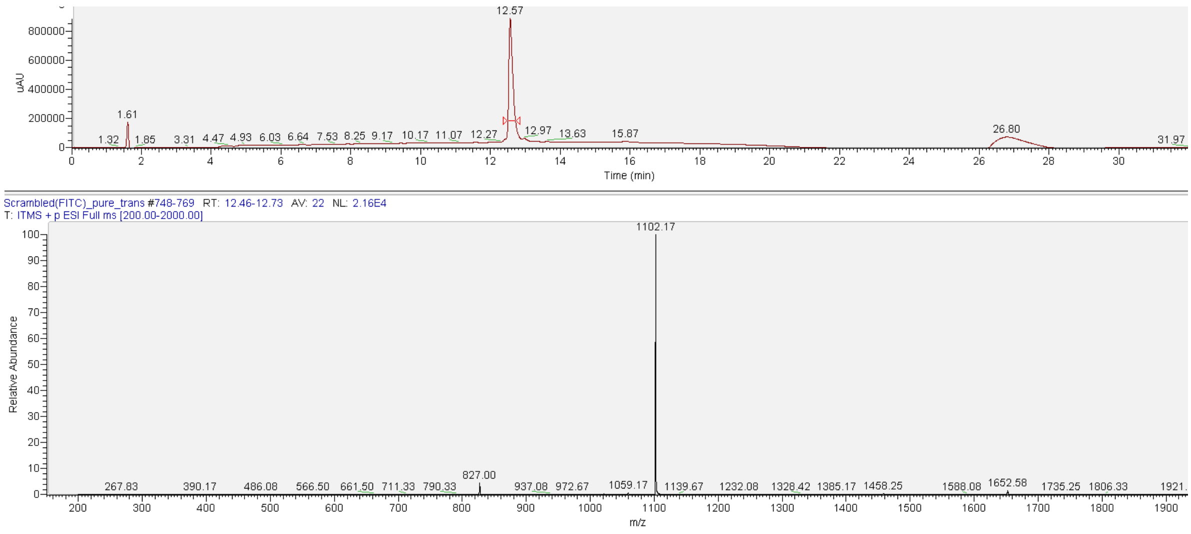

Negative Stapled(FITC)_pure

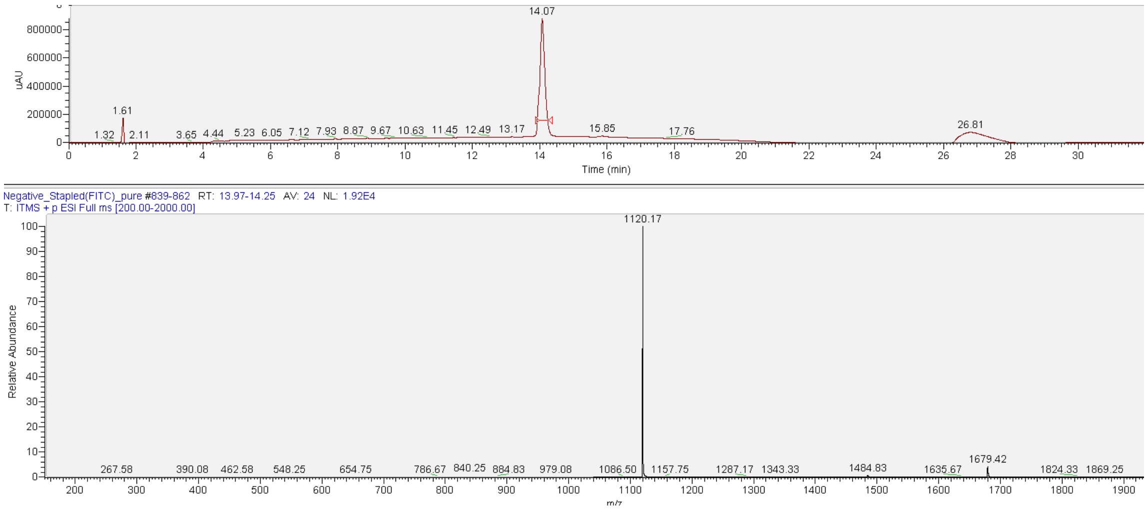

G-link(FITC)_pure

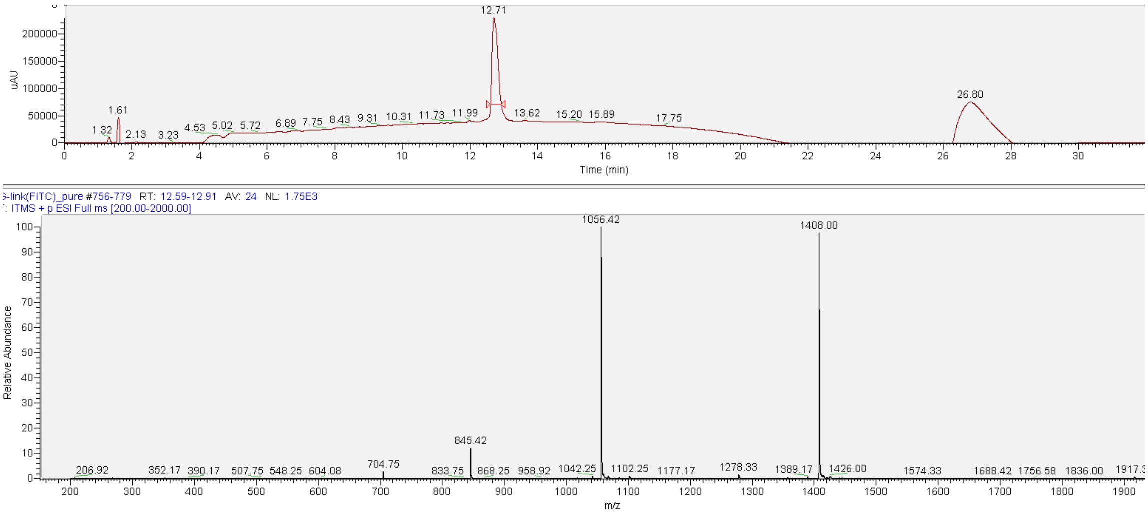

D30E(FITC)_pure

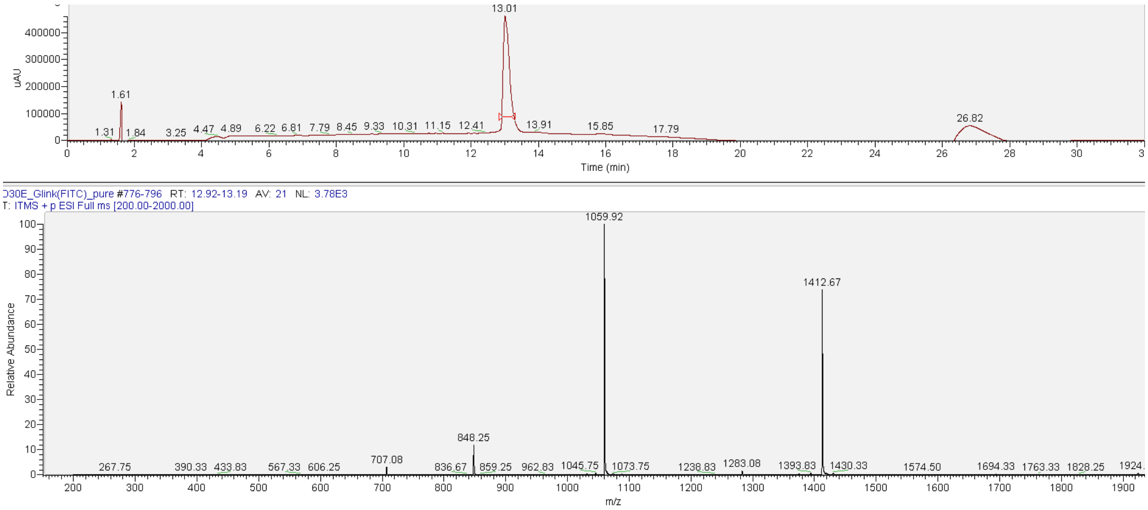

G-link Stapled(FITC)_pure

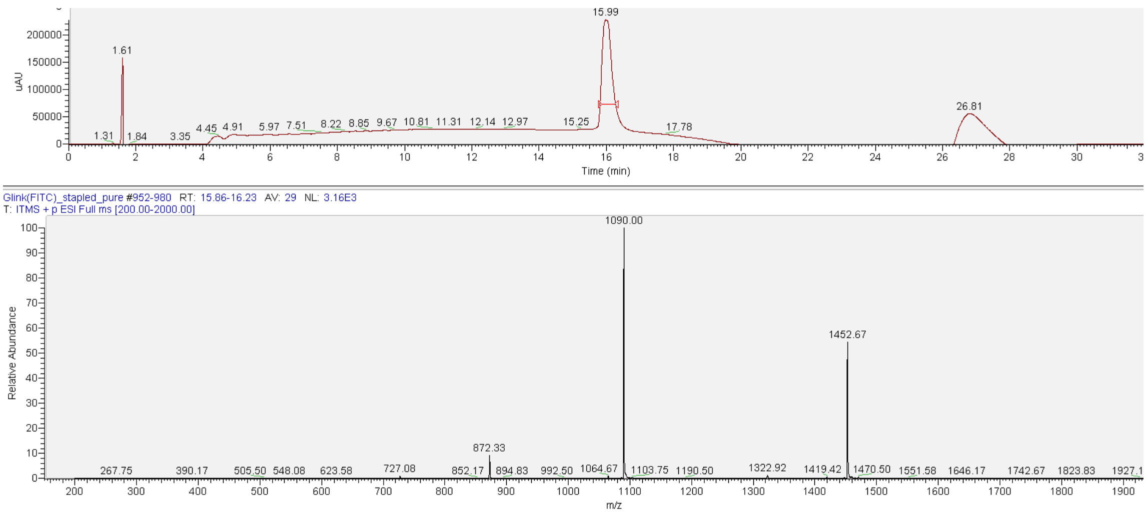

1(C-terminal_FITC)_pure

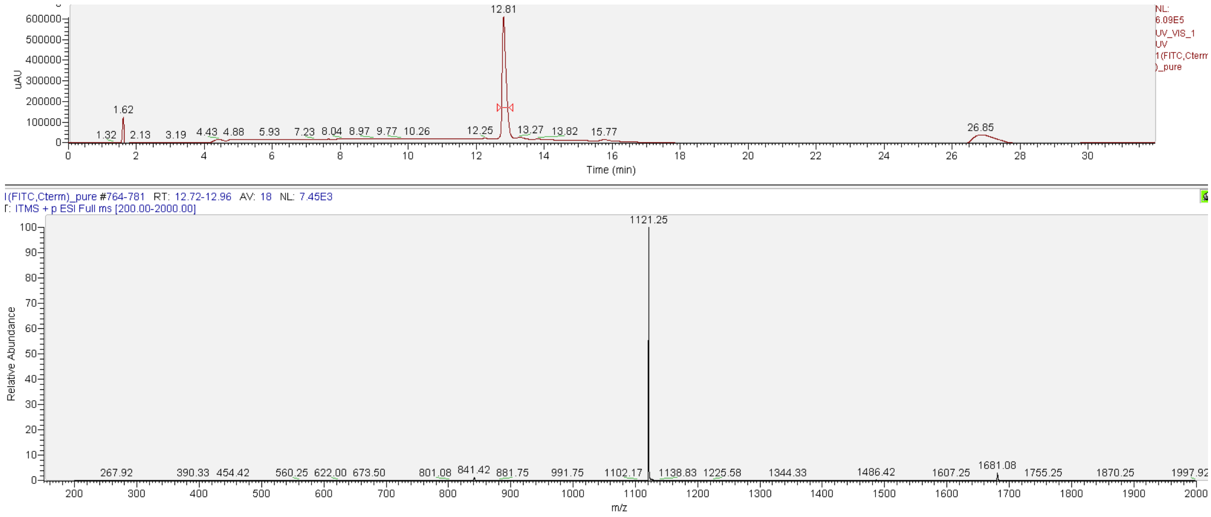

Scrambled(C-terminal_FITC)_pure

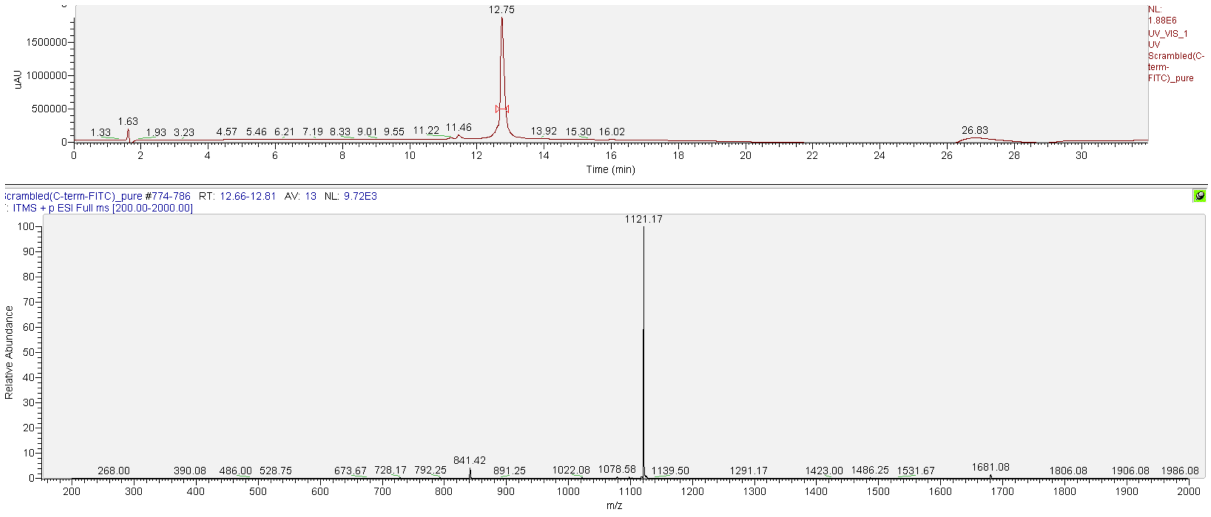

Supplement: Supplementary file 1 — Appendix S1. Supporting Information [file PEP2-113-0-s001.pdf]
